# A blueprint of ectoine metabolism from the genome of the industrial producer *Halomonas elongata* DSM 2581^T^

**DOI:** 10.1111/j.1462-2920.2010.02336.x

**Published:** 2011-08

**Authors:** Karin Schwibbert, Alberto Marin-Sanguino, Irina Bagyan, Gabriele Heidrich, Georg Lentzen, Harald Seitz, Markus Rampp, Stephan C Schuster, Hans-Peter Klenk, Friedhelm Pfeiffer, Dieter Oesterhelt, Hans Jörg Kunte

**Affiliations:** 1 Materials and Environment Division, Federal Institute for Materials Research and Testing (BAM)Berlin, Germany; 2 Department of Membrane Biochemistry, Max Planck Institute of BiochemistryMartinsried, Germany; 3 Research and Development DivisionBitop AG, Witten, Germany; 4 Department of Vertebrate Genomics, Max Planck Institute for Molecular GeneticsBerlin, Germany; 5 Computing Center (RZG) of the Max-Planck-Society, Max Planck Institute of Plasma PhysicsGarching, Germany; 6 Department of Biochemistry and Molecular Biology, Pennsylvania State University, University ParkPennsylvania, USA; 7 Microbiology, German Collection of Microorganisms and Cell Cultures (DSMZ)Braunschweig, Germany

## Abstract

The halophilic γ-proteobacterium *Halomonas elongata* DSM 2581^T^ thrives at high salinity by synthesizing and accumulating the compatible solute ectoine. Ectoine levels are highly regulated according to external salt levels but the overall picture of its metabolism and control is not well understood. Apart from its critical role in cell adaptation to halophilic environments, ectoine can be used as a stabilizer for enzymes and as a cell protectant in skin and health care applications and is thus produced annually on a scale of tons in an industrial process using *H. elongata* as producer strain. This paper presents the complete genome sequence of *H. elongata* (4 061 296 bp) and includes experiments and analysis identifying and characterizing the entire ectoine metabolism, including a newly discovered pathway for ectoine degradation and its cyclic connection to ectoine synthesis. The degradation of ectoine (doe) proceeds via hydrolysis of ectoine (DoeA) to Nα-acetyl-l-2,4-diaminobutyric acid, followed by deacetylation to diaminobutyric acid (DoeB). In *H. elongata*, diaminobutyric acid can either flow off to aspartate or re-enter the ectoine synthesis pathway, forming a cycle of ectoine synthesis and degradation. Genome comparison revealed that the ectoine degradation pathway exists predominantly in non-halophilic bacteria unable to synthesize ectoine. Based on the resulting genetic and biochemical data, a metabolic flux model of ectoine metabolism was derived that can be used to understand the way *H. elongata* survives under varying salt stresses and that provides a basis for a model-driven improvement of industrial ectoine production.

## Introduction

Concentrated salt solutions like salt or soda lakes, coastal lagoons or human-made salterns are extreme environments, inhabited by only a few forms of higher life, but usually maintain dense microbial populations comprising species from all three domains of life (Oren, 2002; 2008;Butinar *et al*., 2005). Global salt deposits show that evaporation of marine saltwater and the development of hypersaline habitats has been an ongoing process for millions of years, providing ample time for the evolution of halophilic *Bacteria* and *Archaea* that can flourish at high salt concentrations. Halophilic *Bacteria* and *Archaea* have developed two basically different osmoregulatory mechanisms to cope with ionic strength and the considerable water stress, namely the ‘salt-in-cytoplasm’ mechanism and the organic osmolyte mechanism (Oren, 2002). Organisms following the salt-in-cytoplasm mechanism adapt the interior protein chemistry of the cell to high salt concentration (Lanyi, 1974; Dennis and Shimmin, 1997; Kennedy *et al*., 2001; Tebbe *et al*., 2005). The osmotic adjustment of the cell can be achieved by raising the salt concentration (KCl) in the cytoplasm according to the environmental osmolarity. In contrast, microorganisms applying the organic osmolyte mechanism keep their cytoplasm, to a large extent, free of KCl and the design ofthe cell's interior remains basically unchanged. Instead, organisms of this group accumulate highly water-soluble organic compounds, in order to maintain an osmotic equilibrium with the surrounding medium (Roberts, 2006). These molecules do not disturb the cell's metabolism, even at high cytoplasmic concentrations, and are thus aptly named ‘compatible solutes’ (Brown, 1976).

The predominant compatible solutes in halophilic *Bacteria* are the amino acid derivatives glycine-betaine and ectoine (Roberts, 2006; Oren, 2008). Most chemoheterotrophic *Bacteria* can readily use glycine-betaine as a compatible solute if it is available in the environment. However, only a few are capable of *de novo* synthesis of glycine-betaine (Nyyssölä*et al*., 2000). Far more prokaryotes synthesize the aspartate derivative ectoine (1,4,5,6,tetra-2-methyl-4-pyrimidonecarboxylic acid) as their main compatible solute (Galinski *et al*., 1985; Severin *et al*., 1992; Roberts, 2006), which can also be utilized as an energy source by halophilic bacteria such as *Halomonas elongata* and *Chromohalobacter salexigens* (Vargas *et al*., 2006). Compatible solutes are beneficial for bacterial cells not only as osmoregulatory solutes, but also as protectants of proteins by mitigating detrimental effects of freezing, drying and high temperatures (Lippert and Galinski, 1992; Borges *et al*., 2002). The beneficial effect is explained by the unfavourable interaction of compatible solutes with the protein's peptide backbone. The lower affinity of compatible solutes, compared with water, for the protein surface is termed the osmophobic effect and results in a thermodynamic force that contributes to protein folding and increased protein stability (Bolen and Baskakov, 2001; Bolen, 2004). Ectoine possesses additional protective properties compared with other compatible solutes, and stabilizes even whole cells against stresses such as UV radiation or cytotoxins (Buommino *et al*., 2005; Furusho *et al*., 2005; Kanapathipillai *et al*., 2005; Kolp *et al*., 2006). It also protects against nanoparticle-induced inflammation in lung epithelia (Sydlik *et al*., 2009), and damage to the small bowel from ischaemia and reperfusion injury (Wei *et al*., 2009). Its protective properties make ectoine a valuable compound, which is marketed in health care and skin care products. Ectoine is therefore produced annually on a scale of tons by industry in a biotechnological process with the halophilic γ-proteobacterium *H. elongata* used as producer strain (Vreeland *et al*., 1980; Lentzen and Schwarz, 2006).

*Halomonas elongata*, which can tolerate salt concentrations well above 10% (1.7 M) NaCl, synthesizes ectoine via a pathway utilizing enzymes specified by three genes, *ectABC* (Göller *et al*., 1998). Cells do not rely only on *de novo* synthesis of ectoine for adaptation to high saline environments, but can also take up compatible solutes or precursors thereof from the medium. To enable solute uptake, *H. elongata* is equipped with a set of compatible solute transporters (Kunte, 2004) of which only one accepts ectoine as a substrate, namely, the ectoine-specific transporter TeaABC (Grammann *et al*., 2002; Tetsch and Kunte, 2002; Kuhlmann *et al*., 2008). TeaABC is not only required for the osmoregulatory accumulation of external ectoine, but also counterbalances an unknown system responsible for excreting endogenous ectoine to the medium (Grammann *et al*., 2002). Discovery of this system was based on the finding that strains of *H. elongata* with an inoperable TeaABC transporter constantly release ectoine to the surrounding medium. Despite this, cells of this mutant are able to keep the internal ectoine concentration at the same level as the wild-type strain. Apparently, the mutation of *teaABC* not only causes excretion of ectoine to the medium but also results in overproduction of ectoine. This observation led to the hypothesis that TeaABC might be linked to the regulation of ectoine synthesis (Kunte, 2006) and helped to design an ectoine production strain of *H. elongata* with higher productivity in ectoine synthesis than the wild-type strain (Kunte *et al*., 2002). However, the mechanism of ectoine excretion is still unknown. Because of the industrial importance of ectoine and its producer organism *H. elongata*, it is necessary to gain a deeper insight into ectoine transport across the cytoplasmic membrane, ectoine metabolism and the metabolic fluxes in *H. elongata* in order to increase ectoine production, e.g. by metabolic engineering (Marin-Sanguino *et al*., 2009). To attain deeper insight, a joint project of industry and research was established to sequence and analyse the genome of *H. elongata* DSM 2581^T^. The genome sequence helped to elucidate the degradation pathway of ectoine, which is presented in this paper for the first time. Furthermore, we provide a comparative analysis of the genes involved in ectoine synthesis and ectoine degradation from this genome with corresponding genes from microorganisms found in marine and saline environments and in soil. Finally, using a systems biology approach, a metabolic model on the genome data from *H. elongata* is introduced. This provides a basis for a model-driven improvement of ectoine production.

## Results and discussion

### Genome organization

The genome of *H. elongata* type strain DSM 2581^T^ consists of a single chromosome of 4 061 296 bp (Table 1) with a high GC content (63.6%). For this chromosome, a total of 3473 protein-coding genes were predicted using the Reganor program (McHardy *et al*., 2004), which integrates data obtained from Critica (Badger and Olsen, 1999) and Glimmer (Delcher *et al*., 1999).

**Table 1:** *H. elongata* DSM 2581T genome summary

Chromosomes	1
DNA, total number bases	4 061 296
Coding density (%)	87.1%
G + C content (%)	63.6%
Genes, total number	3 555
Protein coding genes	3 473
Total RNA genes	82
5S rRNA	4
16S rRNA	4
23S rRNA	4
tRNA genes	68
Other RNA genes	2
Genes with assigned EC number	1 265

The most prominent duplications are the four rRNA operons. The 16S rRNA and 5S rRNA sequences are identical in all four operons, while the 23S rRNA sequences contain nine polymorphic sites. There are only seven other large repeats (1.4–3.0 kb with approximately 88–99% sequence identity). In all cases, these repeats represent duplications of genes, e.g. of enzymes that participate in central intermediary metabolism. In some cases, the duplicated genes are affected by a frameshift or diverge beyond the end of the duplicated region. The *H. elongata* chromosome shows a typical GC skew plot with two inflections (data not shown), indicating the origin of replication and the termination point (position 1835573). The ectoine biosynthesis genes *ectABC* are only 5 kb from the termination point.

### Genome comparison

A comparison of the predicted protein sequence set of *H. elongata* with the NR database (downloaded from NCBI October 4, 2009) emphasizes a very close relationship of *H. elongata* with *C. salexigens*, a halophilic γ-proteobacterium of the family Halomonadaceae (Arahal *et al*., 2001). About half of the proteins could be reliably assigned at the species level (1672 out of 3473) using protein BLAST (Altschul *et al*., 1997) and MEGAN (Huson *et al*., 2007). The vast majority of the assigned proteins are related to *C. salexigens* (1544 assignments, corresponds to 92%). The few remaining MEGAN assignments are relatively equally distributed among a number of different species with *Marinobacter* sp. ELB17 (2.6%) dominating any other species (< 1%). For an overview, see Fig. S1. For 290 sequences, no assignment was reported to any taxon by MEGAN because no significant hit to the NR database was found.

Approximately two-thirds of the *H. elongata* proteins have an ortholog in *C. salexigens* as indicated by bidirectional best BLAST results (2367 proteins, 68.1%). They are very closely related, with an average of 69% sequence identity. Gene order is also well conserved. On closer examination of the set of *H. elongata* proteins that have an ortholog in *C. salexigens*, it was found that when two genes encoding such orthologous proteins are the nearest genetic neighbours in *H. elongata* then their corresponding partners in *C. salexigens* are also the nearest genetic neighbours in 70% of the cases. This high level of synteny is also evident from a MUMmer alignment of the two chromosomal sequences (Fig. S2). This results in a prominent X-alignment. Such X-alignments have been described for several interspecies comparisons (Eisen *et al*., 2000). The prominence of the X-alignment may indicate a close relationship between the two species, which is astonishing as both organisms are classified into distinct genera.

We used the Metanor tool of the GenDB genome annotation system (Meyer *et al*., 2003) for automatic function prediction. For enzymes, these were cross-checked with the data obtained by the PRIAM program (Claudel-Renard *et al*., 2003). A total of 1265 complete or partial EC numbers were assigned. Central metabolism and the biosynthetic pathways related to ectoine biosynthesis and degradation were manually curated in detail.

*Halomonas elongata* contains a complete set of ribosomal proteins. There are tRNA ligases for 19 of the 20 canonical amino acids. Asparagine (Asn) is not loaded as Asn but as aspartate with subsequent amidation to Asn by a *gatABC-*encoded enzyme.

To further analyse the protein set from *H. elongata*, we assigned cluster of orthologous groups of proteins (COGs) using the eggNOG 2.0 dataset as a reference (Muller *et al*., 2010). The assignment procedure, using an in-house script to analyse BlastP results, is described in *Experimental procedures* and in *Supporting information*.

We found a number of high-occupancy COGs that contain many different proteins from *H. elongata* (Table S1). Six COGs have more than 20 proteins of which COG0583 is most highly occupied with 57 members. The 20 most highly occupied COGs belong to four functional classes: (i) transcription regulators (six COGs), (ii) ‘general function’ enzymes (five COGs), (iii) transporters (seven COGs) and (iv) two-component systems (two COGs).

The most highly occupied COG0583 contains *lysR* family transcription regulators. The five high-occupancy COGs with ‘general function’ enzymes code for short-chain alcohol dehydrogenases, aldehyde dehydrogenases, acetyltransferases, methyltransferases and FAD-dependent oxidoreductases. One of the acetyltransferases in the frequent COG0454 is EctA, the first enzyme of the ectoine biosynthesis pathway. The seven high-occupancy COGs with transporters include the three subunits of TRAP transporters (COG1638, COG1593, COG3090, TRAP-type C4-dicaroxylate transport system, periplasmic component, large and small permease component). The *teaABC* ectoine transporter belongs to this set of COGs. The other frequently occurring transporters are MFS superfamily permeases and DMT superfamily permeases as well as two subunits of ABC-type transporters (ATPase, permease).

We searched for COGs, which are preferentially carried by halophilic/marine bacteria by comparing the proteins of 3 halophiles and 10 marines to 14 non-halophilic species (Supporting information, Table S2). Among the 97 COGs identified by this approach were many secondary transport systems that are thought to be dependent on Na^+^ symport, and sodium-proton exchangers. The transport systems that can be mainly found in halophiles belong to the NSS family and the TRAP family of transporters. The distribution of TRAP transporters was analysed by Mulligan and colleagues (2007) and they found that these uptake systems are extensively used in marine bacteria. There is evidence that TRAP transporters are powered by sodium symport (Mulligan *et al*., 2007) and perhaps the utilization of sodium-dependent transporters could be advantageous for halophilic bacteria that have a sodium gradient across their membrane. Members of the NSS family of transporters can be found in *Eukarya*, *Bacteria* and *Archaea* and transport nitrogenous substances (Beuming *et al*., 2006; Quick *et al*., 2006). In bacteria, NSS transporters catalyse the high-affinity uptake of amino acids by a sodium-symport mechanism (Androutsellis-Theotokis *et al*., 2003). Again, the preference of marine bacteria for these transporters can be explained by their dependency on sodium for transport. Both the NSS and TRAP transporter, also have a high affinity for their substrates (Androutsellis-Theotokis *et al*., 2003; Chae and Zylstra, 2006; Kuhlmann *et al*., 2008), which might be required in the marine environment with sometimes low solute concentrations.

High-salt adaptation may result in protein adaptation, e.g. by adjusting protein pI values. We analysed whether *H. elongata* has an unusual average pI when compared with each of the 27 organisms selected for identification of halophile-specific COGs (Supporting information, Table S3). If there is any pI shift in *H. elongata* proteins, the shift is only very slight and towards the acidic direction. These results indicate that an overall acidic proteome is not required for salt adaptation of halophilic bacteria employing the organic osmolyte mechanism.

### Ectoine synthesis

**Fig. 1:**
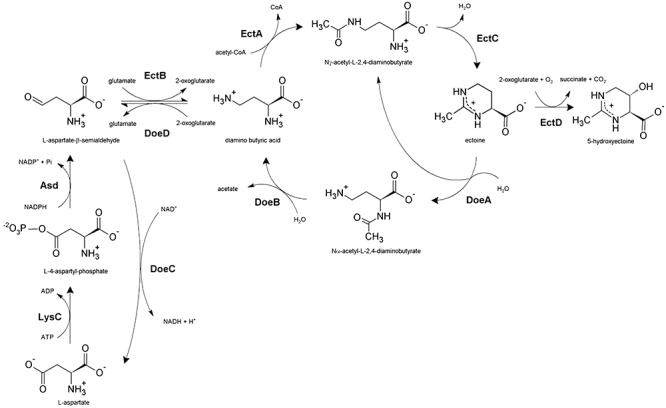
Metabolic pathway of the compatible solute ectoine in *H. elongata*. The degradation pathway is based on genetic and chromatographic analysis carried out in this study. Shown here is the hydrolysis of ectoine that leads directly to Nγ- and Nα-acetyl-l-2,4-diaminobutyric acid. For more details on DoeA activity see text. The depicted ectoine biosynthesis pathway is according to studies previously published (Peters *et al*., 1990; Göller *et al*., 1998; Ono *et al*., 1999). LysC: aspartate kinase; Asd: β-aspartate-semialdehyde-dehydrogenase; EctB: l-2,4-diaminobutyric acid transaminase; EctA: l-2,4-diaminobutyric acid Nγ-acetyltransferase; EctC: ectoine synthase; EctD: ectoine hydroxylase; DoeA: ectoine hydrolase; DoeB: Nα-acetyl-l-2,4-diaminobutyric acid deacetylase; DoeD: l-2,4-diaminobutyric acid transaminase; DoeC: aspartate-semialdehyde dehydrogenase.

*Halomonas elongata* synthesizes ectoine (1,4,5,6,tetra-2-methyl-4-pyrimidonecarboxylic acid) as its main compatible solute (Severin *et al*., 1992). Ectoine is synthesized from aspartate-semialdehyde, the central intermediate in the synthesis of amino acids belonging to the aspartate family (Fig. 1). Ectoine formation comprises three enzymatic steps (Peters *et al*., 1990; Ono *et al*., 1999). First, aspartate-semialdehyde is transaminated to 2,4-diaminobutyric acid (DABA) with glutamate as amino-group donor. The transamination is catalysed by DABA transaminase (EctB). Then, an acetyl group is transferred to DABA from acetyl-CoA by DABA-Nγ-acetyltransferase (EctA) in order to synthesize Nγ-acetyl-l-2,4-diaminobutyric acid. Finally, ectoine synthase (EctC) catalyses the cyclic condensation of Nγ-acetyl-l-2,4-diaminobutyric acid, which leads to the formation of ectoine. Under certain stress conditions (e.g. elevated temperatures) *H. elongata* converts some of the ectoine to 5-hydroxyectoine by ectoine hydroxylase (EctD) (Inbar and Lapidot, 1988; Wohlfarth *et al*., 1990).

**Fig. 2:**
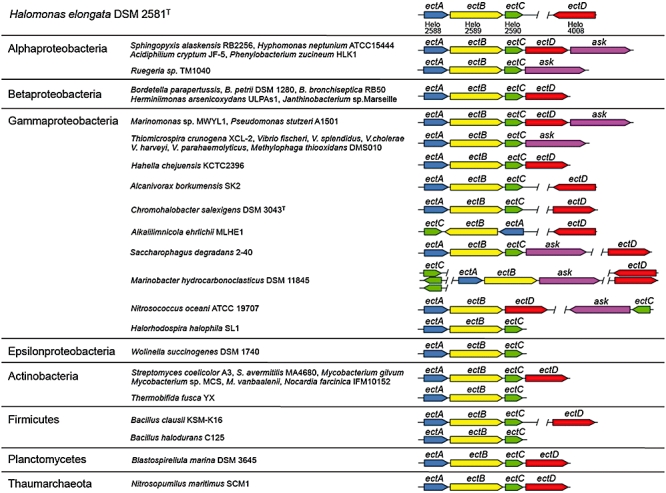
Ectoine synthesis gene organization in *H. elongata* and other prokaryotes. Shown are the *ect* genes from the subdivisions of the proteobacteria, the phyla Actinobacteria, Firmicutes, Planctomycetes and Thaumarchaeota. Only ectoine synthesis genes from prokaryotes with known genome sequences are depicted. l-2,4-diaminobutyric acid Nγ-acetyltransferase genes (*ectA*) are blue, diaminobutyric acid transaminase genes (*ectB*) are yellow, ectoine synthase (*ectC*) genes are green, ectoine hydroxylase genes (*ectD, ectE*) are red and aspartate kinase genes (*ask*) are pink. The accession numbers of all *ect* and *ask* genes are listed in Table S6.

The genes encoding the enzymes for ectoine *de novo* synthesis in *H. elongata* were identified by transposon mutagenesis, and the nucleotide sequence of *ectAB* as well as a partial sequence of *ectC* were published in 1998 (Göller *et al*., 1998). The genomic region containing these ectoine biosynthesis genes (*ectA: Helo_2588, ectB: Helo_2589, ectC: Helo_2590*) is shown in Fig. 2. The *ectD* gene (*Helo_4008*) encoding the hydroxylase for hydroxyectoine synthesis (Prabhu *et al*., 2004; Bursy *et al*., 2007) is located apart from the *ectABC* cluster. Immediately downstream of the 414 nt comprising *ectC* gene a further ORF is located (*Helo_2591*) that is predicted to encode a transcriptional regulator of the AraC family.

The *ectA* gene encodes a 192-residue protein with a calculated molecular mass of 21.2 kDa. According to the studies of Ono and colleagues (Ono *et al*., 1999), DABA-acetyltransferase EctA displays a high specificity for its substrate DABA. EctA from *H. elongata* is a rather acidic protein with a calculated pI value of 4.8.

Similar acidic EctA proteins can be found in most of the marine and halophilic bacteria (e.g. *C. salexigens*, pI 5.3; *Bacillus halodurans*, pI 5.5; *Halorhodospira halophila*, pI 5.4; *Blastospirellula marina* pI 4.7) as well as in soil bacteria from the Actinomyces group (e.g. *Nocardia farcinica*, pI 5.0; *Mycobacterium gilvum*, pI 5.9; *Streptomyces coelicolor*, pI 5.0). In contrast, all but one of the remaining non-halophilic bacteria analysed in this study possess EctA proteins with a neutral or alkaline pI (e.g. *Bacillus clausii*, pI 7.5; *Pseudomonas stutzeri*, pI 8.0; *Wolinella succinogenes*, pI 9.0; *Bordetella parapertussis*, pI 8.4; *Phenylobacterium zucineum*, pI 8.9).

The *ectB* gene encodes a 421-residue protein with a molecular mass of 46.1 kDa, which requires K^+^ for its transaminase activity and for protein stability. Gel filtration experiments with purified protein from *H. elongata* indicate that the DABA aminotransferase EctB might form a homohexamer in the native state (Ono *et al*., 1999). The preferred amino group donor of EctB in the formation of DABA is glutamate, while in the reverse reaction DABA and γ-aminobutyrate are the preferred amino group donors to α-ketoglutarate.

The *ectC* gene encodes ectoine synthase, a 137-residue protein with a calculated molecular weight of 15.5 kDa and a pI value of 4.9. The EctC protein belongs to the enzyme family of carbon-oxygen lyases. *In vitro* experiments with purified EctC revealed that ectoine-synthase activity and affinity to its substrate are strongly affected by NaCl (Ono *et al*., 1999). N-acetylated amino acids having a carbon skeleton with one (ornithine derivatives) or two (lysine derivatives) atoms more than Nγ-acetyl-diaminobutyric acid are not suitable substrates for EctC. Galinski and co-workers, who described the ectoine biosynthetic pathway for the first time, demonstrated the reversibility of the ectoine synthase reaction when measured in crude cell extracts of *Halorhodospira* (formerly *Ectothiorhodospira*) *halochloris* (Peters *et al*., 1990). However, Ono *et al*. characterized purified EctC from *H. elongata* as an enzyme that is unable to carry out the reverse reaction from ectoine to Nγ-acetyl-diaminobutyric acid when ectoine was offered as substrate in the range of 10 mM to 1 M (Ono *et al*., 1999).

The *ectD* encoded ectoine hydroxylase consists of 332 amino acids and has a molecular weight of 37.4 kDa. The EctD protein is a member of an oxygenase subfamily within the non-heme-containing, iron (II)- and α-ketoglutarate-dependent dioxygenase superfamily. Ectoine hydroxylase was shown to catalyse the direct hydroxylation of ectoine to 5-hydroxyectoine (Bursy *et al*., 2007).

### Transcriptional regulation of *ectABC*

Recent studies on the transcriptional regulation of *ectABC* in *Bacillus pasteurii* and *Halobacillus halophilus* revealed that *ectABC* is organized as one operon (Kuhlmann and Bremer, 2002; Saum and Müller, 2008), while in the halophilic γ-proteobacterium *C. salexigens* the transcriptional organization of the *ect*-cluster turned out to be rather complex (Calderón *et al*., 2004). Calderón *et al*. mapped a total of five promoters regulating *ectABC* transcription. Two σ^70^-controlled promoters, one σ^s^-dependent promoter and a promoter of unknown specificity are located upstream of *ectA*, while a fifth promoter was found upstream of *ectB*.

**Fig. 3:**
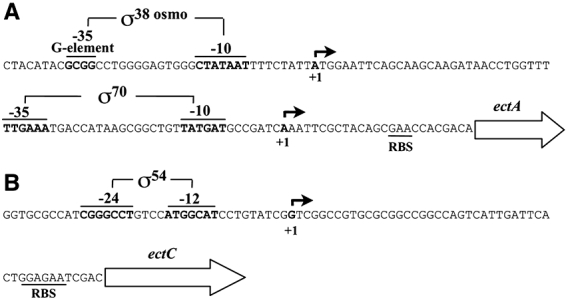
Transcription initiation sites and putative promoters of the ectoine synthesis genes *ectABC*. Nucleotide sequences of the *ectA* (A) and *ectC* (B) promoter regions. Arrows indicate the transcription initiation sites (+ 1), which were mapped by RACE-PCR. The −35 and −10 sequences of the σ^70^ and σ^38^ promoters upstream of *ectA*, and the −24 and −12 sequences of the σ^54^ promoter upstream of *ectC* are written in bold. (B) An 18 bp sequence GCCCGCTGACCATT is located at −111 to −128 bp upstream of the *ectC* transcription initiation site that resembles the consensus sequence of σ^54^ promoters that are controlled by FleQ (not displayed).

To gain further information on the transcriptional regulation of the *ectABC* gene-cluster, we mapped the transcriptional initiation sites in *H. elongata* by RACE-PCR and found a different, but also complex promoter assembly. Two transcriptional initiation sites could be pinpointed in front of *ectA*, and one was mapped immediately upstream of *ectC* (Fig. 3). The two transcription initiation sites before *ectA* are located 25 and 92 bp, respectively, upstream from the *ectA* start codon. Inspection of the DNA sequence upstream of the first *ectA* initiation site (25 bp) revealed the presence of putative −10 and −35 sequences that resemble the binding site for the vegetative sigma factor σ^70^. The −10 and −35 sequences are separated by 17 bp, a typical spacing for promoters controlled by σ^70^. Upstream of the second initiation site (92 bp), a −10 DNA sequence was found that resembles σ^38^-controlled promoters. In addition to the −10 region, a so-called G-element exists at position −35. G-elements are characteristic for osmotically induced σ^38^ promoters (Lee and Gralla, 2004). RACE-PCR at the *ectC* gene mapped a transcription start point 47 bp upstream from the start codon (Fig. 3B). Upstream of the initiation site putative −12 and −24 sequences were found that are typical for σ^54^-controlled promoters. σ^54^-controlled promoters are often involved in transcription of nitrogen-regulated genes (Ausubel, 1984; Bordo *et al*., 1998). The two sequences of the putative σ^54^ promoter are appropriately spaced by four nucleotides. In addition, a sequence of 18 bp was found −111 to −128 bp upstream of the initiation site that resembles a consensus sequence required for transcription activation of some σ^54^ promoters controlled by FleQ (Hu *et al*., 2005). The transcriptional regulation of *ectABC* by an osmoregulated σ^38^ promoter and a σ^54^ promoter is in agreement with physiological observations made with other bacteria, such as *Corynebacterium glutamicum* and *H. halochloris*. In these organisms, it was shown that synthesis of the compatible solutes proline and glycine-betaine, respectively, is not only determined by salinity but also by nitrogen supply (Galinski and Herzog, 1990; Wolf *et al*., 2003).

### Organization of *ectABC* in different prokaryotes

The *ectABC* genes and the proteins for ectoine synthesis are very conserved among ectoine-producing bacteria. A study published recently by Lo and colleagues (2009) analysed the phylogenetic distribution of *ect* genes and showed that the prevalent organization of these genes is in a single cluster of at least three genes (*ectABC*), consistent with the analysis presented in this study. However, comparison of the *H. elongata* genome with other genomes revealed that the *ectABC* genes are not always organized in this way. A first analysis carried out by Vargas and co-workers with the genome of *C. salexigens* came to a similar result (Vargas *et al*., 2008). We extended this study and found that in *Nitrosococcus oceani, ectC* is located at a site different from *ectAB*. *Marinobacter hydrocarbonoclasticus* DSM 11845 [formerly *aquaeolei* VT8 (Márquez and Ventosa, 2005)] carries three *ectC* ORFs, but these are located at different sites within the genome and none are close to *ectAB*. In *Alkalilimnicola ehrlichii*, gene *ectC* is located downstream of *ectAB* but on the opposite strand. In all other genomes that were considered in this study, the *ectABC* components were clustered similar to *ectABC* from *H. elongata*. In summary, the way the *ect-*genes are organized in these organisms can be classified as follows (Fig. 2):

- *Bacillus halodurans, Ruegeria*[formerly *Silicibacter* (Yi *et al*., 2007)] sp. TM1040, and all *Vibrio* species analysed in this study possess only one single *ectABC* cluster and are missing *ectD* that encodes the ectoine hydroxylase.
- In *H. elongata* and two other members of the Oceanospirillales (*C. salexigens*, *Alcanivorax borkumensis* SK2), in γ-proteobacterium *Saccharophagus degradans*, and in *B. clausii*, the *ectABC* cluster and gene *ectD* can be found at separate sites within the genome. *C. salexigens* differs from *H. elongata* and the other members of this group in having a second *ectD*-like ORF named *ectE*. Similar to *ectD*, locus *ectE* is separated from *ectABC*.
- The majority of genomes that were checked in this study, including the chromosome of the crenarchaeote *Nitrosopumilus maritimus*, contain a single *ect*-cluster comprising the genes *ectABC* and *D*.

In roughly half of the genomes that were compared with the *H. elongata* genome an additional ORF (*ask*) can be found encoding a putative aspartate kinase. The ORF *ask* is located downstream of *ectB*, *ectC* or *ectD*. All bacterial genomes in this study that contain *ask* are equipped with at least one further aspartate kinase (LysC). It is therefore tempting to speculate whether *ask* next to the *ect* components is coding for a specific kinase involved in ectoine synthesis. *H. elongata* and its halophilic relative *C. salexigens* do not possess such an *ask*. They rely on only one type of aspartate kinase, LysC (*Helo_3742*), responsible for the synthesis of ectoine and the amino acids lysine, threonine and methionine.

Homology analysis of aspartate kinases by Lo and colleagues (2009) revealed a separation of two subhomology divisions, which are denoted ASKα and ASKβ. Amino acid sequence analysis showed that LysC from *H. elongata* belongs to the ASKβ homology division and is most closely related to the ASKβ aspartate kinase from *C. salexigens*. According to the allosteric-specifity grouping of ASKβ enzymes, LysC of *H. elongata* is sensitive to the allosteric regulation of Thr and Lys. The gene encoding LysC in *H. elongata* is associated with the genes *recA recX alaS lysC crsA tRNA*, which is a conserved gene arrangement among γ-proteobacteria. This gene cluster is not associated with genes involved in the amino acid metabolism of the aspartate family (Lo *et al*., 2009) and our analysis of the *lysC* neighbourhood in *H. elongata* could not find any connections with the ectoine metabolism.

In our opinion, allosteric regulation alone is not a suitable mechanism in regulating LysC activity and thereby the internal ectoine concentration. Feedback or allosteric regulation is used in biological systems to achieve a certain optimal concentration of a metabolite. However, the ectoine content has to be adjusted constantly to match the external osmolarity and it is known that there is essentially a linear relationship between compatible solute content and the salt concentration of the medium (Kuhlmann and Bremer, 2002). Therefore, if an aspartate kinase were involved in controlling ectoine synthesis, then it should be also an osmoregulated enzyme and models explaining the osmoregulatory control of ectoine synthesis have been proposed (Kunte, 2006).

### Ectoine degradation

Ectoine can be accumulated up to molar concentration by *H. elongata* depending on the salinity of the surrounding medium. Furthermore, ectoine can also be utilized as both a carbon and a nitrogen source by *H. elongata* and when ectoine is offered as a nutrient, it still serves as compatible solute (Göller, 1999). In order to find out how ectoine is degraded, the genome of *H. elongata* was compared with *Sinorhizobium meliloti*.

**Fig. 4:**
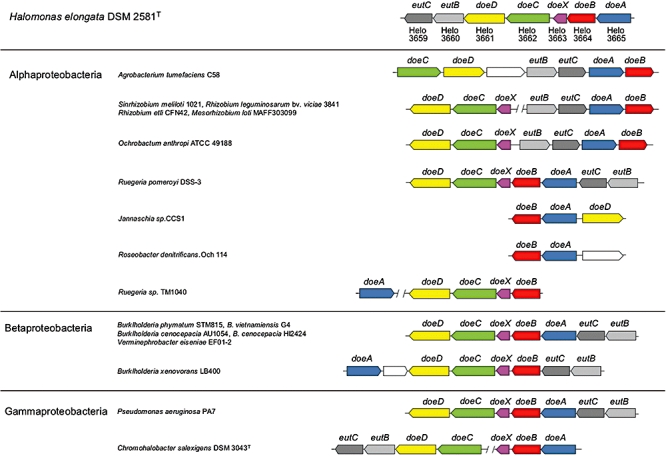
Ectoine-degradation gene organization in *H. elongata* and other bacteria. Ectoine hydrolase genes (*doeA*) are blue, Nα-acetyl-l-2,4-diaminobutyric acid deacetylase genes (*doeB*) are red, genes for AsnC/Lrp-like DNA-binding protein DoeX are pink (*doeX*), aspartate-semialdehyde dehydrogenase genes (*doeC*) are green, l-2,4-diaminobutyric acid transaminase genes (*doeD*) are yellow, *eutB* (putative threonine dehydratase) are light grey, *eutC* (putative ornithine cyclodeaminase) are dark grey. The gene *doeA* and *doeB* are homologues of *eutD* and *eutE* respectively. Only ectoine-degradation genes from bacteria with known genome sequences are depicted. Organisms carrying at least the two genes, *doeA* and *doeB*, could only be found in the proteobacteria domain. The accession numbers of all *doe* genes are listed in Table S6.

In *S. meliloti*, a cluster of five ORFs named *eutABCDE* was described with hypothetical functions in ectoine catabolism (Jebbar *et al*., 2005). No exact function was assigned to any of these five ORFs but their deduced amino acid sequences indicate they are similar to arylmalonate decarboxylases (*eutA*), threonine dehydratases (*eutB*), ornithine cyclodeaminases (*eutC*), aminopeptidases (*eutD*) and glutamate-desuccinylases/aspartoacylases (*eutE*). A homologue for each of the *eutBCDE* genes can be found within the chromosome of *H. elongata* (Fig. 4), but no *eutA* homologue could be identified. The homologues of *eutBC* (*Helo_3660, 3659*) and *eutDE* (*Helo_3665, doeA; Helo_3664, doeB*) are organized in two clusters that are separated by three ORFs (Fig. 4). These three ORFs are homologues of genes annotated as transcriptional regulator (*Helo_3663, doeX*), dehydrogenase (*Helo_3662, doeC*) and transaminase (*Helo_3661, doeD*) respectively.

The ORFs *Helo_3665*, *Helo_3664*, *Helo_3662* and *Helo_3661* were chosen for mutation experiments, as the predicted enzymatic function of their gene products would make them candidates for the breakdown of ectoine into aspartate (Fig. 1). All four ORFs were deleted (in-frame null mutation) and the resulting mutants were either unable to utilize ectoine as a carbon source or they displayed reduced growth on ectoine. We therefore named the cluster *doeABCD* (**d**egradation **o**f **e**ctoine ABCD, Fig. 4). Based on experiments (described below) and sequence homology, we named these ORFs *doeA* (*Helo_3665*, ectoine hydrolase), *doeB* (*Helo_3664*, Nα-acetyl-l-2,4-diaminobutyric acid deacetylase), *doeC* (*Helo_3662*, aspartate-semialdehyde dehydrogenase) and *doeD* (*Helo_3661*, diaminobutyric acid transaminase). A fifth ORF belonging to the *doe* cluster is located between *doeB* and *doeC*, and is named *doeX* (*Helo_3663*, Fig. 4). Deletion of *Helo_3660* (*eutB*) and *Helo_3659* (*eutC*) did not impair growth of the corresponding mutants on ectoine and we infer from these results that *eutBC* does not participate in ectoine degradation.

From the mutational and additional analytical experiments we concluded that the degradation of ectoine proceeds via hydrolysis of ectoine (DoeA) to the novel compound Nα-acetyl-l-2,4-diaminobutyric acid, deacetylation of Nα-acetyl-l-2,4-diaminobutyric acid (DoeB) to l-2,4-diaminobutyric acid, and a transaminase reaction (DoeD) leading to aspartate-semialdehyde. Finally, aspartate-semialdehyde is oxidized by DoeC to aspartate (Fig. 1). The proposed pathway is based on the following experimental and computational data:

(i)The *doeA* gene (*Helo_3665*) is a homologue to *eutD* and codes for a 399 aa protein (44.9 kDa, pI 5.0) that belongs to the peptidase-M24 family. Within that family, DoeA is similar to creatinase (creatine amidinohydrolase), which catalyses the hydrolysis of creatine to sarcosine and urea (Coll *et al*., 1990). Deletion of *doeA* created a mutant KB41 that was unable to grow on ectoine as carbon source. The *doeA*^+^ wild type could be restored in the Δ*doeA* mutant by expressing *doeA in trans* from plasmid pKSB7 (pJB3Cm6::*doeA*), proving that no polar effect was causing the defect in ectoine catabolism.

**Fig. 5:**
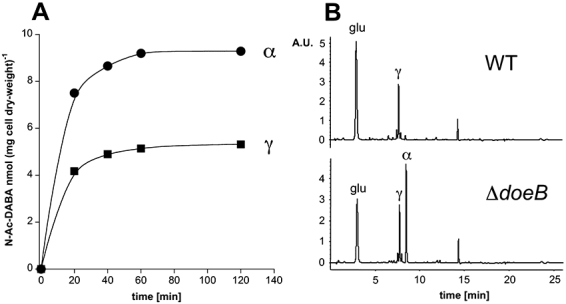
Chromatographic analysis of the cytoplasm from (A) *E. coli* expressing recombinant *doeA* and (B) *H. elongata* wild type and Δ*doeB*-mutant strain KB42.A. Time course of Nα-Ac-DABA and Nγ-Ac-DABA formation from ectoine in *E. coli* expressing recombinant *doeA*. Cells were grown in mineral salt medium containing 340 mM NaCl. Ectoine was added (1 mM) and the cytoplasmic Nα-Ac-DABA (α) and Nγ-Ac-DABA (γ) content was determined by OPA-HPLC shortly before and 20, 40, 60 and 120 min after ectoine was added. Data presented are the mean from two independent experiments. No N-Ac-DABA was formed in *E. coli* cells without *doeA*.B. Chromatogram of the cytoplasm from *H. elongata* KB42 (Δ*doeB*) and wild-type cells (WT), which were grown on mineral salt medium (1.03 M NaCl) in the presence of 500 µM glucose and 10 mM ectoine. After depletion of carbon-source glucose, the amino acid content of both strains was determined by OPA-HPLC. In wild-type cells glutamate (glu) and the ectoine precursor Nγ-Ac-DABA (γ) are detectable. In Δ*doeB* mutant strain KB42 Nα-Ac-DABA (α) is accumulated as the predominant amino-reactive solute. Similar results were obtained when ectoine degradation was induced by hypoosmotic shock diluting the medium from 1.03 M down to 0.51 M NaCl. A.U., arbitrary units.

To gain information on the enzymatic reaction catalysed by DoeA, the *doeA* gene was expressed in *E. coli* BL21 cells from plasmid pKSB11. After 16 h of expression, 1 mM ectoine was added to the salt medium (340 mM NaCl). *E. coli* is known to accumulate ectoine in the cytoplasm as compatible solute via osmoregulated transporters ProU and ProP but is unable to metabolize ectoine (Jebbar *et al*., 1992; Racher *et al*., 1999). The cytoplasmic fraction of *E. coli* was analysed by HPLC allowing for the detection of N-acetyl-l-2,4-diaminobutyric acid (N-Ac-DABA), the product of ectoine hydrolysis. Two forms of N-Ac-DABA were detected, which could be distinguished by comparison with a standard of Nγ-acetyl-l-2,4-diaminobutyric acid (Nγ-Ac-DABA) and Nα-acetyl-l-2,4-diaminobutyric acid (Nα-Ac-DABA). While neither Nα-Ac-DABA nor Nγ-Ac-DABA was formed in *E. coli* without *doeA*, both forms could be detected at a ratio of 2:1 in cells carrying *doeA* (Fig. 5). This unambiguously demonstrates that DoeA functions as ectoine hydrolase.

Similar results concerning ectoine hydolysis were obtained with *H. elongata*Δ*ectA*-mutant KB1 after growth with ectoine as carbon source. Although EctA-catalysed acetylation of DABA leading to Nγ-Ac-DABA is blocked, Nγ-Ac-DABA is accumulated in roughly the same concentration as in wild-type cells. In addition, Nα-Ac-DABA is also present although the pathway towards aspartate remained genetically unchanged. Apparently, ectoine hydrolase activity leads to the formation of Nα-Ac-DABA and Nγ-Ac-DABA also in the *H. elongata* background. Any significant contribution of ectoine synthase EctC to Nγ-Ac-DABA formation is rather unlikely, as purified EctC from *H. elongata* is described as an enzyme with no detectable reverse activity (Ono *et al*., 1999).

The specificity of ectoine hydrolase DoeA with respect to the isoforms of N-Ac-DABA remains somewhat ambiguous. While the results on DoeA expression in *E. coli* (Fig. 5) suggest the formation of both, Nα-Ac-DABA and Nγ-Ac-DABA, upon ectoine hydrolysis (as depicted in Fig. 1), cleavage of ectoine by DoeA could also produce only one isomer, which subsequently has to be converted into the corresponding isomer by an acetyltransferase. Whatever mechanism is employed by the cell, both are suitable and allow for the formation of Nα-Ac-DABA, which is the essential substrate for the subsequent catabolic enzyme DoeB (see below).

(ii)The 342 aa protein (36.6 kDa, pI 4.6) encoded by *doeB* (*Helo_3664*, homologue to *eutE*) is closely related to proteins of the succinyl-glutamate desuccinylase/aspartoacylase subfamily, which are part of the M14 family of metallocarboxypeptidases (Makarova and Grishin, 1999). The desuccinylase is involved in arginine catabolism while the aspartoacylase cleaves N-acetyl-aspartate into aspartate and acetate (Le Coq *et al*., 2008). Deletion of *doeB* resulted in a mutant KB42 that could not utilize ectoine as carbon and nitrogen source. The *doeB*^+^ wild type could be restored in mutant KB42 (Δ*doeB*) by expressing *doeB* from plasmid pJSB3 (pJB3Cm6::*doeB*). Analysing the cytoplasmic fraction of wild type and Δ*doeB* strain by HPLC revealed that Nα-acetyl-l-2,4-diaminobutyric acid is accumulated as the predominant amino-reactive solute in mutant KB42 while no Nα-Ac-DABA is detectable in the *H. elongata* wild type (Fig. 5). The Nγ-Ac-DABA concentration, however, remains still the same in KB42 and wild-type cells. Based on the results from the feeding experiments (no growth on ectoine as nitrogen-source) and the HPLC analysis (accumulation of Nα-Ac-DABA, unchanged Nγ-Ac-DABA level), we propose that the hydrolysis of ectoine is directly succeeded by DoeB-catalysed deacetylation of Nα-Ac-DABA.

While Nγ-Ac-DABA is not a substrate for DoeB, it serves again as a substrate for ectoine synthase EctC and can be converted back to ectoine. Nα-Ac-DABA, however, is removed from this cycle by deacetylation to DABA, which then can either flow off to aspartate or re-enter the ectoine synthesis pathway in wild-type cells. This closes the cycle of synthesis and degradation, which is powered by acetylation and deacetylation of DABA and Nα-Ac-DABA respectively (Fig. 1). As we will describe below, such a cycle provides a fast mechanism for the cell to regulate the cytoplasmic ectoine concentration.

(iii)The *doeD* gene (*Helo_3661*) encodes a putative aspartate aminotransferase (469 aa, 50.8 kDa, pI 5.6) with similarities to the PLP-dependent aspartate aminotransferase superfamily. Deletion of *doeD* resulted in mutant KB48 (Δ*doeD*) that was impaired in growth on ectoine as a sole carbon source. In saline minimal medium (510 mM NaCl), the *doeD* deletion reduced the growth rate of strain KB48 threefold down to 0.077 h^−1^ compared with 0.248 h^−1^ observed with the wild type indicating a participation of DoeD in ectoine degradation. Deletion of the aminotransferase gene *ectB*, resulting in mutant SB1 (Δ*ectB*), or in *doeD*-mutant KB48, resulting in the double knockout mutant SB1.1 (Δ*ectB*, Δ*doeD*), did not further reduce growth on ectoine. Because both mutant strains were still able to synthesize ectoine other transaminases (or amidases) are still active in *H. elongata*, converting DABA to L-aspartate-β-semialdehyde, and likewise, L-aspartate-β-semialdehyde to DABA, which can be readily explained by the rather broad substrate specificity of transaminases (Fotheringham, 2000).

(iv)The *doeC* gene (*Helo_3662*), located upstream of *doeD*, encodes a putative dehydrogenase (493 aa, 53.1 kDa, pI 4.8) and is most closely related to those dehydrogenases that act on aldehyde substrates. Knockout of *doeC* abolished growth of *H. elongata* strain KB47 (Δ*doeC*) on medium containing ectoine as sole carbon source. Growth could be restored in *doeC*-mutant KB47 when ectoine was offered as nitrogen source in the presence of 10 mM glucose. This finding, together with the results obtained from the *doeB* mutant KB42, helped to determine the sequential order of the enzymatic reactions as depicted in Fig. 1, in which the dehydrogenase reaction and the transaminase reaction follow after the DoeB catalysed deacetylation of Nα-Ac-DABA.

However, according to the proposed pathway, acetate is split off from Nα-Ac-DABA in the second reaction. Acetate released in this reaction could serve as carbon source and should in principle allow dehydrogenase mutant KB47 to display at least some minimal growth on medium containing ectoine. We were able to prove through feeding experiments, that *H. elongata* can indeed utilize acetate as carbon source and that wild-type strain DSM 2581^T^, strain KB41 (Δ*doeA*) and strain KB47 (Δ*doeC*) are able to grow on medium containing 10 mM, 20 mM and 40 mM acetate respectively (Fig. S3). Adding 10 mM ectoine to the acetate medium suppressed growth of dehydrogenase-mutant KB47, while strain KB41 and KB42, as well as wild-type DSM 2581^T^, still managed to grow (Fig. S3). This finding explains why strain KB47 cannot grow on ectoine medium and fails to metabolize the internal acetate, which is split off from Nα-Ac-DABA during ectoine degradation. For now, we can only speculate about the underlying mechanism that causes the inability of strain KB47 to utilize acetate in the presence of ectoine. Internal accumulation of intermediates such as DABA or aspartate-semialdehyde to toxic levels cannot be ruled out as the reason for growth inhibition. However, because mutant KB47 can still feed on ectoine as nitrogen donor in the presence of 10 mM glucose, we favour the idea that some kind of catabolite repression is the reason for the failure of KB47 to grow on medium containing both acetate and ectoine.

### Transcriptional organization of *doeABX* and DNA-binding protein DoeX

To characterize the genetic organization of the *doe* components, we carried out RT-PCR and RACE-PCR experiments. RT-PCR revealed that *doeAB* and the adjacent ORF, which was named *doeX* (*Helo_3663*), are organized as one operon (Fig. S4). The *doeCD* components are not part of the *doeABX* operon. The transcriptional initiation site of the *doe*-locus was mapped by RACE-PCR experiments and could be pinpointed 154 nt upstream of *doeA*. Inspection of the sequences upstream of the transcription start site revealed the presence of putative −10 and −35 sequences that resemble the consensus sequences of σ^70^-dependent promoters (Fig. S4).

**Fig. 6:**
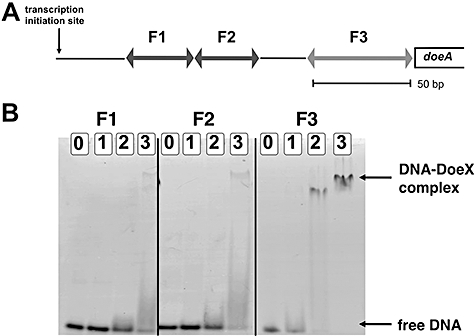
Electrophoretic mobility shift assays (EMSA) with DNA-binding protein DoeX.A. EMSA were performed to detect interactions of purified DoeX protein with labelled DNA fragments F1, F2 and F3 located upstream of the *doeABX* operon.B. In the presence of competitor DNA (500-fold to 1000-fold excess), DoeX specifically binds to fragment F3 resulting in a mobility shift on a non-denaturing polyacrylamide gel. Each assay (20 µl) contained 2 pmol labelled DNA. Lane 0, assay without DoeX protein (control); lane 1, 10 pmol DoeX; lane 2, 20 pmol DoeX; lane 3, 40 pmol DoeX.

The *doeX* gene was annotated as an ORF encoding a 158 aa transcriptional regulator (17.9 kDa, pI 5.8) belonging to the AsnC/Lrp family of DNA-binding proteins. Proteins of the AsnC/Lrp family are known to control the expression of a large number of operons, often in response to amino acid effectors, and can act as both transcriptional repressor and activator (Thaw *et al*., 2006). To test whether DoeX is a DNA-binding protein, recombinant DoeX protein was purified from *E. coli* and applied to electrophoretic mobility shift assays with labelled DNA (Fig. 6). DoeX was found to specifically bind to a 46 nt sequence located directly upstream to the *doeA* start codon.

### Organization of *doe* genes in bacteria

**Fig. 7:**
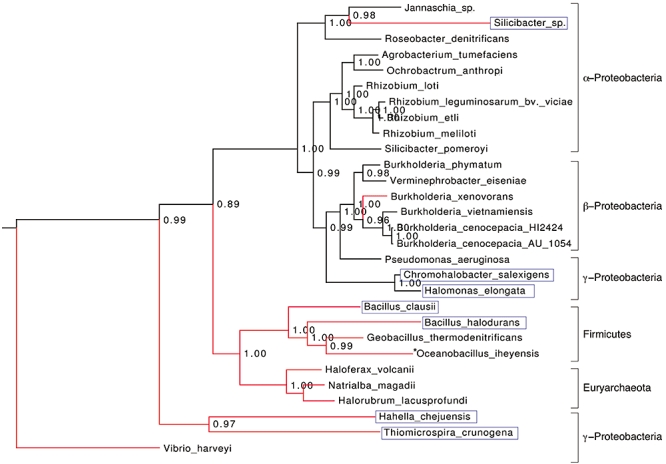
Phylogenetic tree of DoeA homologues and their position in the standard phylogeny (‘tree of life’). Branches marked in black correspond to genomes with the same ‘clustered’ organization of the *doeAB* genes. Red colour is used for those genomes where *doeA* is located separate from *doeB* <*Ruegeria*[previously *Silicibacter* (Yi *et al*., 2007)] sp. TM1040, *B. xenovorans*> or where *doeB* is not present at all. Organisms considered to be ectoine synthesizers based on the presence of the *ectABC* genes are boxed in blue. Numbers printed at the nodes are confidence values (varying from 0 to 1) derived from Bayesian statistics. *Oceanobacillus iheyensis carries an *ectBC* cluster but no *ectA*.

From comparative genomic data, the ectoine degradation pathway described for *H. elongata* is mostly employed by non-halophilic organisms that, according to their genetic makeup, are unable to synthesize ectoine *de novo*. The *doeA/eutD* and *doeB/eutE* sites could be found within the genomes of 18 bacteria, all belonging to the proteobacteria (Fig. 4). Besides *H. elongata*, only two of them are ectoine-synthesizing organisms, namely *C. salexigens* and *Ruegeria* (*Silicibacter*) sp. TM1040. Homologues of both the *doeA* and *doeB* sites of *H. elongata* can be found in many other *Bacteria*, predominantly in the Rhizobiales of the α-proteobacterial domain and in the Burkholderiales of the β-proteobacteria. For further analysis, we selected only a few representatives from larger sets of highly similar sequences originating from species such as *Burkholderia*, which appear overrepresented in the list of completely sequenced genomes. The reduced set of sequences contains 18 members, which share a relatively high similarity (48% to > 80% sequence identity) with *doeA* of *H. elongata* (*Helo_3665*). With two exceptions (*Ruegeria* sp. TM1040 and *Burkholderia xenovorans* LB400), *doeAB* is clustered in all these genomes. A second set of nine *doeA* homologues with a lower similarity to *Helo_3665* (30–45% sequence identity) was found within the Firmicutes, Euryarchaeota and the γ-proteobacteria groups. In these genomes, the *doeA* site is located in a different genetic neighbourhood with no other genes of the ectoine degradation pathway being present. This is illustrated in the computed *doeA* phylogeny (Fig. 7), where the two sequence sets form separate branches (drawn in black and red colour respectively). The two organisms in which *doeA* and *doeB* are not adjacent (*Ruegeria* sp. TM1040 and *B. xenovorans*) are highlighted (branches also drawn in red). For reference, organisms carrying the *ectABC* genes are marked in blue.

In most ectoine producers compared in our investigation (Fig. 2), either only *doeA*-like ORFs could be found, or none of the *doe* components were present. We therefore assume that either ectoine is not metabolized in these bacteria or that alternative pathways in ectoine degradation must exist with, and without, DoeA participation. In analogy to the glutamate-ornithine pathway, ectoine could be alternatively metabolized to aspartate via Nα-Ac-DABA, Nα-acetyl-aspartate-semialdehyde and Nα-acetyl-aspartate. Also, it cannot be ruled out that in organisms without Doe enzymes, a reverse synthesis pathway degrades ectoine, bypassing the irreversible EctA acetyltransferase reaction.

### Ectoine metabolism: reconstruction, modelling and energetic considerations

The metabolic capabilities of an organism are one of the major aspects of cellular physiology. Genome annotation with an emphasis on metabolic reconstruction provides a basis to analyse metabolic capabilities and their impact on the specific adaptation of the organism to its natural environment. The immense amount of data generated, such as the 1265 complete or partial EC numbers assigned for *H. elongata*, calls for a computational strategy to drive a comprehensive analysis. As a first step, we have concentrated on the metabolism of ectoine, as this compatible solute is a key metabolite for survival and success of the halophilic bacterium *H. elongata*. A Flux Balance Analysis (Varma and Palsson, 1993a,b;) approach was selected to create a mathematical model of ectoine metabolism as detailed in *Supporting information*. Using this model, we have analysed two aspects of ectoine metabolism: (i) The energy balance of the glucose to ectoine conversion computed and compared with previous calculations reported in the literature (Oren, 1999). (ii) We attempted to predict the role of the proposed ectoine biosynthesis/degradation cycle.

**Fig. 8:**
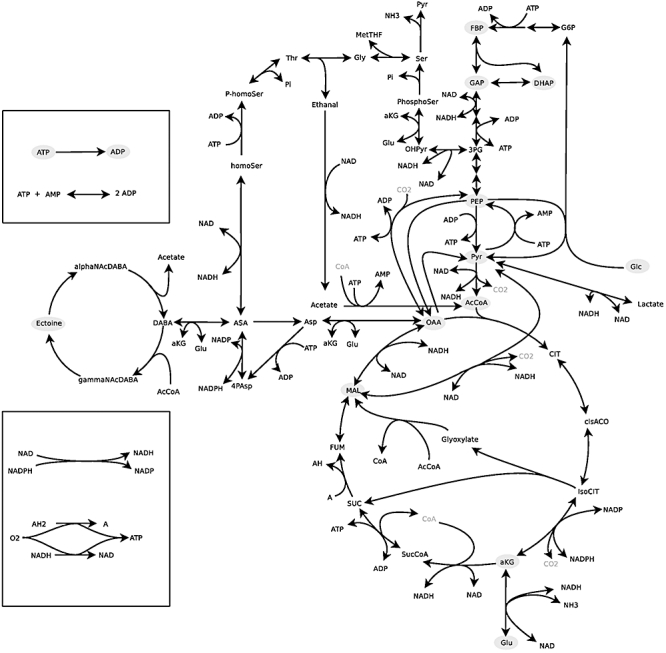
Depiction of the pathways considered in the metabolic model for ectoine.

The metabolic network, reconstructed from the genome of *H. elongata*, contains a series of enzymes, which are involved in biosynthesis of ectoine from glucose (Fig. 8). Overall, glucose is converted to two molecules of PEP/pyruvate, one of which is converted to acetyl-CoA, the other to aspartate-semialdehyde via oxaloacetate. Ectoine is then produced from one molecule each of aspartate-semialdehyde and acetyl-CoA. The set of available enzymes allows for several distinct pathways, some of which result in an identical overall reaction while others differ with respect to the ATP balance. An exhaustive analysis is required to ensure that all alternatives have been considered. While this is difficult by manual inspection, this becomes a feasible task once a model is available so that mathematically sound techniques can be applied.

In one of the possible pathways, PEP is converted to oxaloacetate through PEP carboxylase (EC 4.1.1.31). This particular case has been used for the previous calculations on the energetic costs of ectoine synthesis presented by Oren (1999). Oren's calculations assume nitrogen assimilation via glutamine synthetase (EC 1.4.1.13). With this assumption, the conversion of glucose to ectoine costs 2 ATP. However, an ATP-neutral pathway is possible using glutamate dehydrogenase (EC 1.4.1.2) as a more energy-efficient nitrogen assimilation reaction. This is based on the annotation of a NADH-dependent glutamate dehydrogenase (Helo_3049) in *H. elongata*. An ATP-neutral conversion of glucose to ectoine is consistent with a calorimetric analysis carried out by Maskow and Babel (2001), in which the authors conclude that an efficiency of approximately 100% was reached in the experiment.

The annotated genome allows for yet another solution, in which a total conversion of glucose into ectoine is possible with the simultaneous generation of ATP. The additional ATP can be obtained by using a different pathway, which proceeds via pyruvate to oxaloacetate and is catalysed by the malic enzyme (EC 1.1.1.38) and malate dehydrogenase (EC 1.1.1.37). An estimation of the reaction enthalpy for the conversion of glucose to ectoine shows that this pathway is not only stoichiometrically but is also thermodynamically feasible (see *Supporting information*). Currently, this hypothetical pathway is awaiting an experimental validation.

In summary, three alternative possibilities for conversion of glucose to ectoine differ in ATP production: (i) the pathway originally proposed by Oren (1999) requires two ATP for each molecule of ectoine, (ii) the Oren pathway with a modified nitrogen assimilation step is energy-neutral and (iii) the most energy-efficient pathway, which is thermodynamically feasible and has been revealed by modelling of ectoine biosynthesis, allows to generate one ATP per ectoine molecule. This has clear physiological implications. In case (ii), the energy-neutral pathway allows for a 100% ectoine yield only if no other ATP-utilizing process operates. This does not reflect the reality of a living cell, where maintenance processes require additional ATP generation. Only pathway (iii), which is the most energy-efficient, allows a 100% ectoine yield as long as other metabolic processes do not drain more than one ATP per glucose.

The novel degradation pathway of ectoine we introduced here may result in an apparent futile cycle, in which ectoine can be synthesized and degraded simultaneously resulting in a net conversion of acetyl-CoA into acetate. Acetate can be reconverted to acetyl CoA by acetate : CoA ligase (AMP-forming) (EC 6.2.1.1). Therefore, if such a cycle were active, it would result in an increased apparent cost of two ATPs per turn for ectoine synthesis.

The question remains why the cell would invest two high-energy phosphate bonds to run a cycle of ectoine synthesis and degradation. We believe that such a cycle is an elegant mechanism to control the speed of change in internal ectoine concentration as response to external changes in osmolarity. The turnover (or response) time of a metabolite, defined as the ratio between its concentration and the flux through it in the steady state, has been identified as a good indicator of the timescale of its transient responses (Nikerel *et al*., 2009). Metabolites with a high turnover tend to complete transitions faster than those with a low turnover. Thus, by keeping a flux through the synthesis/degradation cycle, the cell can achieve fast changes in ectoine levels to quickly respond to changes in external osmolality.

According to our model of a simultaneous activity of ectoine synthesis and degradation (ectoine cycle), disrupting the ectoine degradation pathway should lead to a lower ATP load for cells synthesizing ectoine and thereby result in higher ectoine productivity. In order to verify our proposed ectoine cycle and the mathematical model for ectoine synthesis, the *doeA* gene was deleted in the ectoine excreting mutant KB2.11 (Δ*teaABC*). Mutants with a dysfunctional or missing TeaABC ectoine transporter were shown to lose ectoine constantly to the medium (Grammann *et al*., 2002; Kunte *et al*., 2002). As the mathematical model was developed for non-growing cells, ectoine synthesis and export was analysed in minimal medium (510 mM NaCl) with non-growing cells (OD_600_ = 2.6; 100 µg ml^−1^ chloramphenicol) of mutant KB2.13 (Δ*teaABC*, Δ*doeA*) and the parental strain KB2.11. While KB2.11 accumulated 40 mg ectoine l^−1^ h^−1^, the degradation mutant KB2.13 accumulated 50 mg ectoine l^−1^ h^−1^, which corresponds to a 20% higher productivity of strain KB2.13. This result supports our proposed regulatory ectoine cycle and demonstrates the usefulness of the mathematical model in predicting the effect of mutations on the bacterial metabolism.

### Conclusion and outlook

Determining the complete genome sequence of *H. elongata* DSM 2581^T^ has increased our understanding of the metabolism of ectoine and will be the first step in our work towards optimizing the industrial production of the compatible solute ectoine. We were able to completely reconstruct ectoine biosynthesis from glucose and ammonia, by annotating the genes encoding for the central metabolic enzymes and the pathways leading to the ectoine precursors aspartate and glutamate, including the enzymes responsible for the metabolite interconversions at the PEP-pyruvate-oxaloacetate node. In addition, we have identified a pathway for ectoine degradation and shown its cyclic connection to ectoine synthesis. On the basis of these data, a Flux Balance Analysis model of ectoine metabolism has been developed.

Initial steps in using this new data for strain optimization have been undertaken and are described briefly in this paragraph. First, the deletion of gene *doeA*, encoding the ectoine hydrolase protein, which catalyses the first step in the ectoine degradation pathway, has led to increased volumetric productivity of ectoine as predicted by our metabolic model. Furthermore, the genome sequence has enabled us to identify the gene for polyhydroxyalkanoate synthase *phaC*, which is important because *Halomonas* species are also known to synthesize polyhydroxyalkanoates in parallel to ectoine (Mothes *et al*., 2008). Finally, mutagenesis experiments are now underway to abolish polyhydroxyalkanoate biosynthesis by deleting *phaC*, which will hopefully enable new insights into metabolism of this energy storage compound and may also lead to a mutant strain that is able to convert glucose to ectoine more efficiently.

The metabolic model presented in this study only considers ectoine biosynthesis and the influence of different ATP-loads on its synthesis (Fig. S5). We are currently developing an extended model that will also include growth (increase of biomass). The extended model will help us to evaluate the effect of alternative C-sources on ectoine production, including biologically and environmentally relevant substances such as glutamate or glycerol. In addition, the use of extended models allows us to make specific changes in the metabolism of the organism, resulting in an increase of the carbon flux towards the formation of ectoine. Koffas and co-workers (Koffas *et al*., 2003) successfully used this strategy to improve lysine production in *C. glutamicum*. By coordinated overexpression of genes encoding two flux-controlling enzymes in central carbon metabolism and the lysine pathway, they were able to enhance the carbon flux towards its product lysine, resulting in an increase in lysine specific productivity by 250%.

The availability of genomic information paves the way for post-genome technologies such as DNA array and proteomics, which can now be employed with *H. elongata*. These technologies will accelerate the studies of osmoregulation in this halophilic bacterium and will enable us to manipulate its metabolism to create more efficient producer strains for ectoine.

## Experimental procedures

### Sequencing strategy

Whole genome sequencing was carried out in a two-step process. First, *de novo* sequencing was done using a Roche/454 GS 20 instrument (Margulies *et al*., 2005). Using the manufacturer's protocols random genomic DNA libraries were constructed and two sequencing runs were carried out with GS 20 chemistry (max. read length 100 bp). A total of 722 276 reads resulting in 70.6 Mb (average read length 98 bp) were assembled into 100 contigs of 4.0 Mb sequence using the Newbler assembler (Margulies *et al*., 2005). The resulting average coverage was 17.4 fold. As the applied assembly parameters were highly conservative, multicopy segments in the genome caused frequent termination of contig assembly in order to avoid misassemblies. Phred-like quality values were computed for the Newbler-generated contig sequences, similar to Sanger-based quality scores. The resulting contigs were then treated as if they were individual long sequencing reads in later phases of the assembly. In the second phase, a fosmid library was generated and end-sequenced. Phosmid end-sequences were co-assembled together with the 454-based contigs using the Phred-Phrap-Consed program package (Gordon *et al*., 1998). Fosmid end-sequences were used to order contigs. Remaining gaps were closed by either fosmid sequencing or by a PCR approach. The rRNA operons and other long repeats were solved by a mini-assembly strategy (Pfeiffer *et al*., 2008a). Each of the four rRNA operons was amplified as a long PCR product and sequenced on both strains by a nested set of primers. The individual rRNA operons were assembled and the resulting contigs were exported (including quality values) and added to the final assembly as if they were single reads.

### Genome annotation

For gene prediction, the Reganor program (McHardy *et al*., 2004) from the annotation package GENDB (Meyer *et al*., 2003) was used, which integrates results from Critica (Badger and Olsen, 1999) and Glimmer (Delcher *et al*., 1999). Genes for transfer RNA (tRNAs) were predicted using tRNAscan (Lowe and Eddy, 1997). Genes encoding ribosomal RNA (rRNAs) were predicted using RNAmmer (Lagesen *et al*., 2007) except for the 16S rRNA 3′ end, which was set according to Lin and co-workers (Lin *et al*., 2008). Automated function prediction was performed using the Metanor tool of the GenDB genome annotation system (Meyer *et al*., 2003). For enzymes, these were compared with the data obtained by the PRIAM program (Claudel-Renard *et al*., 2003) in a manual curation effort. We used the HaloLex system (http://www.halolex.mpg.de) (Pfeiffer *et al*., 2008b) as a genome annotation platform.

### Genome analysis

The protein set from *H. elongata* was compared with that of *C. salexigens* and other organisms using orthologous group analysis (COGs). Assignments for *C. salexigens* and other species were taken from eggNOG 2.0 (Muller *et al*., 2010). Assignments for *H. elongata* were made by a PERL script based on BlastP comparisons with the eggNOG 2.0 protein sequences. The same COG has been assigned for 98% of the 2367 proteins with a bidirectional best blast between *H. elongata* and *C. salexigens*. Assignment discrepancies are concentrated in distant bidirectional best blast pairs with reduced sequence identity values. For further details see the *Supporting information*.

We attempted to identify COGs, which are preferentially occupied in halophilic/marine organisms. We compared the occupancy between 3 halophilic/10 marine bacteria versus 14 standard bacteria (listed in Table S1). COGs were considered halophilic when the fraction of organisms having at least one COG member was twice that of the standard organisms. As an additional criterion, the COG had to be occupied in 2 of 3 halophiles or 5 of 13 halophile/marine organisms.

High-salt adaptation may result in protein adaptation, e.g. by adjusting protein pI values. We used the COG assignments to select protein pairs between *H. elongata* and those query organisms selected for identification of halophile-specific COGs. If there is only one COG member in the two organisms, the protein pair is used for subsequent computation of the difference in pI value. With this selection method, the pI difference could be averaged over *c*. 500 and 950 protein pairs for each of the 27 organisms included in the analysis (Table S2, Table S4).

### Data analysis and comparative genomics

To find all genes encoding proteins involved in ectoine degradation and to compare gene clusters of ORFs encoding Doe-like proteins, BLAST queries (Altschul *et al*., 1997) of all available microbial genomes included at NCBI (http://ftp://ftp.ncbi.nih.gov/genomes/Bacteria) were conducted. Specifically, for the genomic *doeA* comparison, we identified homologues of the *Helo_3665* amino acid sequence by performing a standard protein BLAST search (Altschul *et al*., 1997) in these databases. Out of all BLAST targets (with at least 30% sequence identity to *Helo_3665*) we compiled a selection of 29 sequences (including *Helo_3665*) by selecting only a few representative strains of species, which appear overrepresented in the list of completely sequenced genomes (e.g. *Burkholderia*). A peptidase sequence of *Vibrio harveyi* (20% sequence identity) was added and used as an out-group for phylogenetic analysis. For this set of 30 amino acid sequences a multiple alignment was computed using MUSCLE (Edgar, 2004). Poorly aligned and gapped positions were eliminated from the original MUSCLE alignment using the GBlocks algorithm (Castresana, 2000), which resulted in an alignment with 271 (54% of the original 497) positions. Finally, MrBayes (Huelsenbeck *et al*., 2001; Altekar *et al*., 2004) was employed for constructing the phylogenetic tree with the clade credibility values being based on Bayesian statistics. The final tree was drawn and rooted using the ATV/Archaeopteryx tool (Zmasek and Eddy, 2001). Using alternative strategies and software for alignment [e.g. employing T-Coffee (Notredame *et al*., 2000), ClustalW (Chenna *et al*., 2003), with and without GBlocks polishing] and phylogenetic analysis [e.g. employing PhyML (Guindon *et al*., 2005), PHYLIP (Felsenstein, 1989)] yielded qualitatively very similar results. The complete analysis pipelines were conducted with the help of the MIGenAS toolkit (Rampp *et al*., 2006).

### Mathematical modelling

The mathematical modelling was carried out as outlined in ‘Network Reconstruction and Flux Balance Analysis’ and further detailed in *Supporting information*. All simulations were performed using Scilab 4.1.2 (INRIA ENPC). Degenerate solutions were explored using Flux Variability Analysis.

### Bacterial strains, plasmids and growth conditions

Bacterial strains, vectors and recombinant plasmids used for this study are listed in Table S5. *H. elongata* strains were grown aerobically at 30°C on MM63 medium (Larsen *et al*., 1987) with glucose as carbon source. For certain experiments, ectoine was used as a carbon and nitrogen source, respectively, as specified. NaCl was added to concentrations as indicated. *E. coli* strains were grown aerobically at 37°C in Luria Bertani medium or MM63 minimal medium. Antibiotics with *E. coli* were used at the following concentrations: kanamycin 50 µg ml^−1^, ampicillin 100 µg ml^−1^, chloramphenicol 30 µg ml^−1^.

### DNA isolation and manipulation

Total DNA from *H. elongata* was isolated according to a modified procedure of Marmur (Marmur, 1961). Routine manipulation of DNA, plasmid isolation, construction of recombinant plasmids, electrophoresis of DNA on agarose gels, and transformation were carried out according to standard procedures (Sambrook and Russell, 2001).

### Purification of DoeX protein

Gene *doeX* was ligated into plasmid pET101 (Invitrogen), which allows the expression of recombinant protein with a C-terminal sequence of six consecutive histidine-residues (His-Tag) for subsequent purification by Ni-chelation chromatography. The resulting plasmid pKSB15 was transferred into *E. coli* BL21 (Invitrogen) and *doeX* expression was induced by the addition of IPTG (1 mM) to the medium of exponentially growing cells (OD_540_ 1.0). After 5 h of induction, cells were harvested by centrifugation and disrupted using the BugBuster Reagent from Novagen (Darmstadt) according to the manufacturer's instruction. The soluble cell extract was loaded onto columns with Ni^2+^-resin (His-bind Resin, Novagen) and after washing, DoeX protein was released from the resin applying 1 M imidazole buffer. DoeX purification was monitored by SDS-PAGE and protein concentration was determined by applying the Bradford assay.

### Generation of deletion mutants

DNA sequences upstream and downstream from the desired gene were joined together by applying the splicing by overlap extension PCR technique (Horton *et al*., 1989). The resulting PCR fragments were ligated into the shuttle vector pK18*mobsacB* (Schäfer *et al*., 1994) and transferred into *H. elongata* by *E. coli* S17-1 mediated conjugation (Simon *et al*., 1983; Kunte and Galinski, 1995). Deletion mutants, arising after double cross-over, were then selected for on LBG medium containing 22% (w/v) sucrose at 37°C. The deletion sites were verified by PCR and DNA-sequencing techniques.

### Complementation experiments

To complement the mutations in strains KB41 (Δ*doeA*) and KB42 (Δ*doeB*), ORFs *doeA* and *doeB*, respectively, were expressed *in trans* with the help of shuttle vector pJB3Cm6 (Blatny *et al*., 1997). PCR-amplified *doeA* and *doeB*, respectively, were inserted into HindIII/XbaI-cut pJB3Cm6. The resulting recombinant plasmids pKSB7 (pJB3Cm6::*doeA*) and pJSB3 (pJB3Cm6::*doeB*) were transferred into the corresponding mutants via conjugation as described previously (Simon *et al*., 1983; Kunte and Galinski, 1995). *H. elongata* cells carrying the recombinant plasmid were selected on minimal medium containing chloramphenicol (30 µg ml^−1^) and tested for growth with ectoine as carbon source on mineral salt medium (680 mM NaCl).

### RT-PCR and RACE-PCR

Total RNA was isolated from exponentially growing cells using a modified hot phenol method (Sambrook and Russell, 2001) and further purified using the NucleoSpin RNA-isolation kit (Macherey & Nagel) according to the manufacturer's instruction. cDNA for *doeABX* operon analysis was synthesized using the reverse primers (5′-TCGAACTTGACCAGGTAATCC-3′) binding in *doeX* and (5′-GGCACCGTACTCGACCTCAC-3′) binding to *doeC*. The transcriptional initiation sites were mapped by a modified RACE-PCR procedure based on a protocol by Gerhart and co-workers (Gerhart *et al*., 2005) using RNA from exponentially growing cells adapted to 680 mM NaCl. Transcription initiations site of *doeA* was mapped with reverse primer (5′-ACCAGTTGACCAGCGAGTTG-3′), *ectA* with reverse primer (5′-CGCTGATAGTGGTCTCG-3′), *ectC* with reverse primer (5′-TTCGCGGTGCACTTCGTT-3′), and a forward primer binding to the artificial RNA-adaptor attached to the 5′-end of the mRNAs. The transcription initiation site is defined by the boundary between the artificially attached RNA and the 5′-end of the transcript.

### Electrophoretic mobility shift assay

Oligonucleotides used for this assay were fragment F1 (5′-TGTTAACAAATGTCATGACAATGAACAT-3′), F2 (5′-GGCCCTGACACGGTCGGCAAGTTAGCGC-3′), and F3 (5′-AGAGGCAGCCGGATATCGGTGACATGATCGTTTGGCGAGCGATTTC-3′). Double-stranded oligonucleotides of F1, F2 and F3 were generated by heating the complementary oligonucleotides in TA-buffer (330 mM TrisAc pH 7.8, 660 mM KAc, 100 mM MgAc, 5 mM DTT) for 10 min at 95°C and slowly cooling at room temperature. Double-stranded nucleotides were labelled by Fluorescein. Binding of DoeX to DNA was carried out in 20 µl SP1 buffer (4% glycerol, 1 mM MgCl_2_, 0,5 mM EDTA, 50 mM NaCl, 10 mM Tris, 0,5 mM DTT, pH 7.5) containing 10 pmol, 20 pmol or 40 pmol DoeX-protein and 2 pmol DNA (F1, F2, F3) in the presence of competitor DNA (poly dIdC, 500-fold to 1000-fold excess). After 30 min of incubation at 30°, protein–DNA complexes were resolved on a 6% polyacrylamide gel in Tris-borate EDTA buffer.

### High-performance liquid chromatography (HPLC)

For identification and quantification of intracellular amino-reactive solutes, cells were harvested, freeze-dried and extracted with chloroform/water/methanol as described previously (Galinski and Herzog, 1990). Cellular extracts were analysed on an Agilent 1100 Series reversed-phase HPLC apparatus with o-phthalaldehyde (OPA) pre-column derivatization und UV detection according to the manufacture's instructions (Agilent; http://www.chem.agilent.com/Library/chromatograms/59801193.pdf). Standards of Nγ-Ac-DABA and Nα-Ac-DABA were obtained from alkaline hydrolysis of pure ectoine (50 mM) in 10 ml 0.1 KOH for 20 h at 50°C (Kunte *et al*., 1993).

## Supporting information

**Fig. S1.** Species-level assignment of *H. elongata* proteins by MEGAN analysis. The plot indicates the number of proteins assigned to the named species out of a set of 1672 proteins for which such an assignment was successful. The number of assigned proteins is given in parenthesis after the name of the species. Only 8% of the proteins are not assigned to *C. salexigens*.

**Fig. S2.** Alignment of the *H. elongata* and *C. salexigens* chromosomes. The chromosomes of *H. elongata* and *C. salexigens* were aligned using MUMmer software (Kurtz *et al.*, 2004) and show a strong X-alignment. Such X-alignments have been described for several interspecies comparisons and attributed to genome inversions around the replication origin (Eisen *et al.*, 2000). A prominent X-alignment probably indicates that the number of such genome rearrangement events was small, which is astonishing for species with such an evolutionary distance that they are classified into distinct genera.

**Fig. S3.** Utilization of acetate as carbon source by *H. elongata* strains in the absence and presence of ectoine. H. elongata wild type, mutant strains KB41 (Δ*doeA*), KB42 (Δ*doeB*) and KB47 (Δ*doeD*) were incubated for 3 days at 30°C on mineral salt medium (0.51 M NaCl) containing 40 mM acetate (A) and 40 mM acetate plus 10 mM ectoine (B) respectively. All strains were able to grow on acetate as sole carbon source (A). In the presence of ectoine, mutant KB47 failed to grow with acetate, while KB41 and KB42 are still able to utilize acetate (B). The inability of *doeD* mutant KB47 to grow on acetate in the presence of ectoine explains why KB47 also fails to grow with ectoine alone although acetate should be still provided due to the deacetylase activity of DoeB (Fig. 3).

**Fig. S4.** RT-PCR and RACE-PCR analysis of the *doeABXC* region. (A) Genetic and physical organization of the *doeABX* locus. The position of the σ^70^-dependent promoter is indicated. Primer binding sites for RT-PCR are marked by triangles. Black triangles with dotted lines show successful generation of PCR products. For the pair of white triangles, no PCR product was obtained. Reverse primers were used for both, RT reaction and PCR. (B) RT-PCR analysis proving that *doeABX* is organized as one operon. A 1450 bp PCR product was amplified from cDNA and separated by agarose-gel electrophoresis (lane 1), which matched the size of the calculated *doeABX* PCR product (1473 bp). A corresponding *doeX–doeC* product could be amplified from genomic DNA with the same primer pair (positive control, lane 2). No PCR product could be amplified from cDNA using a primer pair to *doeX* and *doeC*, proving that *doeC* is not part of the *doeABX* operon (lane 3). A corresponding *doeX–doeC* product could be amplified from genomic DNA with the same primer pair (positive control, lane 4). A PCR product could be amplified with primers that both bind within the *doeC* ORF (control RT reaction, data not shown, product indicated in A). (C) Nucleotide sequence of the *doeA* promoter region. Arrows indicate the transcription initiation site (+ 1), which was mapped by RACE-PCR. The −35 and −10 sequences of the σ^70^-dependent promoter upstream of *doeA* are written in bold.

**Fig. S5.** Maximum ectoine yield as a function of the ATP load and turnover of ectoine in the synthesis degradation cycle (A) and flux distributions for maximum conversion into ectoine (B). (A) Points I to IV mark the flux distributions described in panel B. Horizontal axes represent total consumption of ATP by processes outside the model and the flux circulating through the ectoine synthesis/degradation cycle. The units are arbitrary, normalized for a glucose uptake of 100 (e.g. molecules). (B) I) One of the admissible flux distributions without ATP demand. II) Unique solution when total ATP demand is less than or equal to one ATP per glucose III) Example of solutions for higher ATP demands, superposition of II and maximum ATP production distribution. IV) Example of solutions for a turnover of ectoine, identical to III everywhere except the ATP load and cycle.

**Table S1.** The 20 COGs with the highest occupancy.

**Table S2.** Organisms used for the search for high salt COGs.

**Table S3.** Average pI difference between proteins from *H. elongata* and the indicated organisms.

**Table S4.** Enzymatic reactions used in the metabolic models.

**Table S5.** Bacterial strains and plasmids used in this study.

**Table S6.** Locus tag of genes similar to ect genes, *ask*, and doe genes depicted in Figs 2, 4 and 7, and accession data of genome sequences of corresponding organisms.
