# Supplementary figures and images for: A blueprint of ectoine metabolism from the genome of the industrial producer Halomonas elongata DSM 2581T
Source: Environ Microbiol. 2011 Aug;13(8):1973–94. doi: 10.1111/j.1462-2920.2010.02336.x (PMC3187862; doi:10.1111/j.1462-2920.2010.02336.x)

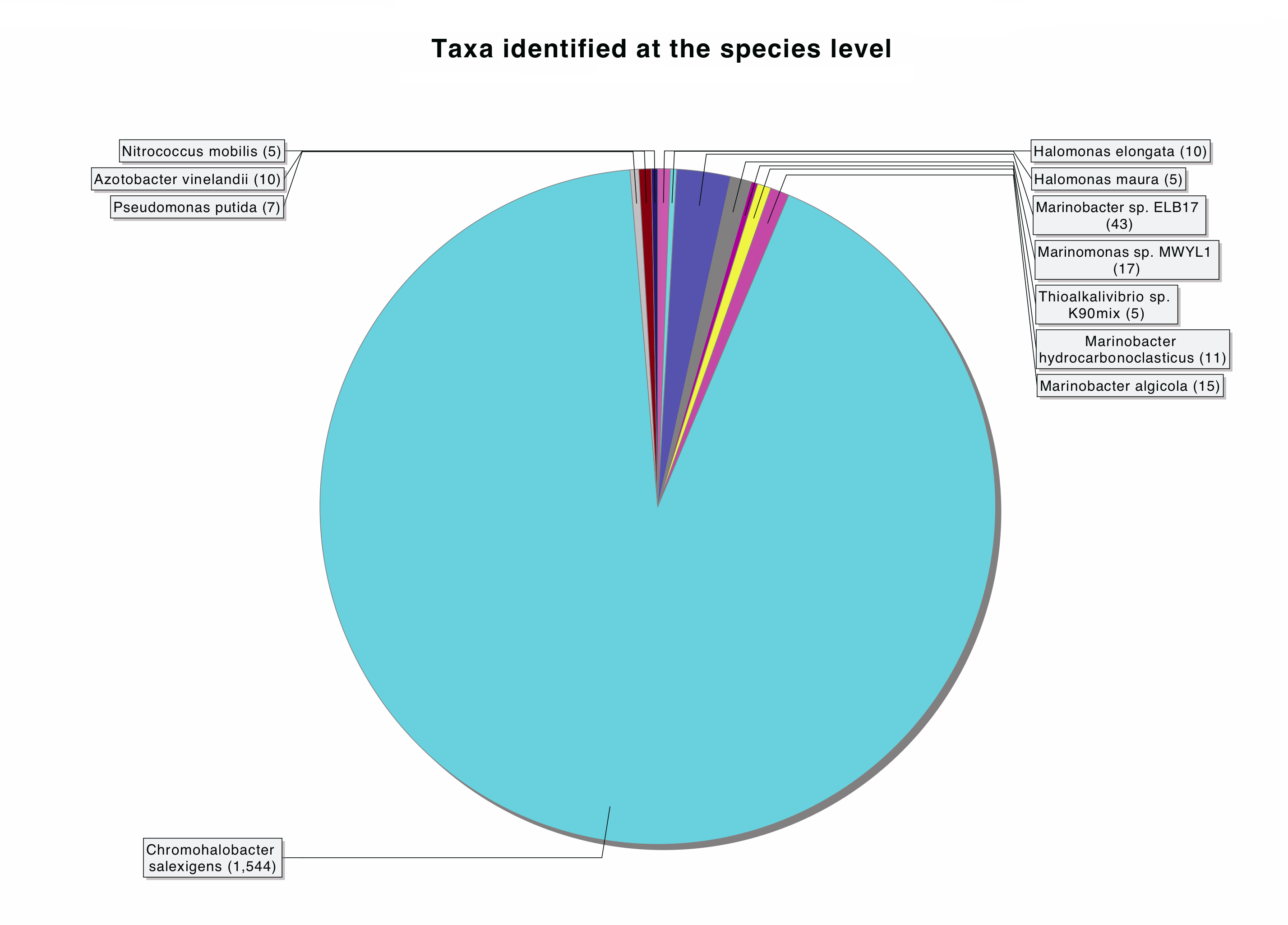

Supplement: Supplementary file 1 [file emi0013-1973-SD1.tif]

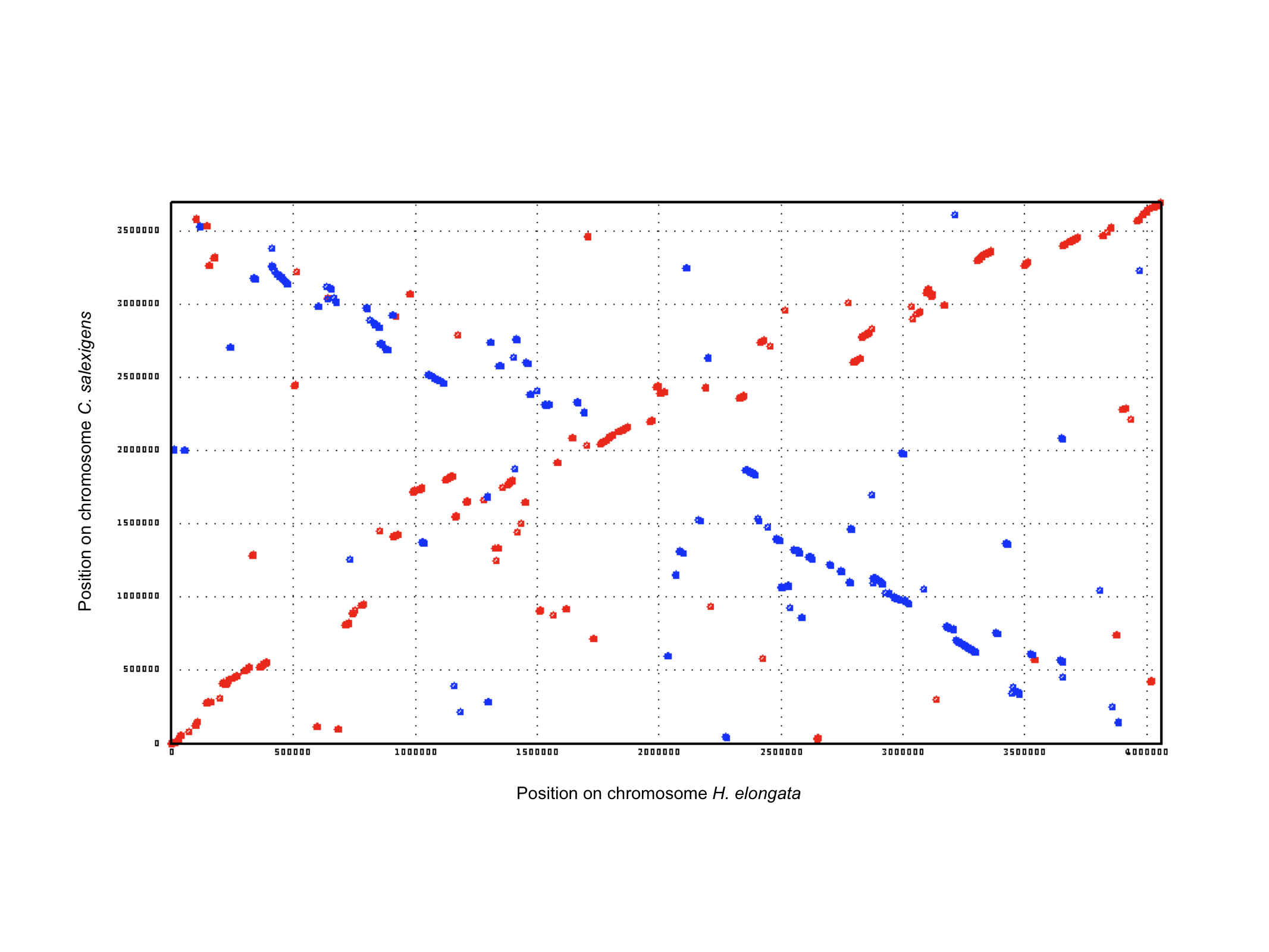

Supplement: Supplementary file 2 [file emi0013-1973-SD2.tif]

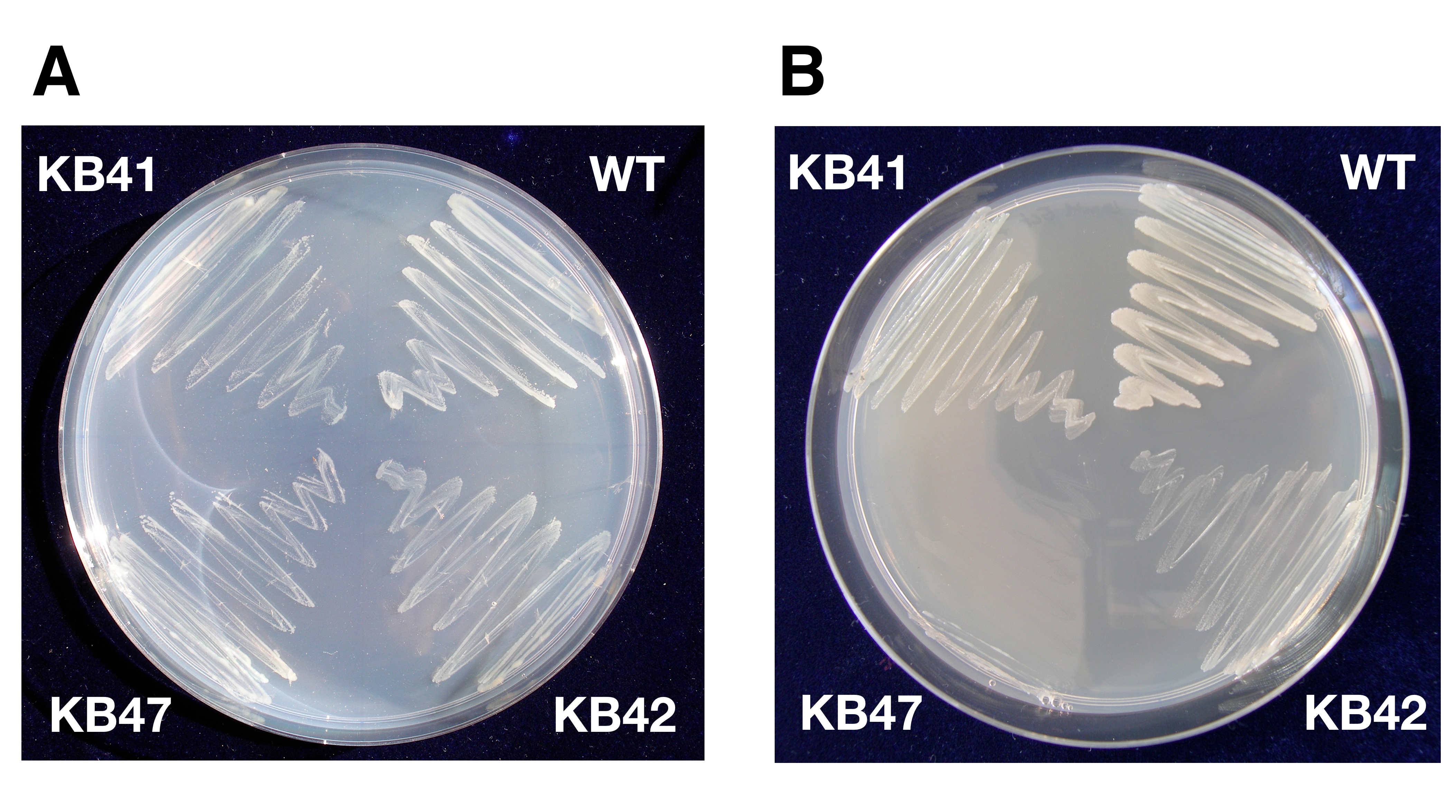

Supplement: Supplementary file 3 [file emi0013-1973-SD3.tif]

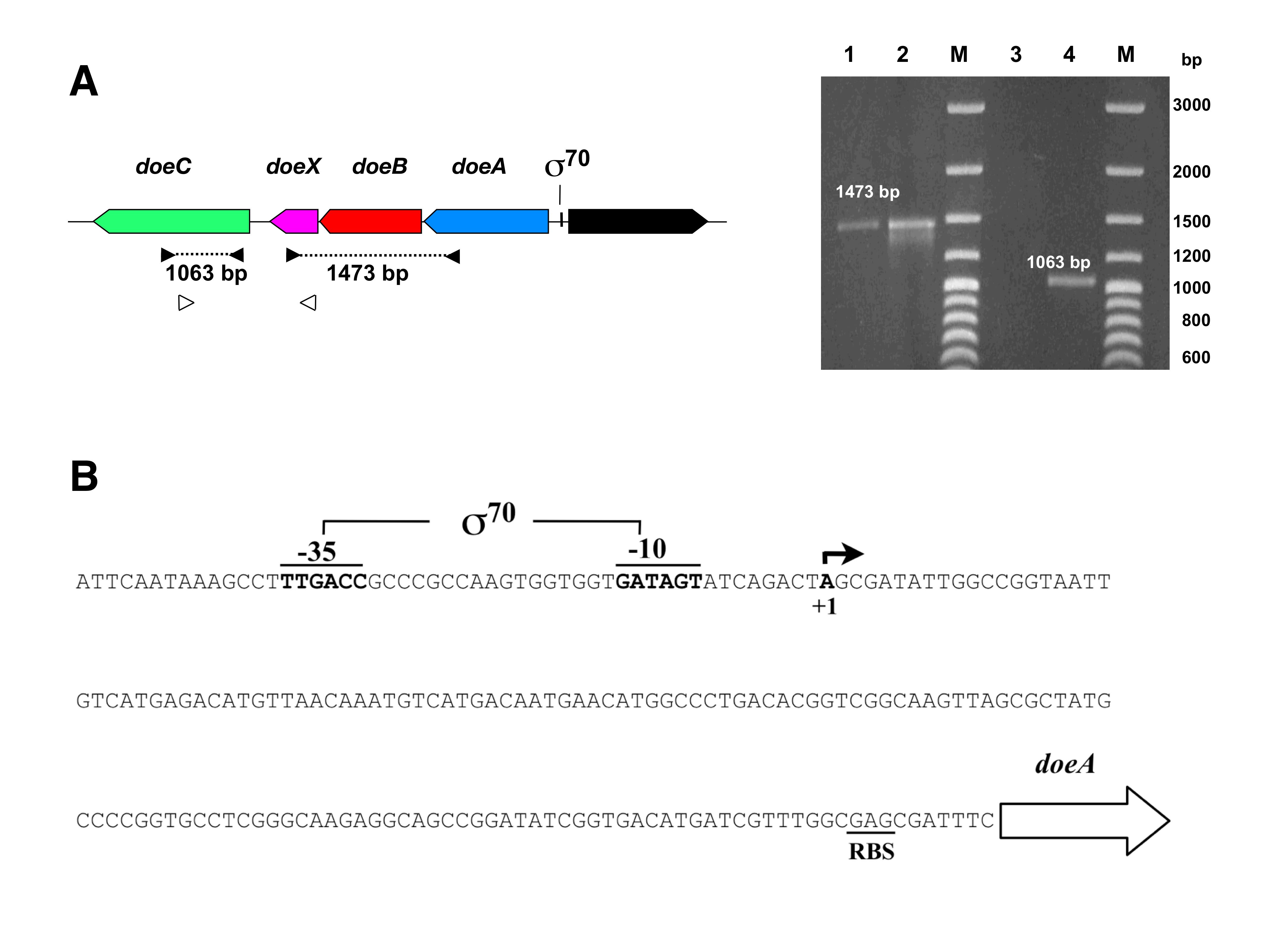

Supplement: Supplementary file 4 [file emi0013-1973-SD4.tif]

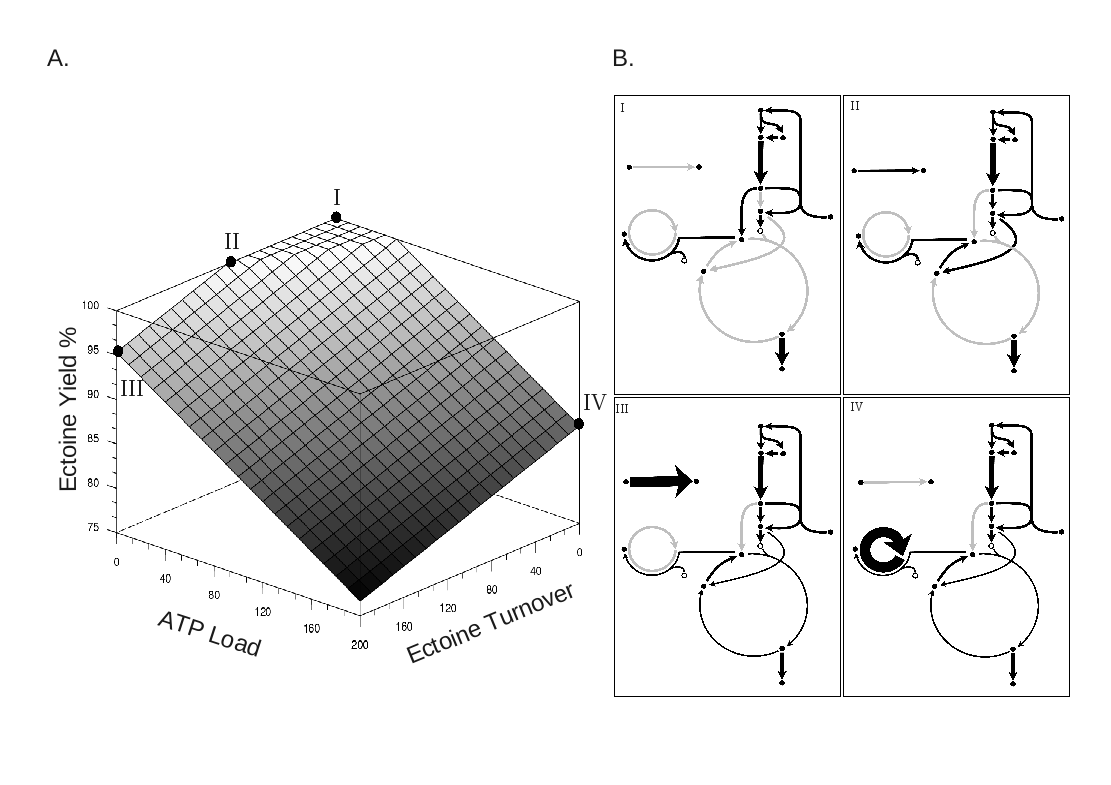

Supplement: Supplementary file 5 [file emi0013-1973-SD5.tif]
