# Supplementary material for: A blueprint of ectoine metabolism from the genome of the industrial producer Halomonas elongata DSM 2581T
Source: Environ Microbiol. 2011 Aug;13(8):1973–94. doi: 10.1111/j.1462-2920.2010.02336.x (PMC3187862; doi:10.1111/j.1462-2920.2010.02336.x)
Supplement: Supplementary file 6 [file emi0013-1973-SD6.doc]

**Figure S1.** Species-level assignment of *H. elongata* proteins by MEGAN analysis. The plot indicates the number of proteins assigned to the named species out of a set of 1,672 proteins for which such an assignment was successful. The number of assigned proteins is given in parenthesis after the name of the species. Only 8% of the proteins are not assigned to *C. salexigens*.

**Figure S2.** Alignment of the *H. elongata* and *C. salexigens* chromosomes. The chromosomes of *H. elongata* and *C. salexigens* were aligned using MUMmer software (Kurtz et al., 2004) and show a strong X-alignment. Such X-alignments have been described for several interspecies comparisons and attributed to genome inversions around the replication origin (Eisen et al., 2000). A prominent X-alignment probably indicates that the number of such genome rearrangement events was small, which is astonishing for species with such an evolutionary distance that they are classified into distinct genera.

**Figure S3.** Utilization of acetate as carbon source by *H. elongata* strains in the absence and presence of ectoine. *H. elongata* wild type, mutant strains KB41 (Δ*doeA*), KB42 (Δ*doeB*), and KB47 (Δ*doeD*) were incubated for three days at 30° C on mineral salt medium (0.51 M NaCl) containing 40 mM acetate (A) and 40 mM acetate plus 10 mM ectoine (B), respectively. All strains were able to grow on acetate as sole carbon source (A). In the presence of ectoine, mutant KB47 failed to grow with acetate, while KB41 and KB42 are still able to utilize acetate (B). The inability of *doeD* mutant KB47 to grow on acetate in the presence of ectoine explains why KB47 also fails to grow with ectoine alone although acetate should be still provided due to the deacetylase activity of DoeB (Figure 3).

**Figure S4.** RT-PCR and RACE-PCR analysis of the *doeABXC* region. (A) Genetic and physical organization of the *doeABX* locus. The position of the σ^70^-dependent promoter is indicated. Primer binding-sites for RT-PCR are marked by triangles. Black triangles with dotted lines show successful generation of PCR products. For the pair of white triangles, no PCR product was obtained. Reverse primers were used for both, RT reaction and PCR. (B) RT-PCR analysis proving that *doeABX* is organized as one operon. A 1450 bp PCR product was amplified from cDNA and separated by agarose-gel electrophoresis (lane 1), which matched the size of the calculated *doeABX* PCR product (1473 bp). A corresponding *doeX-doeC* product could be amplified from genomic DNA with the same primer pair (positive control, lane 2). No PCR product could be amplified from cDNA using a primer pair to *doeX* and *doeC,* proving that *doeC* is not part of the *doeABX* operon (lane 3). A corresponding *doeX-doeC* product could be amplified from genomic DNA with the same primer pair (positive control, lane 4). A PCR product could be amplified with primers that both bind within the *doeC* ORF (control RT reaction, data not shown, product indicated in A). (C) Nucleotide sequence of the *doeA* promoter region. Arrows indicate the transcription initiation-site (+1), which was mapped by RACE-PCR. The –35 and –10 sequences of the σ^70^-dependent promoter upstream of *doeA* are written in bold.

**Figure S5. Maximum ectoine yield as a function of the ATP load and turnover of ectoine in the synthesis degradation cycle (A) and flux distributions for maximum conversion into ectoine (B).** (A) Points I to IV mark the flux distributions described in panel B. Horizontal axes represent total consumption of ATP by processes outside the model and the flux circulating through the ectoine synthesis/degradation cycle. The units are arbitrary, normalized for a glucose uptake of 100 (e.g. molecules). (B) I) One of the admissible flux distributions without ATP demand. II) Unique solution when total ATP demand is less than or equal to one ATP per glucose III) Example of solutions for higher ATP demands, superposition of II and maximum ATP production distribution. IV) Example of solutions for a turnover of ectoine, identical to III everywhere except the ATP load and cycle.
