# Supplementary material for: A blueprint of ectoine metabolism from the genome of the industrial producer Halomonas elongata DSM 2581T
Source: Environ Microbiol. 2011 Aug;13(8):1973–94. doi: 10.1111/j.1462-2920.2010.02336.x (PMC3187862; doi:10.1111/j.1462-2920.2010.02336.x)
Supplement: Supplementary file 7 [file emi0013-1973-SD7.doc]

**Table S1.** The 20 COGs with the highest occupancy.

Shown are the numbers of proteins in *H. elongata* (#(H.elo)) and in *C. salexigens* (#(C.sal)), as well as the number of bidirectional best blast pairs. All of the 20 highest occupancy COGs belong to the four categories “transcription regulators”, broad-specificity enzymes, transporters and their subunits and two-component systems. A high fraction of the COG members are ortholog pairs in *H. elongata* and *C. salexigens*. Commonly, the number of COG members is similar in the two organisms.

| COG | category | #(H.elo) | #(C.sal) | pairs | COG description |
| --- | --- | --- | --- | --- | --- |
| COG0583 | regulator | 57 | 52 | 36 | Transcriptional regulator |
| COG1028 | enzyme | 26 | 29 | 14 | Dehydrogenases with different specificities (related to short-chain alcohol dehydrogenases) |
| COG0477 | transporter | 25 | 22 | 15 | Permeases of the major facilitator superfamily |
| COG1638 | transporter | 23 | 18 | 13 | TRAP-type C4-dicarboxylate transport system, periplasmic component |
| COG1012 | enzyme | 22 | 20 | 12 | NAD-dependent aldehyde dehydrogenases |
| COG0642 | two-comp. | 21 | 17 | 14 | Signal transduction histidine kinase |
| COG1593 | transporter | 21 | 15 | 11 | TRAP-type C4-dicarboxylate transport system, large permease component |
| COG1309 | regulator | 16 | 10 | 9 | Transcriptional regulator |
| COG0745 | two-comp. | 14 | 11 | 9 | Response regulators consisting of a CheY-like receiver domain and a winged-helix DNA-binding domain |
| COG0454 | enzyme | 14 | 10 | 10 | Histone acetyltransferase HPA2 and related acetyltransferases |
| COG0697 | transporter | 12 | 11 | 7 | Permeases of the drug/metabolite transporter (DMT) superfamily |
| COG2207 | regulator | 12 | 11 | 8 | AraC-type DNA-binding domain-containing proteins |
| COG0500 | enzyme | 12 | 9 | 8 | SAM-dependent methyltransferases |
| COG4977 | regulator | 11 | 5 | 4 | Transcriptional regulator containing an amidase domain and an AraC-type DNA-binding HTH domain |
| COG0665 | enzyme | 10 | 10 | 9 | Glycine/D-amino acid oxidases (deaminating) |
| COG1802 | regulator | 10 | 10 | 7 | Transcriptional regulators |
| COG3090 | transporter | 10 | 9 | 6 | TRAP-type C4-dicarboxylate transport system, small permease component |
| COG1396 | regulator | 10 | 7 | 5 | Predicted transcriptional regulators |
| COG3839 | transporter | 10 | 6 | 5 | ABC-type sugar transport systems, ATPase components |
| COG0609 | transporter | 9 | 7 | 6 | ABC-type Fe3+-siderophore transport system, permease component |

**Table S2.** Organisms used for the search for high salt COGs.

The halophilic (H), marine (M), and standard (S) organisms used in the genome comparison analysis are listed.

| taxonomic branch | halophilic/marine | H/M/S | TaxID | standard organism |
| --- | --- | --- | --- | --- |
| [Firmicutes](http://www.ncbi.nlm.nih.gov/Taxonomy/Browser/wwwtax.cgi?mode=Info&id=1239&lvl=6&lin=f&keep=1&srchmode=5&unlock&filter=genome_filter) | *Bacillus halodurans* C-125 | H | 272558 |  |
| [Firmicutes](http://www.ncbi.nlm.nih.gov/Taxonomy/Browser/wwwtax.cgi?mode=Info&id=1239&lvl=6&lin=f&keep=1&srchmode=5&unlock&filter=genome_filter) | *Oceanobacillus iheyensis* HTE831 | M | 221109 |  |
| [Firmicutes](http://www.ncbi.nlm.nih.gov/Taxonomy/Browser/wwwtax.cgi?mode=Info&id=1239&lvl=6&lin=f&keep=1&srchmode=5&unlock&filter=genome_filter) |  | S | 224308 | *Bacillus subtilis* subsp. *subtilis* str. 168 |
| [Firmicutes](http://www.ncbi.nlm.nih.gov/Taxonomy/Browser/wwwtax.cgi?mode=Info&id=1239&lvl=6&lin=f&keep=1&srchmode=5&unlock&filter=genome_filter) |  | S | 222523 | *Bacillus cereus* ATCC 10987 |
| [Bacteroidetes](http://www.ncbi.nlm.nih.gov/Taxonomy/Browser/wwwtax.cgi?mode=Info&id=68336&lvl=6&lin=f&keep=1&srchmode=5&unlock&filter=genome_filter) | *Salinibacter ruber* DSM 13855 | H | 309807 |  |
| [Gamma-proteobacteria](http://www.ncbi.nlm.nih.gov/Taxonomy/Browser/wwwtax.cgi?mode=Info&id=1224&lvl=6&lin=f&keep=1&srchmode=5&unlock&filter=genome_filter) | *Chromohalobacter salexigens* DSM 3043 | H | 290398 |  |
| [Gamma-proteobacteria](http://www.ncbi.nlm.nih.gov/Taxonomy/Browser/wwwtax.cgi?mode=Info&id=1224&lvl=6&lin=f&keep=1&srchmode=5&unlock&filter=genome_filter) | *Hahella chejuensis* KCTC 2396 | M | 349521 |  |
| [Gamma-proteobacteria](http://www.ncbi.nlm.nih.gov/Taxonomy/Browser/wwwtax.cgi?mode=Info&id=1224&lvl=6&lin=f&keep=1&srchmode=5&unlock&filter=genome_filter) | *Marinobacter aquaeolei* VT8 | M | 351348 |  |
| [Gamma-proteobacteria](http://www.ncbi.nlm.nih.gov/Taxonomy/Browser/wwwtax.cgi?mode=Info&id=1224&lvl=6&lin=f&keep=1&srchmode=5&unlock&filter=genome_filter) | *Marinomonas sp*. MWYL1 | M | 400668 |  |
| [Gamma-proteobacteria](http://www.ncbi.nlm.nih.gov/Taxonomy/Browser/wwwtax.cgi?mode=Info&id=1224&lvl=6&lin=f&keep=1&srchmode=5&unlock&filter=genome_filter) | *Shewanella denitrificans* OS217 | M | 318161 |  |
| [Gamma-proteobacteria](http://www.ncbi.nlm.nih.gov/Taxonomy/Browser/wwwtax.cgi?mode=Info&id=1224&lvl=6&lin=f&keep=1&srchmode=5&unlock&filter=genome_filter) | *Vibrio fischeri* ES114 | M | 312309 |  |
| [Gamma-proteobacteria](http://www.ncbi.nlm.nih.gov/Taxonomy/Browser/wwwtax.cgi?mode=Info&id=1224&lvl=6&lin=f&keep=1&srchmode=5&unlock&filter=genome_filter) | *Nitrosococcus oceani* ATCC 19707 | M | 323261 |  |
| [Gamma-proteobacteria](http://www.ncbi.nlm.nih.gov/Taxonomy/Browser/wwwtax.cgi?mode=Info&id=1224&lvl=6&lin=f&keep=1&srchmode=5&unlock&filter=genome_filter) |  | S | 220664 | *Pseudomonas fluorescens* Pf-5 |
| [Gamma-proteobacteria](http://www.ncbi.nlm.nih.gov/Taxonomy/Browser/wwwtax.cgi?mode=Info&id=1224&lvl=6&lin=f&keep=1&srchmode=5&unlock&filter=genome_filter) |  | S | 83333 | *Escherichia coli* K12 |
| [Gamma-proteobacteria](http://www.ncbi.nlm.nih.gov/Taxonomy/Browser/wwwtax.cgi?mode=Info&id=1224&lvl=6&lin=f&keep=1&srchmode=5&unlock&filter=genome_filter) |  | S | 399741 | *Serratia proteamaculans* 568 |
| [Gamma-proteobacteria](http://www.ncbi.nlm.nih.gov/Taxonomy/Browser/wwwtax.cgi?mode=Info&id=1224&lvl=6&lin=f&keep=1&srchmode=5&unlock&filter=genome_filter) |  | S | 297246 | *Legionella pneumophila* str. Paris |
| Alpha-p[roteobacteria](http://www.ncbi.nlm.nih.gov/Taxonomy/Browser/wwwtax.cgi?mode=Info&id=1224&lvl=6&lin=f&keep=1&srchmode=5&unlock&filter=genome_filter) | *Silicibacter sp.* TM1040 | M | 292414 |  |
| Alpha-p[roteobacteria](http://www.ncbi.nlm.nih.gov/Taxonomy/Browser/wwwtax.cgi?mode=Info&id=1224&lvl=6&lin=f&keep=1&srchmode=5&unlock&filter=genome_filter) | *Roseobacter denitrificans* OCh 114 | M | 375451 |  |
| Alpha-p[roteobacteria](http://www.ncbi.nlm.nih.gov/Taxonomy/Browser/wwwtax.cgi?mode=Info&id=1224&lvl=6&lin=f&keep=1&srchmode=5&unlock&filter=genome_filter) |  | S | 176299 | *Agrobacterium tumefaciens* str. C58 |
| Alpha-p[roteobacteria](http://www.ncbi.nlm.nih.gov/Taxonomy/Browser/wwwtax.cgi?mode=Info&id=1224&lvl=6&lin=f&keep=1&srchmode=5&unlock&filter=genome_filter) |  | S | 264203 | *Zymomonas mobilis* subsp. *mobilis* ZM4 |
| Alpha-p[roteobacteria](http://www.ncbi.nlm.nih.gov/Taxonomy/Browser/wwwtax.cgi?mode=Info&id=1224&lvl=6&lin=f&keep=1&srchmode=5&unlock&filter=genome_filter) |  | S | 269796 | *Rhodospirillum rubrum* ATCC 11170 |
| Alpha-p[roteobacteria](http://www.ncbi.nlm.nih.gov/Taxonomy/Browser/wwwtax.cgi?mode=Info&id=1224&lvl=6&lin=f&keep=1&srchmode=5&unlock&filter=genome_filter) |  | S | 439375 | *Ochrobactrum anthropi* ATCC 49188 |
| [Beta-proteobacteria](http://www.ncbi.nlm.nih.gov/Taxonomy/Browser/wwwtax.cgi?mode=Info&id=1224&lvl=6&lin=f&keep=1&srchmode=5&unlock&filter=genome_filter) |  | S | 375286 | *Janthinobacterium* sp. Marseille |
| [Beta-proteobacteria](http://www.ncbi.nlm.nih.gov/Taxonomy/Browser/wwwtax.cgi?mode=Info&id=1224&lvl=6&lin=f&keep=1&srchmode=5&unlock&filter=genome_filter) |  | S | 228410 | *Nitrosomonas europaea* ATCC 19718 |
| [Cyanobacteria](http://www.ncbi.nlm.nih.gov/Taxonomy/Browser/wwwtax.cgi?mode=Info&id=1117&lvl=3&lin=f&keep=1&srchmode=5&unlock&filter=genome_filter) | *Prochlorococcus marinus* str. MIT 9313 | M | 74547 |  |
| [Cyanobacteria](http://www.ncbi.nlm.nih.gov/Taxonomy/Browser/wwwtax.cgi?mode=Info&id=1117&lvl=3&lin=f&keep=1&srchmode=5&unlock&filter=genome_filter) |  | S | 103690 | *Nostoc sp*. PCC 7120 |
| [Aquificae](http://www.ncbi.nlm.nih.gov/Taxonomy/Browser/wwwtax.cgi?mode=Undef&id=200783&lvl=3&keep=1&srchmode=5&unlock&filter=genome_filter) |  | S | 224324 | *Aquifex aeolicus* VF5 |

### Table S3. Average pI difference between proteins from *H. elongata* and the indicated organisms.

The pI differences were computed for the 27 halophilic (H), marine (M), and standard (S) organisms listed in Table S2. Protein pair selection is based on COG assignments. Each COG that contains a single protein in *H. elongata* and in the indicated organism was included in the analysis. The number of included COGs (and thus protein pairs) is indicated. The pI of the two proteins and their difference was computed. The pI differences were averaged (pI shift). Negative numbers indicate that the average pI of the compared organism is more acidic, positive number that it is more alkaline. The table is sorted by pI shift. It should be noted that most of the pI shifts are only minimal but that only two organisms have a shift towards a more acidic proteome.

| Organism | H/M/S | pI shift | # pairs |
| --- | --- | --- | --- |
| *Salinibacter ruber* | H | -0.7 | 524 |
| *Oceanobacillus iheyensis* | M | -0.1 | 561 |
| *Marinobacter aquaeolei* | M | 0.0 | 952 |
| *Silicibacter sp*. TM1040 | M | 0.0 | 659 |
| Chromohalobacter salexigens | H | 0.1 | 1114 |
| *Marinomonas sp.* | M | 0.1 | 924 |
| *Vibrio fischeri* | M | 0.1 | 730 |
| *Roseobacter denitrificans* | M | 0.1 | 714 |
| *Bacillus halodurans* | H | 0.1 | 615 |
| *Bacillus cereus* | S | 0.1 | 549 |
| *Hahella chejuensis* | M | 0.2 | 926 |
| *Shewanella denitrificans* | M | 0.2 | 832 |
| *Bacillus subtilis* | S | 0.2 | 594 |
| *Nostoc sp*. PCC 7120 | S | 0.2 | 529 |
| Escherichia coli | S | 0.4 | 855 |
| *Agrobacterium tumefaciens* | S | 0.4 | 608 |
| *Ochrobactrum anthropi* | S | 0.4 | 574 |
| *Pseudomonas fluorescens* | S | 0.5 | 920 |
| *Serratia proteamaculans* | S | 0.5 | 866 |
| *Rhodospirillum rubrum* | S | 0.5 | 679 |
| *Prochlorococcus marinus* | M | 0.5 | 517 |
| *Nitrosomonas europaea* | S | 0.6 | 674 |
| *Janthinobacterium* sp. Marseille | S | 0.7 | 752 |
| Zymomonas mobilis | S | 0.8 | 566 |
| *Nitrosococcus oceani* | M | 0.9 | 755 |
| *Legionella pneumophila* | S | 0.9 | 670 |
| *Aquifex aeolicus* | S | 1.3 | 508 |

### Table S4. Enzymatic reactions used in the metabolic models.

The table contains the reaction number, reaction name, and the catalyzed reaction (columns 3 to 5, data taken from KEGG). In the first two columns, the EC number and the annotated *H. elongata* protein(s) are indicated.

##### Glycine, Serine and Threonin metabolism

| ec:2.7.1.39 | Helo_4004 | R01771 | ATP:L-homoserine O-phosphotransferase | L-Homoserine + ATP ===> O-Phospho-L-homoserine + ADP | |
| --- | --- | --- | --- | --- | --- |
| ec:4.2.1.108 | Helo_2590 | R06979 | Ectoine hydro-lyase | N-gamma-Acetyldiaminobutyrate ===> Ectoine + H2O | |
| ec:2.6.1.52 | Helo_2720 | R04173 | 3-Phosphoserine:2-oxoglutarate aminotransferase | Dexfosfoserine + 2-Oxoglutarate <===> Glutamate + 3Phosphohydroxypyruvate | |
| ec:2.6.1.76 | Helo_2589 | R06977 | L-2,4-diaminobutyrate:2-oxoglutarate 4-aminotransferase | Glutamate + L-Aspartic 4-semialdehyde <===> L-2,4-Diaminobutyrate + 2-Oxoglutarate | |
| ec:3.1.3.3 | Helo_1722 | R00582 | O-phospho-L-serine phosphohydrolase | Dexfosfoserine + H2O ===> Phosphate + Serine | |
| ec:1.1.1.95 | Helo_1081 Helo_3667 | R01513 | 3-Phospho-D-glycerate:NAD+ 2-oxidoreductase | NAD + 3-Phosphoglycerate <===> 3Phosphohydroxypyruvate + NADH + H+ | |
| ec:2.1.2.1 | Helo_3598 Helo_3449 | R00945 | 5,10-Methylenetetrahydrofolate:glycine hydroxymethyltransferase | Gly + H2O + 5,10-Methylene-THF <===> THF + Serine |  |
| ec:4.2.3.1 | Helo_3856 | R01466 | L-threonine-forming) | H2O + O-Phospho-L-homoserine <===> L-Threonine + Phosphate |  |
| ec:1.1.1.3 | Helo_3857 | R01773 | L-Homoserine:NAD+ oxidoreductase | L-Homoserine + NAD <===> L-Aspartic 4-semialdehyde + NADH + H+ |  |
| ec:2.1.2.10 | Helo_2529 Helo_1668 Helo_2544 | R04125 | S-aminomethyl-dihydrolipoylprotein:(6S)-tetrahydrofolate aminomethyltransferase (ammonia-forming) | THF + S-Aminomethyldihydrolipoylprotein <===> NH3 + Dihydrolipoylprotein + 5,10-Methylene-THF |  |
| ec:4.3.1.17 | Helo_3468 | R00220 | L-serine ammonia-lyase | Serine ===> NH3 + Pyruvate |  |
| ec:1.8.1.4 | Helo_3110 | R08549 | 2-Oxoglutarate dehydrogenase complex | CoA + NAD + 2-Oxoglutarate <===> CO2 + NADH + H+ + Succinyl-CoA |  |
| ec:1.8.1.4 | Helo_3110 | R00209 | pyruvate dehydrogenase complex | Pyruvate + CoA + NAD <===> CO2 + Acetyl-CoA + NADH + H+ |  |
| ec:2.3.1.178 | Helo_2588 | R06978 | L-2,4-diaminobutyrate acetyltransferase | L-2,4-Diaminobutyrate + Acetyl-CoA ===> CoA + N-gamma-Acetyldiaminobutyrate |  |
| ec:2.7.2.4 | Helo_3742 | R00480 | ATP:L-aspartate 4-phosphotransferase | L-Aspartate + ATP ===> 4-Phospho-L-aspartate + ADP |  |
| ec:1.2.1.11 | Helo_2235 | R02291 | L-Aspartate-4-semialdehyde:NADP+ oxidoreductase (phosphorylating) | L-Aspartic 4-semialdehyde + NADP + Phosphate <===> 4-Phospho-L-aspartate + NADPH + H+ |  |
| ec:4.3.1.19 | Helo_1021 Helo_3660 Helo_3251 | R00220 | L-serine ammonia-lyase | Serine ===> NH3 + Pyruvate |  |

TCA cycle

| ec:2.3.3.1 | Helo_3117 | R00351 | acetyl-CoA:oxaloacetate C-acetyltransferase (thioester-hydrolysing) | CoA + Citrate <=== Acetyl-CoA + H2O + Oxaloacetate |
| --- | --- | --- | --- | --- |
| ec:4.1.1.49 | Helo_1685 | R00341 | phosphoenolpyruvate-forming) | ATP + Oxaloacetate ===> PEP + CO2 + ADP |
| ec:4.2.1.3 | Helo_2076 Helo_2437 Helo_3816 | R01900 | isocitrate hydro-lyase (cis-aconitate-forming) | Isocitrate <===> H2O + cis-Aconitate |
| ec:4.2.1.3 | Helo_2076 Helo_2437 Helo_3816 | R01325 | citrate hydro-lyase (cis-aconitate-forming) | Citrate <===> H2O + cis-Aconitate |
| ec:4.2.1.2 | Helo_2298 Helo_2547 | R01082 | (S)-malate hydro-lyase (fumarate-forming) | Malate <===> H2O + Fumarate |
| ec:1.2.4.2 | Helo_4075 Helo_3112 | R00621 | rn:R00621 | TPP + 2-Oxoglutarate <===> CO2 + 3-Carboxy-1-hydroxypropyl-ThPP |
| ec:1.2.4.2 | Helo_4075 Helo_3112 | R08549 | 2-Oxoglutarate dehydrogenase complex | CoA + NAD + 2-Oxoglutarate <===> CO2 + NADH + H+ + Succinyl-CoA |
| ec:1.2.4.1 | Helo_3572 | R00209 | pyruvate dehydrogenase complex | Pyruvate + CoA + NAD <===> CO2 + Acetyl-CoA + NADH + H+ |
| ec:1.3.99.1 | Helo_3114 Helo_3113 Helo_3115 Helo_3116 | R00412 | succinate:acceptor oxidoreductase | Succinate + A <===> AH2 + Fumarate |
| ec:1.1.1.42 | Helo_3063 Helo_3252 | R00268 | oxalosuccinate carboxy-lyase (2-oxoglutarate-forming) | Oxalosuccinate <===> CO2 + 2-Oxoglutarate |
| ec:1.1.1.42 | Helo_3063 Helo_3252 | R01899 | Isocitrate:NADP+ oxidoreductase | Isocitrate + NADP <===> Oxalosuccinate + NADPH + H+ |
| ec:2.3.1.12 | Helo_3571 Helo_2372 | R00209 | pyruvate dehydrogenase complex | Pyruvate + CoA + NAD <===> CO2 + Acetyl-CoA + NADH + H+ |
| ec:6.2.1.5 | Helo_2498 Helo_2634 Helo_3108 Helo_3109 | R00405 | Succinate:CoA ligase (ADP-forming) | CoA + ATP + Succinate <===> Phosphate + ADP + Succinyl-CoA |
| ec:1.8.1.4 | Helo_3110 | R08549 | 2-Oxoglutarate dehydrogenase complex | CoA + NAD + 2-Oxoglutarate <===> CO2 + NADH + H+ + Succinyl-CoA |
| ec:1.8.1.4 | Helo_3110 | R00209 | pyruvate dehydrogenase complex | Pyruvate + CoA + NAD <===> CO2 + Acetyl-CoA + NADH + H+ |
| ec:2.3.1.61 | Helo_3111 Helo_2296 | R08549 | 2-Oxoglutarate dehydrogenase complex | CoA + NAD + 2-Oxoglutarate <===> CO2 + NADH + H+ + Succinyl-CoA |
| ec:1.1.1.37 | Helo_3404 Helo_1693 | R00342 | (S)-malate:NAD+ oxidoreductase | NAD + Malate <===> NADH + H+ + Oxaloacetate |

Pyruvate metabolism

| ec:2.7.9.2 | Helo_2433 | R00199 | ATP:pyruvate,water phosphotransferase | Pyruvate + H2O + ATP ===> PEP + AMP + Phosphate |
| --- | --- | --- | --- | --- |
| ec:4.1.1.49 | Helo_1685 | R00341 | phosphoenolpyruvate-forming) | ATP + Oxaloacetate ===> PEP + CO2 + ADP |
| ec:4.1.1.3 | Helo_3734 Helo_3736 Helo_3735 | R00217 | oxaloacetate carboxy-lyase (pyruvate-forming) | Oxaloacetate ===> Pyruvate + CO2 |
| ec:2.3.3.9 | Helo_4288 | R00472 | L-Malate glyoxylate-lyase (CoA-acetylating) | CoA + Malate <=== Glyoxylate + Acetyl-CoA + H2O |
| ec:4.1.1.31 | Helo_3010 | R00345 | phosphoenolpyruvate-forming) | Phosphate + Oxaloacetate <=== PEP + CO2 + H2O |
| ec:1.2.4.1 | Helo_3572 | R00209 | pyruvate dehydrogenase complex | Pyruvate + CoA + NAD <===> CO2 + Acetyl-CoA + NADH + H+ |
| ec:1.2.1.3 | Helo_2817 | R00710 | Acetaldehyde:NAD+ oxidoreductase | Acetaldehyde + H2O + NAD <===> NADH + H+ + Acetate |
| ec:2.7.1.40 | Helo_4243 Helo_1605 | R00200 | ATP:pyruvate 2-O-phosphotransferase | Pyruvate + ATP <=== PEP + ADP |
| ec:2.3.1.12 | Helo_3571 Helo_2372 | R00209 | pyruvate dehydrogenase complex | Pyruvate + CoA + NAD <===> CO2 + Acetyl-CoA + NADH + H+ |
| ec:1.1.1.40 | Helo_3763 | R00217 | oxaloacetate carboxy-lyase (pyruvate-forming) | Oxaloacetate ===> Pyruvate + CO2 |
| ec:1.8.1.4 | Helo_3110 | R08549 | 2-Oxoglutarate dehydrogenase complex | CoA + NAD + 2-Oxoglutarate <===> CO2 + NADH + H+ + Succinyl-CoA |
| ec:1.8.1.4 | Helo_3110 | R00209 | pyruvate dehydrogenase complex | Pyruvate + CoA + NAD <===> CO2 + Acetyl-CoA + NADH + H+ |
| ec:6.2.1.1 | Helo_2142 Helo_3563 | R00316 | ATP:acetate adenylyltransferase | ATP + Acetate <===> PPi + Acetyl adenylate |
| ec:6.2.1.1 | Helo_2142 Helo_3563 | R00236 | acetyl adenylate:CoA acetyltransferase | CoA + Acetyl adenylate <===> Acetyl-CoA + AMP |
| ec:1.1.1.37 | Helo_3404 Helo_1693 | R00342 | (S)-malate:NAD+ oxidoreductase | NAD + Malate <===> NADH + H+ + Oxaloacetate |
| ec:1.1.1.38 | Helo_3817 | R00214 | (S)-malate:NAD+ oxidoreductase (decarboxylating) | NAD + Malate <===> Pyruvate + CO2 + NADH + H+ |
| ec:1.1.1.38 | Helo_3817 | R00217 | oxaloacetate carboxy-lyase (pyruvate-forming) | Oxaloacetate ===> Pyruvate + CO2 |
| ec:1.1.1.27 | Helo_1046 | R00703 | (S)-Lactate:NAD+ oxidoreductase | NAD + L-Lactate <===> Pyruvate + NADH + H+ |

Glycolysis and Gluconeogenesis

| ec:5.3.1.9 | Helo_1718 Helo_4245 | R02739 | alpha-D-Glucose 6-phosphate ketol-isomerase | alpha-D-Glucose 6-phosphate <===> beta-D-Glucose 6-phosphate |
| --- | --- | --- | --- | --- |
| ec:5.3.1.9 | Helo_1718 Helo_4245 | R02740 | alpha-D-Glucose 6-phosphate ketol-isomerase | alpha-D-Glucose 6-phosphate <===> beta-D-Fructose 6-phosphate |
| ec:4.1.1.49 | Helo_1685 | R00341 | phosphoenolpyruvate-forming) | ATP + Oxaloacetate ===> PEP + CO2 + ADP |
| ec:5.4.2.1 | Helo_1030 Helo_1820 Helo_3004 | R01518 | 2-Phospho-D-glycerate 2,3-phosphomutase | 2-Phospho-D-glycerate <===> 3-Phosphoglycerate |
| ec:5.3.1.1 | Helo_4141 | R01015 | D-glyceraldehyde-3-phosphate aldose-ketose-isomerase | Glyceraldehyde 3-phosphate <===> Glycerone phosphate |
| ec:1.2.4.1 | Helo_3572 | R00209 | pyruvate dehydrogenase complex | Pyruvate + CoA + NAD <===> CO2 + Acetyl-CoA + NADH + H+ |
| ec:4.2.1.11 | Helo_3749 | R00658 | 2-phospho-D-glycerate hydro-lyase (phosphoenolpyruvate-forming) | 2-Phospho-D-glycerate <===> PEP + H2O |
| ec:1.2.1.3 | Helo_2817 | R00710 | Acetaldehyde:NAD+ oxidoreductase | Acetaldehyde + H2O + NAD <===> NADH + H+ + Acetate |
| ec:2.7.1.69 | Helo_3696 Helo_1921 Helo_3099 | R02738 | Protein-N(pai)-phosphohistidine:sugar N(pai)-phosphotransferase | Protein N-pros-phosphohistidine + Glucose <===> Protein histidine + alpha-D-Glucose 6-phosphate |
| ec:2.7.1.40 | Helo_4243 Helo_1605 | R00200 | ATP:pyruvate 2-O-phosphotransferase | Pyruvate + ATP <=== PEP + ADP |
| ec:2.3.1.12 | Helo_3571 Helo_2372 | R00209 | pyruvate dehydrogenase complex | Pyruvate + CoA + NAD <===> CO2 + Acetyl-CoA + NADH + H+ |
| ec:4.1.2.13 | Helo_1183 Helo_1180 | R01070 | beta-D-fructose-1,6-bisphosphate D-glyceraldehyde-3-phosphate-lyase (glycerone-phosphate-forming) | beta-D-Fructose 1,6-bisphosphate <===> Glyceraldehyde 3-phosphate + Glycerone phosphate |
| ec:1.8.1.4 | Helo_3110 | R08549 | 2-Oxoglutarate dehydrogenase complex | CoA + NAD + 2-Oxoglutarate <===> CO2 + NADH + H+ + Succinyl-CoA |
| ec:1.8.1.4 | Helo_3110 | R00209 | pyruvate dehydrogenase complex | Pyruvate + CoA + NAD <===> CO2 + Acetyl-CoA + NADH + H+ |
| ec:6.2.1.1 | Helo_2142 Helo_3563 | R00316 | ATP:acetate adenylyltransferase | ATP + Acetate <===> PPi + Acetyl adenylate |
| ec:6.2.1.1 | Helo_2142 Helo_3563 | R00236 | acetyl adenylate:CoA acetyltransferase | CoA + Acetyl adenylate <===> Acetyl-CoA + AMP |
| ec:1.2.1.12 | Helo_4242 Helo_1182 Helo_2131 Helo_2214 | R01061 | D-glyceraldehyde-3-phosphate:NAD+ oxidoreductase (phosphorylating) | NAD + Glyceraldehyde 3-phosphate + Phosphate <===> NADH + 1,3-Bisphospho-D-glycerate + H+ |
| ec:2.7.2.3 | Helo_1181 | R01512 | ATP:3-phospho-D-glycerate 1-phosphotransferase | ATP + 3-Phosphoglycerate <===> 1,3-Bisphospho-D-glycerate + ADP |
| ec:1.1.1.27 | Helo_1046 | R00703 | (S)-Lactate:NAD+ oxidoreductase | NAD + L-Lactate <===> Pyruvate + NADH + H+ |
| ec:2.7.1.11 | Helo_2186 | R04779 | ATP:D-fructose-6-phosphate 1-phosphotransferase | beta-D-Fructose 6-phosphate + ATP ===> beta-D-Fructose 1,6-bisphosphate + ADP |

Other reactions

| ec:2.7.3.9 | | Helo_1696 Helo_3698 Helo_3100 | R02628 | Phosphoenolpyruvate:protein-L-histidine N-pros-phosphotransferase | PEP + Protein histidine ===> Pyruvate + Protein N-pros-phosphohistidine |
| --- | --- | --- | --- | --- | --- |
| ec:2.6.1.1 | | Helo_2764 Helo_2013 Helo_4120 | R00355 | L-Aspartate:2-oxoglutarate aminotransferase | L-Aspartate + 2-Oxoglutarate <===> Glutamate + Oxaloacetate |
| ec:1.4.1.2 | | Helo_3049 | R00243 | L-Glutamate:NAD+ oxidoreductase (deaminating) | Glutamate + H2O + NAD <===> NH3 + NADH + H+ + 2-Oxoglutarate |
| ec:4.1.3.1 | | Helo_3070 | R00479 | isocitrate glyoxylate-lyase (succinate-forming) | Isocitrate <===> Glyoxylate + Succinate |
| ec:2.7.4.3 | | Helo_3570 | R00127 | ATP:AMP phosphotransferase | AMP + ATP <===> 2.0 ADP |
|  | Helo_3664 | |  | DoeB | H2O + N-alpha-Acetyldiaminobutyrate ===> L-2,4-Diaminobutyrate + Acetate |
|  | Helo_3665 | |  | DoeA | Ectoine + H2O <===> N-alpha-Acetyldiaminobutyrate |
|  |  | |  | ATP load | H2O + ATP ===> Phosphate + ADP |
|  |  | |  | NADH NADPH conversion | NAD + NADPH <===> NADH + NADP |

**Oxidative Phosphorylation**

|  |  |  | NADH oxidation | NADH + O2 + H+ + 1.7 Phosphate + 1.7 ADP ===> 1.7 H2O + NAD + 1.7 ATP |
| --- | --- | --- | --- | --- |
|  |  |  | FADH oxidation | O2 + AH2 + H+ + 1.1 Phosphate + 1.1 ADP ===> 1.1 H2O + 1.1 ATP + A |

**Table S5.** Bacterial strains and plasmids used in this study.

| Strain or plasmid | Relevant genotype and/or description*^a^* | Source or reference |
| --- | --- | --- |
| *H. elongata* |  |  |
| DSM 2581^T^ | type strain | DSMZ*^b^* |
| KB1 | Δ*ectA* | (Grammann et al., 2002) |
| KB41 | Δ*doeA* | This study |
| KB42 | Δ*doeB* | This study |
| KB47 | Δ*doeC* | This study |
| KB48 | Δ*doeD* | This study |
| KB49 | Δ*eutB* | This study |
| KB50 | Δ*eutC* | This study |
| SB1 | Δ*ectB* | This study |
| SB1.1 | Δ*ectB,* Δ*doeD* | This study |
| KB2.11 | Δ*teaABC* | This study |
| KB2.13 | Δ*teaABC*, Δ*doeA* | This study |
| *E. coli* |  |  |
| BL21 | F^-^ *ompT hsdS*_B_ (r_B_^-^m_B_^-^) *gal dcm rne131* (DE3) | Invitrogen |
| DH5α | F^-^ φ*80dlacZDM15* Δ(*lacZYA-argF*) *U169 recA1 hsdR17* (*rK^-^ mK^+^*) *supE44* Δ^-^  *thi-1 gyrA relA1* | (Hanahan, 1983) |
| S17-1 | *thi pro hsdR^-^ hsdM*^+^ *recA*; Tp^r^, Sm^r^ | (Simon et al., 1983) |
| Plasmids |  |  |
| pK18*mobsacB* | Km^r^ , *mob,* *sacB* | (Schäfer et al., 1994) |
| pJB3Cm6 | Cm^r^ | (Blatny et al., 1997) |
| pKSB7 | pJB3Cm6::*doeA;* Cm^r^ | This study |
| pJSB3 | pJB3Cm6::*doeB;* Cm^r^ | This study |
| pET101 | Ap^r^ | Invitrogen |
| pKSB11 | pET101::*doeA*, Ap^r^ | This study |

*^a^* Abbreviations of antibiotics: Cm, chloramphenicol; Km, kanamycin; Ap, ampicillin; Sm, streptomycin; Tp, trimethoprim

*^b^* DSMZ: Deutsche Sammlung von Mikroorganismen und Zellkulturen, Braunschweig, Germany

**Table S6.** Locus tag of genes similar to *ect* genes, *ask*, and *doe* genes depicted in Figure 2, Figure 4, and Figure 7, and accession data of genome sequences of corresponding organisms.

| **Organism and Accession Number Genome Sequence** | **Gene Name** | Locus Tag |
| --- | --- | --- |
| *Acidiphilium cryptum* JF-5  CP000697 | *ectABCD, ask* | Acry_3008, 3009, 3010, 3011, 3012 |
| *Agrobacterium tumefaciens* C58  AE007870 | *doeCD*  *eutBC*  *doeAB* | Atu4762, 4761  Atu4759, 4758  Atu4757, 4756 |
| *Alkalilimnicola ehrlichii* MLHE-1  CP000453 | *ectC*  *ectAB*  *ectD* | Mlg_1190  Mlg_1192, 1191  Mlg_0392 |
| *Alkanivorax borkumensis* SK2  AM286690 | *ectABC*  *ectD* | ABO_2150, 2151, 2152  ABO_0023 |
| *Bacillus clausii* KSM-K16  AP006627 | *ectABC*  *ectD* | ABC0334, 0335, 0336  ABC3489 |
| *Bacillus halodurans* C-125  BA000004 | *ectABC* | BH0920, 0919, 0918 |
| *Blastospirellula marina* DSM 3645  AANZ00000000 | *ectABCD* | DSM3645_02398, 02393, 02388, 02383 |
| *Bordetella bronchiseptica* RB50  BX470250 | *ectABCD* | BB3220, 3219, 3218, 3217 |
| *Bordetella parapertussis* | *ectABCD* | BPP1888, 1889, 1890, 1891 |
| *Bordetella petrii* DSM 1280  AM902716 | *ectABCD* | Bpet1981, 1982, 1983, 1984 |
| *Burkholderia cenocepacia* AU1054  CP000380 | *doeABXCD* | Bcen_5803, 5802, 5801, 5800, 5799 |
| *Burkholderia cenocepacia* HI2424  CP000460 | *doeABXCD* | Bcen*2424*_6167, 6166, 6165, 6164, 6163 |
| *Burkholderia phymatum* STM815  CP001044 | *doeABXCD* | Bphy_3862, 3861, 3860, 3859, 3858 |
| *Burkholderia vietnamiensis* G4  CP000616 | *doeABXCD* | Bcep*1808*_5462, 5461, 5460, 5459, 5458 |
| *Burkholderia xenovorans* LB400  CP000272 | *doeA*  *doeBXCD* | Bxe_C0063  Bxe_C0058, C0059, C0060, C0061 |
| *Chromohalobacter salexigens* DSM 3043^T^  CP000285 | *ectABC,*  *ectD*  *ectE*  *doeABX*  *doeCD* | Csal_1875, 1876, 1877  Csal_0542  Csal_3003  Csal_2732, 2731, 2730  Csal_2724, 2723 |
| *Geobacillus thermodenitrificans* NG80-2  CP000557 | *doeA* | GTNG_2272 |
| *Hahella chejuensis* KCTC 2396  CP000155 | *ectABCD*  *doeA* | HCH_01510, 01509, 01508, 01507  HCH_06289 |
| *Haloferax volcanii* DS2  CP001953 | *doeA* | HVO_B0267 |
| *Halomonas elongata* DSM 2581^T^  FN869568 | *ectABC*  *ectD*  *doeABXCD* | Helo_2588, 2589, 2590  Helo_4008  Helo_3665, 3664, 3663, 3662, 3661 |
| *Halorhodospira halophila* SL1  CP000544 | *ectABC* | Hhal_1732, 1733, 1734 |
| *Halorubrum lacusprofundi* ATCC 49239  CP001365 | *doeA* | Hlac_1051 |
| *Herminiimonas arsenicoxydans* ULPAs1  CU207211 | *ectABCD* | HEAR3380, 3379, 3378, 3377 |
| *Hyphomonas neptunium* ATCC 15444  CP000158 | *ectABCD, ask* | HNE_1639, 1640, 1641, 1642, 1643 |
| *Jannaschia* sp.CCS1  CP000264 | *doeABD* | Jann_0849, 0850, 0851 |
| *Janthinobacterium* sp. Marseille  CP000269 | *ectABCD* | mma_3601, 3600, 36599, 36598 |
| *Marinobacter hydrocarbonoclasticus* DSM 11845  CP000514 | *ectAB, ask,*  *ectC, ectC, ectC,*  *ectD, ectD* | Maqu_0147, 0148, 0149  Maqu_0079, 0616, 0444  Maqu_1849, 3892 |
| *Marinomonas* sp. MWYL1  CP000749 | *ectABCD, ask* | Mmwyl1_1158, 1159, 1160, 1161, 1162 |
| *Mesorhizobium loti* MAFF303099  BA000012 | *doeDCX,*  *eutBC*  *doeAB* | mll7127, mll7178, mll7129  mlr7138, mlr 7139  mlr7141. mlr7142 |
| *Methylophaga thiooxidans* MDS010  ABXT00000000 | *ectABC, ask* | MDMS009_1763, 2070, 2081, 1862 |
| *Mycobacterium gilvum* PYR-GCK  CP000656 | *ectABCD* | Mflv_4832, 4833, 4834, 4835 |
| *Mycobacterium* sp. JLS  CP000580 | *ectABCD* | Mjls_4417, 4418, 4419, 4420 |
| *Mycobacterium* sp. MCS  CP000384 | *ectABCD* | Mmcs_4190, 4191, 4192, 4193 |
| *Mycobacterium vanbaalenii* PYR-1  CP000511 | *ectABCD* | Mvan_5274, 5273, 5272, 5271 |
| *Natrialba magadii* ATCC 43099  CP001933 | *doeA* | Nmag_3760 |
| *Nitrosococcus oceani* ATCC 19707  CP000127 | *ectABD*  *ectC, ask* | Noc_1562, 1561, 1560  Noc_1028, Noc_1029 |
| *Nitrosopumilus maritimus* SCM1  CP000866 | *ectABCD* | Nmar_1346, 1345, 1344, 1343 |
| *Norcadia farcinica* IFM10152  AP006618 | *ectABCD* | nfa27160, 27170, 27180, 27190 |
| *Oceanobacillus iheyensis* HTE831  BA000028 | *ectBC*  *doeA* | OB0518, 0519  OB2950 |
| *Ochrobactum anthropi* ATCC 49188  CP000759 | *doeXCD*  *eutBC*  *doeAB* | Oant_3470, 3471, 3472  Oant_3469, 3468  Oant_3467, 3466 |
| *Phenylobacterium zucineum HLK1*  CP000747 | *ectABCD, ask* | PHZ_c1335, c1336, c1337, c1338, c1339 |
| *Pseudomonas aeruginosa* PA7  CP000744 | *doeABXCD* | PSPA7_4378, 4379, 4380 4381, 4382 |
| *Pseudomonas stutzeri* A1501  CP000304 | *ectABCD, ask* | PST_0181, 0180, 0179. 0178, 0177 |
| *Rhizobium etli* CFN42  CP000138 | *doeXCD*  *eutBC*  *doeAB* | RHE_PF00200, PF00201, PF202  RHE_PF00182, 00181  RHE_PF00180, PF00179 |
| *Rhizobium leguminosarum* bv. *viciae* 3841  AM236086 | *doeDCX*  *eutBC*  *doeAB* | pRL120045, 120044, 120043  pRL120052, 120053  pRL120054, 120055 |
| *Roseobacter denitrificans* OCh 114  CP000362 | *doeAB* | RD1_3474, 3475 |
| *Ruegeria* *pomeroyi* DSS-3  CP000031 | *doeABXCD* | SPO1140, 1139, 1138, 1137, 1136 |
| *Ruegeria* sp. TM1040  CP000377 | *ectABC*  *ask*  *doeA*  *doeBXCD* | TM*1040*_0550, 0551, 0552, 0553, TM*1040*_0554  TM*1040*_1921  TM*1040*_2694, 2693, 2692, 2691 |
| *Saccharophagus degradans* 2-40  CP000282 | *ectABC, ask*  *ectD* | SDE_1189, 1190, 1191  Sde_1259 |
| *Sinorhizobium meliloti* 1021  AL591985 | *doeXCD*  *eutBC*  *doeAB* | SM_b20425, b20424, b20423  SM_b20432, b20433  SM_b20434, b20435 |
| *Sphingopyxis alaskensis* RB 2256  Sala_2949 | *ectABCD, ask* | Sala_2949, 2950, 2951, 2952, 2953 |
| *Streptomyces avermitilis* MA-4680  BA000030 | *ectABCD* | SAV_6398, 6397, 6396, 6395 |
| *Streptomyces coelicolor* A3  AL645882 | *ectABCD* | SCO1864, 1865, 1866, 1867 |
| *Thermobifida fusca* YX  CP000088 | *ectABC* | TFU_0300, 0301, 0302 |
| *Thiomicrospira crunogena* XCL-2  CP000109 | *ectABC, ask*  *doeA* | Tcr_0518, 0519, 0520, 0521  Tcr_0221 |
| *Verminephrobacter eiseniae* EF01-2  CP000542 | *doeABXCD* | Veis_2149, 2148, 2147, 2146, 2145 |
| *Vibrio cholerae* O395  CP000626 | *ectABC, ask* | VC*O395*_0409, 0410, 0411, 0412 |
| *Vibrio fischeri* ATCC 700601  CP000021 | *ectABC, ask* | VF_A1122, A1123, A1124, A1125 |
| *Vibrio harveyi* ATCC BAA-1116  CP000789 | *ectABC, ask* | VIBHAR_02454, 02453, 02453, 02451 |
| *Vibrio parahaemolyticus* RIMD 2210633  BA000031 | *ectABC, ask* | VP1722, 1721, 1720, 1719 |
| *Vibrio* *splendidus* LGP32  FM954973 | *ectABC, ask* | VS_II0066, II0067, II0068, II0069 |
| *Wolinella succinogenes* DSM 1740  BX571656 | *ectABC* | WS0854, 0855, 0856 |
