# Supplementary material for: A blueprint of ectoine metabolism from the genome of the industrial producer Halomonas elongata DSM 2581T
Source: Environ Microbiol. 2011 Aug;13(8):1973–94. doi: 10.1111/j.1462-2920.2010.02336.x (PMC3187862; doi:10.1111/j.1462-2920.2010.02336.x)
Supplement: Supplementary file 8 [file emi0013-1973-SD8.doc]

### Supporting Information

**Figure S1.** Species-level assignment of *H. elongata* proteins by MEGAN analysis. The plot indicates the number of proteins assigned to the named species out of a set of 1,672 proteins for which such an assignment was successful. The number of assigned proteins is given in parenthesis after the name of the species. Only 8% of the proteins are not assigned to *C. salexigens*.

**Figure S2.** Alignment of the *H. elongata* and *C. salexigens* chromosomes. The chromosomes of *H. elongata* and *C. salexigens* were aligned using MUMmer software (Kurtz et al., 2004) and show a strong X-alignment. Such X-alignments have been described for several interspecies comparisons and attributed to genome inversions around the replication origin (Eisen et al., 2000). A prominent X-alignment probably indicates that the number of such genome rearrangement events was small, which is astonishing for species with such an evolutionary distance that they are classified into distinct genera.

**Figure S3.** Utilization of acetate as carbon source by *H. elongata* strains in the absence and presence of ectoine. *H. elongata* wild type, mutant strains KB41 (Δ*doeA*), KB42 (Δ*doeB*), and KB47 (Δ*doeD*) were incubated for three days at 30° C on mineral salt medium (0.51 M NaCl) containing 40 mM acetate (A) and 40 mM acetate plus 10 mM ectoine (B) respectively. All strains were able to grow on acetate as sole carbon source (A). In the presence of ectoine, mutant KB47 failes to grow with acetate, while KB41 and KB42 are still able to utilize acetate (B). The inability of *doeD* mutant KB47 to grow on acetate in the presence of ectoine explains why KB47 also fails to grow with ectoine alone although acetate should be still provided due to the deacetylase activity of DoeB (Figure 3).

**Figure S4.** RT-PCR and RACE-PCR analysis of the *doeABXC* region. (A) Genetic and physical organization of the *doeABX* locus. The position of the σ^70^-dependent promoter is indicated. Primer binding-sites for RT-PCR are marked by triangles. Black triangles with dotted lines show successful generation of PCR products. For the pair of white triangles, no PCR product was obtained. Reverse primers were used for both, RT reaction and PCR. (B) RT-PCR analysis proving that *doeABX* is organized as one operon. A 1450 bp PCR product was amplified from cDNA and separated by agarose-gel electrophoresis (lane 1), which matched the size of the calculated *doeABX* PCR product (1473 bp). A corresponding *doeX-doeC* product could be amplified from genomic DNA with the same primer pair (positive control, lane 2). No PCR product could be amplified from cDNA using a primer pair to *doeX* and *doeC,* proving that *doeC* is not part of the *doeABX* operon (lane 3). A corresponding *doeX-doeC* product could be amplified from genomic DNA with the same primer pair (positive control, lane 4). A PCR product could be amplified with primers that both bind within the *doeC* ORF (control RT reaction, data not shown, product indicated in A). (C) Nucleotide sequence of the *doeA* promoter region. Arrows indicate the transcription initiation-site (+1), which was mapped by RACE-PCR. The –35 and –10 sequences of the σ^70^-dependent promoter upstream of *doeA* are written in bold.

**Figure S5. Maximum ectoine yield as a function of the ATP load and turnover of ectoine in the synthesis degradation cycle (A) and flux distributions for maximum conversion into ectoine (B).** (A) Points I to IV mark the flux distributions described in panel B. Horizontal axes represent total consumption of ATP by processes outside the model and the flux circulating through the ectoine synthesis/degradation cycle. The units are arbitrary, normalized for a glucose uptake of 100 (e.g. molecules). (B) I) One of the admissible flux distributions without ATP demand. II) Unique solution when total ATP demand is less than or equal to one ATP per glucose III) Example of solutions for higher ATP demands, superposition of II and maximum ATP production distribution. IV) Example of solutions for a turnover of ectoine, identical to III everywhere except the ATP load and cycle.

**Table S1.** The 20 COGs with the highest occupancy.

Shown are the numbers of proteins in *H. elongata* [#(H.elo)] and in *C. salexigens* [#(C.sal)], as well as the number of bidirectional best blast pairs. All of the 20 highest occupancy COGs belong to the four categories “transcription regulators”, “broad-specificity enzymes”, “transporters and their subunits” and “two-component systems”. A high fraction of the COG members are ortholog pairs in *H. elongata* and *C. salexigens*. Commonly, the number of COG members is similar in the two organisms.

| COG | category | #(H.elo) | #(C.sal) | pairs | COG description |
| --- | --- | --- | --- | --- | --- |
| COG0583 | regulator | 57 | 52 | 36 | Transcriptional regulator |
| COG1028 | enzyme | 26 | 29 | 14 | Dehydrogenases with different specificities (related to short-chain alcohol dehydrogenases) |
| COG0477 | transporter | 25 | 22 | 15 | Permeases of the major facilitator superfamily |
| COG1638 | transporter | 23 | 18 | 13 | TRAP-type C4-dicarboxylate transport system, periplasmic component |
| COG1012 | enzyme | 22 | 20 | 12 | NAD-dependent aldehyde dehydrogenases |
| COG0642 | two-comp. | 21 | 17 | 14 | Signal transduction histidine kinase |
| COG1593 | transporter | 21 | 15 | 11 | TRAP-type C4-dicarboxylate transport system, large permease component |
| COG1309 | regulator | 16 | 10 | 9 | Transcriptional regulator |
| COG0745 | two-comp. | 14 | 11 | 9 | Response regulators consisting of a CheY-like receiver domain and a winged-helix DNA-binding domain |
| COG0454 | enzyme | 14 | 10 | 10 | Histone acetyltransferase HPA2 and related acetyltransferases |
| COG0697 | transporter | 12 | 11 | 7 | Permeases of the drug/metabolite transporter (DMT) superfamily |
| COG2207 | regulator | 12 | 11 | 8 | AraC-type DNA-binding domain-containing proteins |
| COG0500 | enzyme | 12 | 9 | 8 | SAM-dependent methyltransferases |
| COG4977 | regulator | 11 | 5 | 4 | Transcriptional regulator containing an amidase domain and an AraC-type DNA-binding HTH domain |
| COG0665 | enzyme | 10 | 10 | 9 | Glycine/D-amino acid oxidases (deaminating) |
| COG1802 | regulator | 10 | 10 | 7 | Transcriptional regulators |
| COG3090 | transporter | 10 | 9 | 6 | TRAP-type C4-dicarboxylate transport system, small permease component |
| COG1396 | regulator | 10 | 7 | 5 | Predicted transcriptional regulators |
| COG3839 | transporter | 10 | 6 | 5 | ABC-type sugar transport systems, ATPase components |
| COG0609 | transporter | 9 | 7 | 6 | ABC-type Fe3+-siderophore transport system, permease component |

**Table S2.** Organisms used for the search for high salt COGs.

The halophilic (H), marine (M), and standard (S) organisms used in the genome comparison analysis are listed.

| taxonomic branch | halophilic/marine | H/M/S | TaxID | standard organism |
| --- | --- | --- | --- | --- |
| [Firmicutes](http://www.ncbi.nlm.nih.gov/Taxonomy/Browser/wwwtax.cgi?mode=Info&id=1239&lvl=6&lin=f&keep=1&srchmode=5&unlock&filter=genome_filter) | *Bacillus halodurans* C-125 | H | 272558 |  |
| [Firmicutes](http://www.ncbi.nlm.nih.gov/Taxonomy/Browser/wwwtax.cgi?mode=Info&id=1239&lvl=6&lin=f&keep=1&srchmode=5&unlock&filter=genome_filter) | *Oceanobacillus iheyensis* HTE831 | M | 221109 |  |
| [Firmicutes](http://www.ncbi.nlm.nih.gov/Taxonomy/Browser/wwwtax.cgi?mode=Info&id=1239&lvl=6&lin=f&keep=1&srchmode=5&unlock&filter=genome_filter) |  | S | 224308 | *Bacillus subtilis* subsp. *subtilis* str. 168 |
| [Firmicutes](http://www.ncbi.nlm.nih.gov/Taxonomy/Browser/wwwtax.cgi?mode=Info&id=1239&lvl=6&lin=f&keep=1&srchmode=5&unlock&filter=genome_filter) |  | S | 222523 | *Bacillus cereus* ATCC 10987 |
| [Bacteroidetes](http://www.ncbi.nlm.nih.gov/Taxonomy/Browser/wwwtax.cgi?mode=Info&id=68336&lvl=6&lin=f&keep=1&srchmode=5&unlock&filter=genome_filter) | *Salinibacter ruber* DSM 13855 | H | 309807 |  |
| [Gamma-proteobacteria](http://www.ncbi.nlm.nih.gov/Taxonomy/Browser/wwwtax.cgi?mode=Info&id=1224&lvl=6&lin=f&keep=1&srchmode=5&unlock&filter=genome_filter) | *Chromohalobacter salexigens* DSM 3043 | H | 290398 |  |
| [Gamma-proteobacteria](http://www.ncbi.nlm.nih.gov/Taxonomy/Browser/wwwtax.cgi?mode=Info&id=1224&lvl=6&lin=f&keep=1&srchmode=5&unlock&filter=genome_filter) | *Hahella chejuensis* KCTC 2396 | M | 349521 |  |
| [Gamma-proteobacteria](http://www.ncbi.nlm.nih.gov/Taxonomy/Browser/wwwtax.cgi?mode=Info&id=1224&lvl=6&lin=f&keep=1&srchmode=5&unlock&filter=genome_filter) | *Marinobacter aquaeolei* VT8 | M | 351348 |  |
| [Gamma-proteobacteria](http://www.ncbi.nlm.nih.gov/Taxonomy/Browser/wwwtax.cgi?mode=Info&id=1224&lvl=6&lin=f&keep=1&srchmode=5&unlock&filter=genome_filter) | *Marinomonas sp*. MWYL1 | M | 400668 |  |
| [Gamma-proteobacteria](http://www.ncbi.nlm.nih.gov/Taxonomy/Browser/wwwtax.cgi?mode=Info&id=1224&lvl=6&lin=f&keep=1&srchmode=5&unlock&filter=genome_filter) | *Shewanella denitrificans* OS217 | M | 318161 |  |
| [Gamma-proteobacteria](http://www.ncbi.nlm.nih.gov/Taxonomy/Browser/wwwtax.cgi?mode=Info&id=1224&lvl=6&lin=f&keep=1&srchmode=5&unlock&filter=genome_filter) | *Vibrio fischeri* ES114 | M | 312309 |  |
| [Gamma-proteobacteria](http://www.ncbi.nlm.nih.gov/Taxonomy/Browser/wwwtax.cgi?mode=Info&id=1224&lvl=6&lin=f&keep=1&srchmode=5&unlock&filter=genome_filter) | *Nitrosococcus oceani* ATCC 19707 | M | 323261 |  |
| [Gamma-proteobacteria](http://www.ncbi.nlm.nih.gov/Taxonomy/Browser/wwwtax.cgi?mode=Info&id=1224&lvl=6&lin=f&keep=1&srchmode=5&unlock&filter=genome_filter) |  | S | 220664 | *Pseudomonas fluorescens* Pf-5 |
| [Gamma-proteobacteria](http://www.ncbi.nlm.nih.gov/Taxonomy/Browser/wwwtax.cgi?mode=Info&id=1224&lvl=6&lin=f&keep=1&srchmode=5&unlock&filter=genome_filter) |  | S | 83333 | *Escherichia coli* K12 |
| [Gamma-proteobacteria](http://www.ncbi.nlm.nih.gov/Taxonomy/Browser/wwwtax.cgi?mode=Info&id=1224&lvl=6&lin=f&keep=1&srchmode=5&unlock&filter=genome_filter) |  | S | 399741 | *Serratia proteamaculans* 568 |
| [Gamma-proteobacteria](http://www.ncbi.nlm.nih.gov/Taxonomy/Browser/wwwtax.cgi?mode=Info&id=1224&lvl=6&lin=f&keep=1&srchmode=5&unlock&filter=genome_filter) |  | S | 297246 | *Legionella pneumophila* str. Paris |
| Alpha-p[roteobacteria](http://www.ncbi.nlm.nih.gov/Taxonomy/Browser/wwwtax.cgi?mode=Info&id=1224&lvl=6&lin=f&keep=1&srchmode=5&unlock&filter=genome_filter) | *Silicibacter sp.* TM1040 | M | 292414 |  |
| Alpha-p[roteobacteria](http://www.ncbi.nlm.nih.gov/Taxonomy/Browser/wwwtax.cgi?mode=Info&id=1224&lvl=6&lin=f&keep=1&srchmode=5&unlock&filter=genome_filter) | *Roseobacter denitrificans* OCh 114 | M | 375451 |  |
| Alpha-p[roteobacteria](http://www.ncbi.nlm.nih.gov/Taxonomy/Browser/wwwtax.cgi?mode=Info&id=1224&lvl=6&lin=f&keep=1&srchmode=5&unlock&filter=genome_filter) |  | S | 176299 | *Agrobacterium tumefaciens* str. C58 |
| Alpha-p[roteobacteria](http://www.ncbi.nlm.nih.gov/Taxonomy/Browser/wwwtax.cgi?mode=Info&id=1224&lvl=6&lin=f&keep=1&srchmode=5&unlock&filter=genome_filter) |  | S | 264203 | *Zymomonas mobilis* subsp. *mobilis* ZM4 |
| Alpha-p[roteobacteria](http://www.ncbi.nlm.nih.gov/Taxonomy/Browser/wwwtax.cgi?mode=Info&id=1224&lvl=6&lin=f&keep=1&srchmode=5&unlock&filter=genome_filter) |  | S | 269796 | *Rhodospirillum rubrum* ATCC 11170 |
| Alpha-p[roteobacteria](http://www.ncbi.nlm.nih.gov/Taxonomy/Browser/wwwtax.cgi?mode=Info&id=1224&lvl=6&lin=f&keep=1&srchmode=5&unlock&filter=genome_filter) |  | S | 439375 | *Ochrobactrum anthropi* ATCC 49188 |
| [Beta-proteobacteria](http://www.ncbi.nlm.nih.gov/Taxonomy/Browser/wwwtax.cgi?mode=Info&id=1224&lvl=6&lin=f&keep=1&srchmode=5&unlock&filter=genome_filter) |  | S | 375286 | *Janthinobacterium* sp. Marseille |
| [Beta-proteobacteria](http://www.ncbi.nlm.nih.gov/Taxonomy/Browser/wwwtax.cgi?mode=Info&id=1224&lvl=6&lin=f&keep=1&srchmode=5&unlock&filter=genome_filter) |  | S | 228410 | *Nitrosomonas europaea* ATCC 19718 |
| [Cyanobacteria](http://www.ncbi.nlm.nih.gov/Taxonomy/Browser/wwwtax.cgi?mode=Info&id=1117&lvl=3&lin=f&keep=1&srchmode=5&unlock&filter=genome_filter) | *Prochlorococcus marinus* str. MIT 9313 | M | 74547 |  |
| [Cyanobacteria](http://www.ncbi.nlm.nih.gov/Taxonomy/Browser/wwwtax.cgi?mode=Info&id=1117&lvl=3&lin=f&keep=1&srchmode=5&unlock&filter=genome_filter) |  | S | 103690 | *Nostoc sp*. PCC 7120 |
| [Aquificae](http://www.ncbi.nlm.nih.gov/Taxonomy/Browser/wwwtax.cgi?mode=Undef&id=200783&lvl=3&keep=1&srchmode=5&unlock&filter=genome_filter) |  | S | 224324 | *Aquifex aeolicus* VF5 |

### Table S3. Average pI difference between proteins from *H. elongata* and the indicated organisms.

The pI differences were computed for the 27 halophilic (H), marine (M), and standard (S) organisms listed in Table S2. Protein pair selection is based on COG assignments. Each COG that contains a single protein in *H. elongata* and in the indicated organism was included in the analysis. The number of included COGs (and thus protein pairs) is indicated. The pI of the two proteins and their difference was computed. The pI differences were averaged (pI shift). Negative numbers indicate that the average pI of the compared organism is more acidic, positive number that it is more alkaline. The table is sorted by pI shift. It should be noted that most of the pI shifts are only minimal but that only two organisms have a shift towards a more acidic proteome.

| Organism | H/M/S | pI shift | # pairs |
| --- | --- | --- | --- |
| *Salinibacter ruber* | H | -0.7 | 524 |
| *Oceanobacillus iheyensis* | M | -0.1 | 561 |
| *Marinobacter aquaeolei* | M | 0.0 | 952 |
| *Silicibacter sp*. TM1040 | M | 0.0 | 659 |
| Chromohalobacter salexigens | H | 0.1 | 1114 |
| *Marinomonas sp.* | M | 0.1 | 924 |
| *Vibrio fischeri* | M | 0.1 | 730 |
| *Roseobacter denitrificans* | M | 0.1 | 714 |
| *Bacillus halodurans* | H | 0.1 | 615 |
| *Bacillus cereus* | S | 0.1 | 549 |
| *Hahella chejuensis* | M | 0.2 | 926 |
| *Shewanella denitrificans* | M | 0.2 | 832 |
| *Bacillus subtilis* | S | 0.2 | 594 |
| *Nostoc sp*. PCC 7120 | S | 0.2 | 529 |
| Escherichia coli | S | 0.4 | 855 |
| *Agrobacterium tumefaciens* | S | 0.4 | 608 |
| *Ochrobactrum anthropi* | S | 0.4 | 574 |
| *Pseudomonas fluorescens* | S | 0.5 | 920 |
| *Serratia proteamaculans* | S | 0.5 | 866 |
| *Rhodospirillum rubrum* | S | 0.5 | 679 |
| *Prochlorococcus marinus* | M | 0.5 | 517 |
| *Nitrosomonas europaea* | S | 0.6 | 674 |
| *Janthinobacterium* sp. Marseille | S | 0.7 | 752 |
| Zymomonas mobilis | S | 0.8 | 566 |
| *Nitrosococcus oceani* | M | 0.9 | 755 |
| *Legionella pneumophila* | S | 0.9 | 670 |
| *Aquifex aeolicus* | S | 1.3 | 508 |

### Table S4. Enzymatic reactions used in the metabolic models.

The table contains the reaction number, reaction name, and the catalyzed reaction (columns 3 to 5, data taken from KEGG). In the first two columns, the EC number and the annotated *H. elongata* protein(s) are indicated.

##### Glycine, Serine and Threonine metabolism

| ec:2.7.1.39 | Helo_4004 | R01771 | ATP:L-homoserine O-phosphotransferase | L-Homoserine + ATP ===> O-Phospho-L-homoserine + ADP |
| --- | --- | --- | --- | --- |
| ec:4.2.1.108 | Helo_2590 | R06979 | Ectoine hydro-lyase | N-gamma-Acetyldiaminobutyrate ===> Ectoine + H2O |
| ec:2.6.1.52 | Helo_2720 | R04173 | 3-Phosphoserine:2-oxoglutarate aminotransferase | Dexfosfoserine + 2-Oxoglutarate <===> Glutamate + 3Phosphohydroxypyruvate |
| ec:2.6.1.76 | Helo_2589 | R06977 | L-2,4-diaminobutyrate:2-oxoglutarate 4-aminotransferase | Glutamate + L-Aspartic 4-semialdehyde <===> L-2,4-Diaminobutyrate + 2-Oxoglutarate |
| ec:3.1.3.3 | Helo_1722 | R00582 | O-phospho-L-serine phosphohydrolase | Dexfosfoserine + H2O ===> Phosphate + Serine |
| ec:1.1.1.95 | Helo_1081 Helo_3667 | R01513 | 3-Phospho-D-glycerate:NAD+ 2-oxidoreductase | NAD + 3-Phosphoglycerate <===> 3Phosphohydroxypyruvate + NADH + H+ |
| ec:2.1.2.1 | Helo_3598 Helo_3449 | R00945 | 5,10-Methylenetetrahydrofolate:glycine hydroxymethyltransferase | Gly + H2O + 5,10-Methylene-THF <===> THF + Serine |
| ec:4.2.3.1 | Helo_3856 | R01466 | L-threonine-forming) | H2O + O-Phospho-L-homoserine <===> L-Threonine + Phosphate |
| ec:1.1.1.3 | Helo_3857 | R01773 | L-Homoserine:NAD+ oxidoreductase | L-Homoserine + NAD <===> L-Aspartic 4-semialdehyde + NADH + H+ |
| ec:2.1.2.10 | Helo_2529 Helo_1668 Helo_2544 | R04125 | S-aminomethyl-dihydrolipoylprotein:(6S)-tetrahydrofolate aminomethyltransferase (ammonia-forming) | THF + S-Aminomethyldihydrolipoylprotein <===> NH3 + Dihydrolipoylprotein + 5,10-Methylene-THF |
| ec:4.3.1.17 | Helo_3468 | R00220 | L-serine ammonia-lyase | Serine ===> NH3 + Pyruvate |
| ec:1.8.1.4 | Helo_3110 | R08549 | 2-Oxoglutarate dehydrogenase complex | CoA + NAD + 2-Oxoglutarate <===> CO2 + NADH + H+ + Succinyl-CoA |
| ec:1.8.1.4 | Helo_3110 | R00209 | pyruvate dehydrogenase complex | Pyruvate + CoA + NAD <===> CO2 + Acetyl-CoA + NADH + H+ |
| ec:2.3.1.178 | Helo_2588 | R06978 | L-2,4-diaminobutyrate acetyltransferase | L-2,4-Diaminobutyrate + Acetyl-CoA ===> CoA + N-gamma-Acetyldiaminobutyrate |
| ec:2.7.2.4 | Helo_3742 | R00480 | ATP:L-aspartate 4-phosphotransferase | L-Aspartate + ATP ===> 4-Phospho-L-aspartate + ADP |
| ec:1.2.1.11 | Helo_2235 | R02291 | L-Aspartate-4-semialdehyde:NADP+ oxidoreductase (phosphorylating) | L-Aspartic 4-semialdehyde + NADP + Phosphate <===> 4-Phospho-L-aspartate + NADPH + H+ |
| ec:4.3.1.19 | Helo_1021 Helo_3660 Helo_3251 | R00220 | L-serine ammonia-lyase | Serine ===> NH3 + Pyruvate |

TCA cycle

| ec:2.3.3.1 | Helo_3117 | R00351 | acetyl-CoA:oxaloacetate C-acetyltransferase (thioester-hydrolysing) | CoA + Citrate <=== Acetyl-CoA + H2O + Oxaloacetate |
| --- | --- | --- | --- | --- |
| ec:4.1.1.49 | Helo_1685 | R00341 | phosphoenolpyruvate-forming) | ATP + Oxaloacetate ===> PEP + CO2 + ADP |
| ec:4.2.1.3 | Helo_2076 Helo_2437 Helo_3816 | R01900 | isocitrate hydro-lyase (cis-aconitate-forming) | Isocitrate <===> H2O + cis-Aconitate |
| ec:4.2.1.3 | Helo_2076 Helo_2437 Helo_3816 | R01325 | citrate hydro-lyase (cis-aconitate-forming) | Citrate <===> H2O + cis-Aconitate |
| ec:4.2.1.2 | Helo_2298 Helo_2547 | R01082 | (S)-malate hydro-lyase (fumarate-forming) | Malate <===> H2O + Fumarate |
| ec:1.2.4.2 | Helo_4075 Helo_3112 | R00621 | rn:R00621 | TPP + 2-Oxoglutarate <===> CO2 + 3-Carboxy-1-hydroxypropyl-ThPP |
| ec:1.2.4.2 | Helo_4075 Helo_3112 | R08549 | 2-Oxoglutarate dehydrogenase complex | CoA + NAD + 2-Oxoglutarate <===> CO2 + NADH + H+ + Succinyl-CoA |
| ec:1.2.4.1 | Helo_3572 | R00209 | pyruvate dehydrogenase complex | Pyruvate + CoA + NAD <===> CO2 + Acetyl-CoA + NADH + H+ |
| ec:1.3.99.1 | Helo_3114 Helo_3113 Helo_3115 Helo_3116 | R00412 | succinate:acceptor oxidoreductase | Succinate + A <===> AH2 + Fumarate |
| ec:1.1.1.42 | Helo_3063 Helo_3252 | R00268 | oxalosuccinate carboxy-lyase (2-oxoglutarate-forming) | Oxalosuccinate <===> CO2 + 2-Oxoglutarate |
| ec:1.1.1.42 | Helo_3063 Helo_3252 | R01899 | Isocitrate:NADP+ oxidoreductase | Isocitrate + NADP <===> Oxalosuccinate + NADPH + H+ |
| ec:2.3.1.12 | Helo_3571 Helo_2372 | R00209 | pyruvate dehydrogenase complex | Pyruvate + CoA + NAD <===> CO2 + Acetyl-CoA + NADH + H+ |
| ec:6.2.1.5 | Helo_2498 Helo_2634 Helo_3108 Helo_3109 | R00405 | Succinate:CoA ligase (ADP-forming) | CoA + ATP + Succinate <===> Phosphate + ADP + Succinyl-CoA |
| ec:1.8.1.4 | Helo_3110 | R08549 | 2-Oxoglutarate dehydrogenase complex | CoA + NAD + 2-Oxoglutarate <===> CO2 + NADH + H+ + Succinyl-CoA |
| ec:1.8.1.4 | Helo_3110 | R00209 | pyruvate dehydrogenase complex | Pyruvate + CoA + NAD <===> CO2 + Acetyl-CoA + NADH + H+ |
| ec:2.3.1.61 | Helo_3111 Helo_2296 | R08549 | 2-Oxoglutarate dehydrogenase complex | CoA + NAD + 2-Oxoglutarate <===> CO2 + NADH + H+ + Succinyl-CoA |
| ec:1.1.1.37 | Helo_3404 Helo_1693 | R00342 | (S)-malate:NAD+ oxidoreductase | NAD + Malate <===> NADH + H+ + Oxaloacetate |

Pyruvate metabolism

| ec:2.7.9.2 | Helo_2433 | R00199 | ATP:pyruvate,water phosphotransferase | Pyruvate + H2O + ATP ===> PEP + AMP + Phosphate |
| --- | --- | --- | --- | --- |
| ec:4.1.1.49 | Helo_1685 | R00341 | phosphoenolpyruvate-forming) | ATP + Oxaloacetate ===> PEP + CO2 + ADP |
| ec:4.1.1.3 | Helo_3734 Helo_3736 Helo_3735 | R00217 | oxaloacetate carboxy-lyase (pyruvate-forming) | Oxaloacetate ===> Pyruvate + CO2 |
| ec:2.3.3.9 | Helo_4288 | R00472 | L-Malate glyoxylate-lyase (CoA-acetylating) | CoA + Malate <=== Glyoxylate + Acetyl-CoA + H2O |
| ec:4.1.1.31 | Helo_3010 | R00345 | phosphoenolpyruvate-forming) | Phosphate + Oxaloacetate <=== PEP + CO2 + H2O |
| ec:1.2.4.1 | Helo_3572 | R00209 | pyruvate dehydrogenase complex | Pyruvate + CoA + NAD <===> CO2 + Acetyl-CoA + NADH + H+ |
| ec:1.2.1.3 | Helo_2817 | R00710 | Acetaldehyde:NAD+ oxidoreductase | Acetaldehyde + H2O + NAD <===> NADH + H+ + Acetate |
| ec:2.7.1.40 | Helo_4243 Helo_1605 | R00200 | ATP:pyruvate 2-O-phosphotransferase | Pyruvate + ATP <=== PEP + ADP |
| ec:2.3.1.12 | Helo_3571 Helo_2372 | R00209 | pyruvate dehydrogenase complex | Pyruvate + CoA + NAD <===> CO2 + Acetyl-CoA + NADH + H+ |
| ec:1.1.1.40 | Helo_3763 | R00217 | oxaloacetate carboxy-lyase (pyruvate-forming) | Oxaloacetate ===> Pyruvate + CO2 |
| ec:1.8.1.4 | Helo_3110 | R08549 | 2-Oxoglutarate dehydrogenase complex | CoA + NAD + 2-Oxoglutarate <===> CO2 + NADH + H+ + Succinyl-CoA |
| ec:1.8.1.4 | Helo_3110 | R00209 | pyruvate dehydrogenase complex | Pyruvate + CoA + NAD <===> CO2 + Acetyl-CoA + NADH + H+ |
| ec:6.2.1.1 | Helo_2142 Helo_3563 | R00316 | ATP:acetate adenylyltransferase | ATP + Acetate <===> PPi + Acetyl adenylate |
| ec:6.2.1.1 | Helo_2142 Helo_3563 | R00236 | acetyl adenylate:CoA acetyltransferase | CoA + Acetyl adenylate <===> Acetyl-CoA + AMP |
| ec:1.1.1.37 | Helo_3404 Helo_1693 | R00342 | (S)-malate:NAD+ oxidoreductase | NAD + Malate <===> NADH + H+ + Oxaloacetate |
| ec:1.1.1.38 | Helo_3817 | R00214 | (S)-malate:NAD+ oxidoreductase (decarboxylating) | NAD + Malate <===> Pyruvate + CO2 + NADH + H+ |
| ec:1.1.1.38 | Helo_3817 | R00217 | oxaloacetate carboxy-lyase (pyruvate-forming) | Oxaloacetate ===> Pyruvate + CO2 |
| ec:1.1.1.27 | Helo_1046 | R00703 | (S)-Lactate:NAD+ oxidoreductase | NAD + L-Lactate <===> Pyruvate + NADH + H+ |

Glycolysis and Gluconeogenesis

| ec:5.3.1.9 | Helo_1718 Helo_4245 | R02739 | alpha-D-Glucose 6-phosphate ketol-isomerase | alpha-D-Glucose 6-phosphate <===> beta-D-Glucose 6-phosphate |
| --- | --- | --- | --- | --- |
| ec:5.3.1.9 | Helo_1718 Helo_4245 | R02740 | alpha-D-Glucose 6-phosphate ketol-isomerase | alpha-D-Glucose 6-phosphate <===> beta-D-Fructose 6-phosphate |
| ec:4.1.1.49 | Helo_1685 | R00341 | phosphoenolpyruvate-forming) | ATP + Oxaloacetate ===> PEP + CO2 + ADP |
| ec:5.4.2.1 | Helo_1030 Helo_1820 Helo_3004 | R01518 | 2-Phospho-D-glycerate 2,3-phosphomutase | 2-Phospho-D-glycerate <===> 3-Phosphoglycerate |
| ec:5.3.1.1 | Helo_4141 | R01015 | D-glyceraldehyde-3-phosphate aldose-ketose-isomerase | Glyceraldehyde 3-phosphate <===> Glycerone phosphate |
| ec:1.2.4.1 | Helo_3572 | R00209 | pyruvate dehydrogenase complex | Pyruvate + CoA + NAD <===> CO2 + Acetyl-CoA + NADH + H+ |
| ec:4.2.1.11 | Helo_3749 | R00658 | 2-phospho-D-glycerate hydro-lyase (phosphoenolpyruvate-forming) | 2-Phospho-D-glycerate <===> PEP + H2O |
| ec:1.2.1.3 | Helo_2817 | R00710 | Acetaldehyde:NAD+ oxidoreductase | Acetaldehyde + H2O + NAD <===> NADH + H+ + Acetate |
| ec:2.7.1.69 | Helo_3696 Helo_1921 Helo_3099 | R02738 | Protein-N(pai)-phosphohistidine:sugar N(pai)-phosphotransferase | Protein N-pros-phosphohistidine + Glucose <===> Protein histidine + alpha-D-Glucose 6-phosphate |
| ec:2.7.1.40 | Helo_4243 Helo_1605 | R00200 | ATP:pyruvate 2-O-phosphotransferase | Pyruvate + ATP <=== PEP + ADP |
| ec:2.3.1.12 | Helo_3571 Helo_2372 | R00209 | pyruvate dehydrogenase complex | Pyruvate + CoA + NAD <===> CO2 + Acetyl-CoA + NADH + H+ |
| ec:4.1.2.13 | Helo_1183 Helo_1180 | R01070 | beta-D-fructose-1,6-bisphosphate D-glyceraldehyde-3-phosphate-lyase (glycerone-phosphate-forming) | beta-D-Fructose 1,6-bisphosphate <===> Glyceraldehyde 3-phosphate + Glycerone phosphate |
| ec:1.8.1.4 | Helo_3110 | R08549 | 2-Oxoglutarate dehydrogenase complex | CoA + NAD + 2-Oxoglutarate <===> CO2 + NADH + H+ + Succinyl-CoA |
| ec:1.8.1.4 | Helo_3110 | R00209 | pyruvate dehydrogenase complex | Pyruvate + CoA + NAD <===> CO2 + Acetyl-CoA + NADH + H+ |
| ec:6.2.1.1 | Helo_2142 Helo_3563 | R00316 | ATP:acetate adenylyltransferase | ATP + Acetate <===> PPi + Acetyl adenylate |
| ec:6.2.1.1 | Helo_2142 Helo_3563 | R00236 | acetyl adenylate:CoA acetyltransferase | CoA + Acetyl adenylate <===> Acetyl-CoA + AMP |
| ec:1.2.1.12 | Helo_4242 Helo_1182 Helo_2131 Helo_2214 | R01061 | D-glyceraldehyde-3-phosphate:NAD+ oxidoreductase (phosphorylating) | NAD + Glyceraldehyde 3-phosphate + Phosphate <===> NADH + 1,3-Bisphospho-D-glycerate + H+ |
| ec:2.7.2.3 | Helo_1181 | R01512 | ATP:3-phospho-D-glycerate 1-phosphotransferase | ATP + 3-Phosphoglycerate <===> 1,3-Bisphospho-D-glycerate + ADP |
| ec:1.1.1.27 | Helo_1046 | R00703 | (S)-Lactate:NAD+ oxidoreductase | NAD + L-Lactate <===> Pyruvate + NADH + H+ |
| ec:2.7.1.11 | Helo_2186 | R04779 | ATP:D-fructose-6-phosphate 1-phosphotransferase | beta-D-Fructose 6-phosphate + ATP ===> beta-D-Fructose 1,6-bisphosphate + ADP |

Other reactions

| ec:2.7.3.9 | | Helo_1696 Helo_3698 Helo_3100 | R02628 | Phosphoenolpyruvate:protein-L-histidine N-pros-phosphotransferase | PEP + Protein histidine ===> Pyruvate + Protein N-pros-phosphohistidine |
| --- | --- | --- | --- | --- | --- |
| ec:2.6.1.1 | | Helo_2764 Helo_2013 Helo_4120 | R00355 | L-Aspartate:2-oxoglutarate aminotransferase | L-Aspartate + 2-Oxoglutarate <===> Glutamate + Oxaloacetate |
| ec:1.4.1.2 | | Helo_3049 | R00243 | L-Glutamate:NAD+ oxidoreductase (deaminating) | Glutamate + H2O + NAD <===> NH3 + NADH + H+ + 2-Oxoglutarate |
| ec:4.1.3.1 | | Helo_3070 | R00479 | isocitrate glyoxylate-lyase (succinate-forming) | Isocitrate <===> Glyoxylate + Succinate |
| ec:2.7.4.3 | | Helo_3570 | R00127 | ATP:AMP phosphotransferase | AMP + ATP <===> 2.0 ADP |
|  | Helo_3664 | |  | DoeB | H2O + N-alpha-Acetyldiaminobutyrate ===> L-2,4-Diaminobutyrate + Acetate |
|  | Helo_3665 | |  | DoeA | Ectoine + H2O <===> N-alpha-Acetyldiaminobutyrate |
|  |  | |  | ATP load | H2O + ATP ===> Phosphate + ADP |
|  |  | |  | NADH NADPH conversion | NAD + NADPH <===> NADH + NADP |

**Oxidative Phosphorylation**

|  |  |  | NADH oxidation | NADH + O2 + H+ + 1.7 Phosphate + 1.7 ADP ===> 1.7 H2O + NAD + 1.7 ATP |
| --- | --- | --- | --- | --- |
|  |  |  | FADH oxidation | O2 + AH2 + H+ + 1.1 Phosphate + 1.1 ADP ===> 1.1 H2O + 1.1 ATP + A |

**Table S5.** Bacterial strains and plasmids used in this study.

| Strain or plasmid | Relevant genotype and/or description*^a^* | Source or reference |
| --- | --- | --- |
| *H. elongata* |  |  |
| DSM 2581^T^ | type strain | DSMZ*^b^* |
| KB1 | Δ*ectA* | (Grammann et al., 2002) |
| KB41 | Δ*doeA* | This study |
| KB42 | Δ*doeB* | This study |
| KB47 | Δ*doeC* | This study |
| KB48 | Δ*doeD* | This study |
| KB49 | Δ*eutB* | This study |
| KB50 | Δ*eutC* | This study |
| SB1 | Δ*ectB* | This study |
| SB1.1 | Δ*ectB,* Δ*doeD* | This study |
| KB2.11 | Δ*teaABC* | This study |
| KB2.13 | Δ*teaABC*, Δ*doeA* | This study |
| *E. coli* |  |  |
| BL21 | F^-^ *ompT hsdS*_B_ (r_B_^-^m_B_^-^) *gal dcm rne131* (DE3) | Invitrogen |
| DH5α | F^-^ φ*80dlacZDM15* Δ(*lacZYA-argF*) *U169 recA1 hsdR17* (*rK^-^ mK^+^*) *supE44* Δ^-^  *thi-1 gyrA relA1* | (Hanahan, 1983) |
| S17-1 | *thi pro hsdR^-^ hsdM*^+^ *recA*; Tp^r^, Sm^r^ | (Simon et al., 1983) |
| Plasmids |  |  |
| pK18*mobsacB* | Km^r^ , *mob,* *sacB* | (Schäfer et al., 1994) |
| pJB3Cm6 | Cm^r^ | (Blatny et al., 1997) |
| pKSB7 | pJB3Cm6::*doeA;* Cm^r^ | This study |
| pJSB3 | pJB3Cm6::*doeB;* Cm^r^ | This study |
| pET101 | Ap^r^ | Invitrogen |
| pKSB11 | pET101::*doeA*, Ap^r^ | This study |

*^a^* Abbreviations of antibiotics: Cm, chloramphenicol; Km, kanamycin; Ap, ampicillin; Sm, streptomycin; Tp, trimethoprim

*^b^* DSMZ: Deutsche Sammlung von Mikroorganismen und Zellkulturen, Braunschweig, Germany

**Table S6.** Locus tag of genes similar to *ect* genes, *ask*, and *doe* genes depicted in Figure 2, Figure 4, and Figure 7, and accession data of genome sequences of corresponding organisms.

| **Organism and Accession Number Genome Sequence** | **Gene Name** | Locus Tag |
| --- | --- | --- |
| *Acidiphilium cryptum* JF-5  CP000697 | *ectABCD, ask* | Acry_3008, 3009, 3010, 3011, 3012 |
| *Agrobacterium tumefaciens* C58  AE007870 | *doeCD*  *eutBC*  *doeAB* | Atu4762, 4761  Atu4759, 4758  Atu4757, 4756 |
| *Alkalilimnicola ehrlichii* MLHE-1  CP000453 | *ectC*  *ectAB*  *ectD* | Mlg_1190  Mlg_1192, 1191  Mlg_0392 |
| *Alkanivorax borkumensis* SK2  AM286690 | *ectABC*  *ectD* | ABO_2150, 2151, 2152  ABO_0023 |
| *Bacillus clausii* KSM-K16  AP006627 | *ectABC*  *ectD* | ABC0334, 0335, 0336  ABC3489 |
| *Bacillus halodurans* C-125  BA000004 | *ectABC* | BH0920, 0919, 0918 |
| *Blastospirellula marina* DSM 3645  AANZ00000000 | *ectABCD* | DSM3645_02398, 02393, 02388, 02383 |
| *Bordetella bronchiseptica* RB50  BX470250 | *ectABCD* | BB3220, 3219, 3218, 3217 |
| *Bordetella parapertussis* | *ectABCD* | BPP1888, 1889, 1890, 1891 |
| *Bordetella petrii* DSM 1280  AM902716 | *ectABCD* | Bpet1981, 1982, 1983, 1984 |
| *Burkholderia cenocepacia* AU1054  CP000380 | *doeABXCD* | Bcen_5803, 5802, 5801, 5800, 5799 |
| *Burkholderia cenocepacia* HI2424  CP000460 | *doeABXCD* | Bcen*2424*_6167, 6166, 6165, 6164, 6163 |
| *Burkholderia phymatum* STM815  CP001044 | *doeABXCD* | Bphy_3862, 3861, 3860, 3859, 3858 |
| *Burkholderia vietnamiensis* G4  CP000616 | *doeABXCD* | Bcep*1808*_5462, 5461, 5460, 5459, 5458 |
| *Burkholderia xenovorans* LB400  CP000272 | *doeA*  *doeBXCD* | Bxe_C0063  Bxe_C0058, C0059, C0060, C0061 |
| *Chromohalobacter salexigens* DSM 3043^T^  CP000285 | *ectABC,*  *ectD*  *ectE*  *doeABX*  *doeCD* | Csal_1875, 1876, 1877  Csal_0542  Csal_3003  Csal_2732, 2731, 2730  Csal_2724, 2723 |
| *Geobacillus thermodenitrificans* NG80-2  CP000557 | *doeA* | GTNG_2272 |
| *Hahella chejuensis* KCTC 2396  CP000155 | *ectABCD*  *doeA* | HCH_01510, 01509, 01508, 01507  HCH_06289 |
| *Haloferax volcanii* DS2  CP001953 | *doeA* | HVO_B0267 |
| *Halomonas elongata* DSM 2581^T^  FN869568 | *ectABC*  *ectD*  *doeABXCD* | Helo_2588, 2589, 2590  Helo_4008  Helo_3665, 3664, 3663, 3662, 3661 |
| *Halorhodospira halophila* SL1  CP000544 | *ectABC* | Hhal_1732, 1733, 1734 |
| *Halorubrum lacusprofundi* ATCC 49239  CP001365 | *doeA* | Hlac_1051 |
| *Herminiimonas arsenicoxydans* ULPAs1  CU207211 | *ectABCD* | HEAR3380, 3379, 3378, 3377 |
| *Hyphomonas neptunium* ATCC 15444  CP000158 | *ectABCD, ask* | HNE_1639, 1640, 1641, 1642, 1643 |
| *Jannaschia* sp.CCS1  CP000264 | *doeABD* | Jann_0849, 0850, 0851 |
| *Janthinobacterium* sp. Marseille  CP000269 | *ectABCD* | mma_3601, 3600, 36599, 36598 |
| *Marinobacter hydrocarbonoclasticus* DSM 11845  CP000514 | *ectAB, ask,*  *ectC, ectC, ectC,*  *ectD, ectD* | Maqu_0147, 0148, 0149  Maqu_0079, 0616, 0444  Maqu_1849, 3892 |
| *Marinomonas* sp. MWYL1  CP000749 | *ectABCD, ask* | Mmwyl1_1158, 1159, 1160, 1161, 1162 |
| *Mesorhizobium loti* MAFF303099  BA000012 | *doeDCX,*  *eutBC*  *doeAB* | mll7127, mll7178, mll7129  mlr7138, mlr7139  mlr7141. mlr7142 |
| *Methylophaga thiooxidans* MDS010  ABXT00000000 | *ectABC, ask* | MDMS009_1763, 2070, 2081, 1862 |
| *Mycobacterium gilvum* PYR-GCK  CP000656 | *ectABCD* | Mflv_4832, 4833, 4834, 4835 |
| *Mycobacterium* sp. JLS  CP000580 | *ectABCD* | Mjls_4417, 4418, 4419, 4420 |
| *Mycobacterium* sp. MCS  CP000384 | *ectABCD* | Mmcs_4190, 4191, 4192, 4193 |
| *Mycobacterium vanbaalenii* PYR-1  CP000511 | *ectABCD* | Mvan_5274, 5273, 5272, 5271 |
| *Natrialba magadii* ATCC 43099  CP001933 | *doeA* | Nmag_3760 |
| *Nitrosococcus oceani* ATCC 19707  CP000127 | *ectABD*  *ectC, ask* | Noc_1562, 1561, 1560  Noc_1028, Noc_1029 |
| *Nitrosopumilus maritimus* SCM1  CP000866 | *ectABCD* | Nmar_1346, 1345, 1344, 1343 |
| *Nocardia farcinica* IFM10152  AP006618 | *ectABCD* | nfa27160, 27170, 27180, 27190 |
| *Oceanobacillus iheyensis* HTE831  BA000028 | *ectBC*  *doeA* | OB0518, 0519  OB2950 |
| *Ochrobactum anthropi* ATCC 49188  CP000759 | *doeXCD*  *eutBC*  *doeAB* | Oant_3470, 3471, 3472  Oant_3469, 3468  Oant_3467, 3466 |
| *Phenylobacterium zucineum HLK1*  CP000747 | *ectABCD, ask* | PHZ_c1335, c1336, c1337, c1338, c1339 |
| *Pseudomonas aeruginosa* PA7  CP000744 | *doeABXCD* | PSPA7_4378, 4379, 4380 4381, 4382 |
| *Pseudomonas stutzeri* A1501  CP000304 | *ectABCD, ask* | PST_0181, 0180, 0179. 0178, 0177 |
| *Rhizobium etli* CFN42  CP000138 | *doeXCD*  *eutBC*  *doeAB* | RHE_PF00200, PF00201, PF202  RHE_PF00182, 00181  RHE_PF00180, PF00179 |
| *Rhizobium leguminosarum* bv. *viciae* 3841  AM236086 | *doeDCX*  *eutBC*  *doeAB* | pRL120045, 120044, 120043  pRL120052, 120053  pRL120054, 120055 |
| *Roseobacter denitrificans* OCh 114  CP000362 | *doeAB* | RD1_3474, 3475 |
| *Ruegeria* *pomeroyi* DSS-3  CP000031 | *doeABXCD* | SPO1140, 1139, 1138, 1137, 1136 |
| *Ruegeria* sp. TM1040  CP000377 | *ectABC*  *ask*  *doeA*  *doeBXCD* | TM*1040*_0550, 0551, 0552, 0553, TM*1040*_0554  TM*1040*_1921  TM*1040*_2694, 2693, 2692, 2691 |
| *Saccharophagus degradans* 2-40  CP000282 | *ectABC, ask*  *ectD* | SDE_1189, 1190, 1191  Sde_1259 |
| *Sinorhizobium meliloti* 1021  AL591985 | *doeXCD*  *eutBC*  *doeAB* | SM_b20425, b20424, b20423  SM_b20432, b20433  SM_b20434, b20435 |
| *Sphingopyxis alaskensis* RB 2256  Sala_2949 | *ectABCD, ask* | Sala_2949, 2950, 2951, 2952, 2953 |
| *Streptomyces avermitilis* MA-4680  BA000030 | *ectABCD* | SAV_6398, 6397, 6396, 6395 |
| *Streptomyces coelicolor* A3  AL645882 | *ectABCD* | SCO1864, 1865, 1866, 1867 |
| *Thermobifida fusca* YX  CP000088 | *ectABC* | TFU_0300, 0301, 0302 |
| *Thiomicrospira crunogena* XCL-2  CP000109 | *ectABC, ask*  *doeA* | Tcr_0518, 0519, 0520, 0521  Tcr_0221 |
| *Verminephrobacter eiseniae* EF01-2  CP000542 | *doeABXCD* | Veis_2149, 2148, 2147, 2146, 2145 |
| *Vibrio cholerae* O395  CP000626 | *ectABC, ask* | VC*O395*_0409, 0410, 0411, 0412 |
| *Vibrio fischeri* ATCC 700601  CP000021 | *ectABC, ask* | VF_A1122, A1123, A1124, A1125 |
| *Vibrio harveyi* ATCC BAA-1116  CP000789 | *ectABC, ask* | VIBHAR_02454, 02453, 02453, 02451 |
| *Vibrio parahaemolyticus* RIMD 2210633  BA000031 | *ectABC, ask* | VP1722, 1721, 1720, 1719 |
| *Vibrio* *splendidus* LGP32  FM954973 | *ectABC, ask* | VS_II0066, II0067, II0068, II0069 |
| *Wolinella succinogenes* DSM 1740  BX571656 | *ectABC* | WS0854, 0855, 0856 |

### Supporting Information: Orthologous group (COG) assignment

Orthologous group assignments for *C. salexigens* and other species were taken from eggNOG 2.0 (Muller et al., 2010), which extends existing COGs (Tatusov et al., 2003) (based on 66 completely sequenced genomes) to a set of 630 complete genomes. Unassigned proteins were then clustered into NOG (non-supervised orthologous groups).

COG assignments for *H. elongata* were made by a PERL script based on BlastP comparisons to the eggNOG 2.0 protein sequences. At maximum, the first 10 hits were analyzed. Hits were considered when they were better than “e-10” and when the blast score was at least 50% of the best blast score. The eggNOG database associates COGs with protein domains and this information was used in the *H. elongata* assignment. COGs were assigned when the alignment covered at least 70% of the domain associated with the COG.

### Supporting Information: Validation of the COG assignment procedure

The same COG has been assigned for 98.4% of the 2367 proteins with a bidirectional best blast between *H. elongata* and *C. salexigens*. This includes (a) 92.2%, where the same COG (set) was assigned, (b) 2.6% where neither the *H. elongata* nor the *C. salexigens* protein had an assigned COG, and (c) 3.6% of the proteins where a corresponding COG was assigned but where differences were seen due to additional COG assignments (3.3% in *H. elongata*, 0.3% in *C. salexigens*). Thus, discrepancies are minimal. In less than 1%, a COG was assigned to only one protein of a bidirectional best blast pair. There were only 16 proteins with assignment of discrepant COGs. Of these, 11 may be non-orthologous. They belong to a minor set of 65 bidirectional best blast hits where either sequence identity is below 30% or less than half of the protein was represented in the partner. In summary, the COG assignments of the PERL script are of sufficient reliability for subsequent analysis. The script may have a slight tendency for an overprediction.

### Supporting Information: Searching for high salt related COGs

In a search for COGs related to high salt environments, we analyzed COG members in three halophilic organisms (H: *Chromohalobacter salexigens*, *Bacillus halodurans*, *Salinibacter ruber*), 10 marine organisms (M) and 14 standard organisms (S) like *E .coli* and *Bacillus subtilis*. The complete list of organisms can be found in Table S2. An attempt was made to select the standard organisms from similar taxonomic branches as used for halophilic and marine organisms.

COGs, which are occupied in *H. elongata* were checked for occupancy in each of these species. COGs were classified as “standard” when they were occupied in at least 60% of the standard organisms (i.e., “percent occupancy” in standard organisms at least 60%). To identify “halophilic” COGs, the percent occupancy in halophilic/marine organisms was compared to the percent occupancy in standard organisms. COGs with a two-fold higher occupancy value in halophiles/marines were classified as halophilic when they occurred in a sufficient number of organisms: Either in 2 of the 3 halophilic organisms or in 5 of the 13 halophilic/marine organisms. By this approach, we classified 97 COGs as halophilic. Of these, 3 have at least five members, 12 have 2-4 members and 82 have a single member in *H. elongata*.

Three COGs contain the three subunits of the DctPQM-type of TRAP transporter, which is expanded in *H. elongata* but has only a single member in *C. salexigens*. This is exemplified by the *H. elongata* TRAP transporter Helo_2801 (COG4663, substrate-binding subunit), Helo_2802 (COG4665, small transmembrane subunit) and Helo_2803 (COG4664, large transmembrane subunit). Each of the three subunits from this transporter has a bidirectional best blast hit to the corresponding subunit of the *C. salexigens* TRAP transporter. However, sequence identity levels (30%, 38%, and 44%) are well below the average for protein pairs from these two species (69%). Thus, these two TRAP transporters probably do not represent orthologs and thus may have distinct substrates. *H. elongata* has five additional TRAP transporters of this subtype (Helo_1504, Helo_1503, Helo_1502; Helo_1776, Helo_1777, Helo_1778; Helo_3996, Helo_3997, Helo_3998; Helo_4268, Helo_4266, Helo_4267; Helo_1558, Helo_1558_A, Helo_1559). Several of the subunits are also or only assigned to the high-occupancy TRAP transporter COGs COG3030, COG1593, or COG1638.

Most of the halophilic COGs are occupied in both, *H. elongata* and *C. salexigens*. There are only a few exceptions. (i) Besides *H. elongata*, COG2176 (DNA polymerase III, alpha subunit (gram-positive type) occurs in *B. halodurans* as well as *S. ruber*, in four marine organisms including *Oceanobacillus* *iheyensis* but in only two standard organisms (*B. subtilis*, *B. cereus*). This enzyme is most prominent in Firmicutes. (ii) COG2374 (Predicted extracellular nuclease) occurs in *S. ruber* and 5 marine organisms but only in standard organisms *B. cereus* and *Nostoc*. This COG is occupied in only few species of various taxonomic branches. (iii) COG3720 (Putative heme degradation protein) occurs in *S. ruber* and 5 marine organisms but also in 3 standard species. It is found preferentially in proteobacteria. (iv) COG3245 (Cytochrome c5) occurs in six marine organisms but only 3 standard organisms. It is preferentially found in proteobacteria. (v) A similar pattern is also seen for COG3508 (Homogentisate 1,2-dioxygenase).

In addition, there are several hypothetical proteins from various COGs/NOGs, which occur only in *H. elongata* and not in *C. salexigens*. (i) NOG12323 occurs in *B. halodurans* and 4 marine organisms but only in the standard organism *B. subtilis*. (ii) COG3487, COG3488, COG3489, and COG3490 occur in the same set of 6 marine organisms but only 2-3 standard organisms. Among the halophilic COG members, which are common to *H. elongata* and *C. salexigens* are ectoine synthase (NOG8290) and ectoine hydroxylase (NOG81762). The latter has two paralogs in *C. salexigens* (*ectD* and *ectE*) but only one member in *H. elongata* (*ectD*). Other enzymes related to ectoine biosynthesis belong to functionally heterogenous COGs, e.g. EctA in COG0454, a COG, which contains various acetyltransferases.

### Supporting Information: Comparison of protein pI values between *H. elongata* and other organisms

We analyzed if the *H. elongata* proteome has a shift in its pI pattern as an adaptation to the high-salt environment. We used the set of organisms selected for identification of halophile-specific COGs for the analysis of the corresponding protein pair. (In few cases, proteins from query organisms were classified in eggNOG but their sequences were not available in the eggNOG sequences file and these were excluded from the analysis). For each protein pair, the pI difference was computed. These data were averaged for each of the compared organisms (Table S3). The minimum number of protein pairs was 517 for *Prochlorococcus* *marinus* (with the exception of *Aquifex* *aeolicus*, which had 508 pairs). The maximum was 952 for *Marinobacter* *aquaeolei* (with the exception of *Chromohalobacter* *salexigens* which had 1114 pairs).

The following conclusions can be drawn: (i) pI shifts are frequently only small. For 13 of the 27 organisms, the pI shift is less then 0.3 pI units. (ii) pI shifts tend to be in the alkaline direction, indicating that the *H. elongata* proteome has evolved slightly into the acidic direction. Only two organisms have a more acidic proteome, two have no shift at all and 23 organisms have a (slightly) more alkaline proteome. (iii) Larger pI shifts seem indicative of specific adaptations in the query organism rather than in *H. elongata*. *S. ruber*, which is one of the few bacteria using the salt-in strategy to cope with osmotic stress, has a proteome, which is more acidic by 0.7 pI units. Five organisms have a larger shift towards alkaline pI between 0.6 and 0.9 pI units. *A. aeolicus* stands out with a shift by 1.3 units.

### Supporting Information: Reconstruction and modeling of the metabolic network

***Flux balance analysis***: A Flux Balance Analysis (FBA) (Varma and Palsson, 1993b, a) approach was selected to create a mathematical model of ectoine metabolism. In brief, such a model represents a metabolic network, consisting of biochemical reactions and their stoichiometries and reversibilities. The enzymes identified in the annotated genome of *H. elongata* were connected to one or more reactions using the KEGG database (Kanehisa and Goto, 2000) and their reversibilities were determined according to standard biochemical knowledge and the BRENDA database (Schomburg et al., 2002). The main advantage of such a formalized mathematical representation is the possibility to apply rigorous tests to validate the consistency of the network and identify biochemically relevant steady states.

A context can be defined for the metabolic network by defining the inputs, outputs and reversible exchanges through the system’s boundary. FBA can then compute the steady state flux through every reaction that can achieve a certain biological goal (e.g. maximal ATP production). Such a result is called *flux distribution*. FBA analysis can only be successful if a consistent and formally correct metabolic network can be extracted from the genome annotation. Thus, this mathematical technique may uncover genome annotation problems and enforce error corrections. This exemplifies how mathematical modeling provides a major improvement over classical paper and pencil stochiometric calculations. It is important to clarify that FBA is not used here to establish what is optimized by the cell; it is expected that different functions will be optimized in different conditions. The aim of the analysis is to explore how these biological goals can be reached within a certain metabolic network structure, and to what extent they may compete or conflict with one another.

A minimal model for conversion of glucose to ectoine is presented. As this model *excludes* growth (biomass production), only metabolism of the aspartate family of amino acids has been included. It should be emphasized that (i) the model is based on a detailed manual curation of an initial automatic genome annotation and, (ii) the model has been developed from a richer model by reduction of all reactions that have no impact on the central biological topic being addressed.

***Annotation of enzymes***: A well-annotated genome is a prerequisite for a credible metabolic model. Automated function prediction was performed using the Metanor tool of the GenDB genome annotation system (Meyer et al., 2003). Automated annotation is, unfortunately, subject to a substantial error rate. The PRIAM program (Claudel-Renard et al., 2003) provides a second, complementary, enzyme assignment strategy. The consistency between automatically assigned EC numbers and PRIAM-based EC numbers has been checked in a manual curation effort. During this process, also experimental data from the literature and knowledge on the physiology of the organism were taken into account. Reactions of central and amino acid metabolism, especially those relevant for ectoine metabolism, have been extensively manually curated.

Several enzyme assignments were critically evaluated in later stages of the project, e.g. when model-based metabolic network validation indicated a potential genome annotation error. Also, unexpected modeling results were critically evaluated in order to validate that the conclusions are biochemically sound. For details of such evaluations see below.

A total of 1265 proteins from *H elongata* received complete or incomplete EC numbers.

***Network reconstruction***: We describe the process where a collection of input data (here, EC numbers) is converted to a mathematically sound model representing a biological process. In the network reconstruction described, enzyme assignments are converted into a collection of reactions with fully specified stoichiometries.

First, EC numbers have to be converted to reactions. There are several EC numbers, which refer to more than one reaction (e.g. enzymes which are promiscuous with respect to substrate or coenzyme). On the other hand, several reactions may be associated with more than one EC number (e.g. in case where a coenzyme-specific enzyme and a promiscuous enzyme have been assigned distinct EC numbers). Thus, the conversion between EC numbers and reactions is not straightforward but is largely facilitated due to the resources available from the KEGG database (Kanehisa and Goto, 2000). Reactions, which are not represented in the KEGG database, like the newly detected ectoine degradation pathway, were added manually. All reactions considered in the current model are listed in Table S4.

All reactions are integrated into a large stoichiometric matrix, which is the key element of Flux Balance Analysis (and other techniques of “constraint-based modeling”). In this matrix, each compound (substrate/product) represented anywhere in the metabolic network has its row and each (enzymatic) reaction has its column. The stoichiometric coefficients of each reaction are added to the matrix at the corresponding position. As an example, the reaction substrate receives -1 and the product +1 if a single substrate molecule is converted to a single product molecule. Overall, this creates a mathematical representation of metabolism. This representation allows applying standard mathematical tools, which in turn may identify potential genome annotation errors (e.g. missing enzyme assignments). In other words, using a mathematical formalism adds another layer of checking of the genome annotation.

Considered reactions are based on enzyme assignments during genome annotation and its manual curation. In some cases, formal checking will identify pathway gaps where a pathway is incomplete due to the missing annotation of one or a few enzymes. It may happen that the pathway is known to be functional in the organism (e.g. because it can grow without the necessity to supply the corresponding compound in the growth medium) but attempts to identify the enzyme gene based on current knowledge are not successful. In such cases, the reaction may be set to exist despite the inability to identify a corresponding gene. In the restricted model reported in this manuscript, such pathway gaps have not been found. However, we added two general reactions without gene assignment. One reaction is a general ATP hydrolysis reaction, which we use to represent the *ATP load* of a cell. The other is a transhydrogenase reaction (NADH+NADP^+^ => NAD^+^+NADPH). Both reactions are discussed in more detail below.

After selection of reactions, reversibilities have to be assigned. Identifying the reactions that cannot be reversed is instrumental for the success of the analysis since it excludes biochemically unfeasible solutions. Reversibilities were determined according to standard biochemical knowledge and the BRENDA database (Schomburg et al., 2002). An example of the relevance of such a reversibility assignment, which has an impact on the modeling results, is discussed below in more detail.

The stoichiometry of each metabolic reaction of the network needs to be represented in the stoichiometric matrix. While this information is commonly available from the biochemical reaction itself, there are a few exceptions. One such exception is the re-oxidation of reduced coenzymes via the respiratory chain, and the subsequent generation of ATP from ADP and P_i_. This oxidative phosphorylation process is represented in the mathematical model by the P/O ratio, i.e. the number of ATP equivalents generated per oxygen molecule. The stoichiometry of oxidative phosphorylation depends on various details of ATP synthase and the respiratory chain complexes, which cannot be obtained from genome sequence analysis. The setting of the P/O ratio is detailed in a short paragraph below.

***Network context and operation scenarios***: A context can be defined for the metabolic network by defining the exchanges through the systems boundary that are possible in a certain situation. Only the inputs (e.g. glucose) or outputs (e.g. ectoine) can be depleted or accumulated. Flux Balance Analysis is performed under the assumption that all other metabolites are balanced in the steady state process. In terms of Flux Balance Analysis, the “state of the network” is defined by the values of the rates of all its reactions.

For the outputs, theoretical yields can be calculated (such as maximal ectoine production or maximal ATP generation). Such a calculation can be stated as solving an optimization problem: find the rates of all the reactions that are compatible with the steady state condition and that achieve a certain biological goal (e.g. maximize ectoine production). The result is called *flux distribution*. The reported values are yields, because the problem is normalized to one of the inputs (which in this manuscript always is set to 100 molecules of glucose).

We want to stress again that Flux Balance Analysis can only be successful if a consistent and formally correct metabolic network can be extracted from the genome annotation. Thus, this mathematical technique may uncover genome annotation problems and enforce error corrections. In order to ensure consistency between genome annotation and modeling, relevant pathways and reactions were automatically extracted from the HaloLex system ([www.halolex.mpg.de](http://www.halolex.mpg.de/)) (Pfeiffer et al., 2008a) and filtered by a data import pipeline. This strategy allowed to always regenerating the model from the genome annotation data at any time.

We applied Flux Balance Analysis for several scenarios, each requesting to optimize for a biological goal (like maximal ATP generation or maximal ectoine production). It is important to clarify that Flux Balance Analysis is not used to establish what is optimized by the cell; it is expected that different functions will be optimized in different conditions. The aim of the analysis is to explore how these biological goals can be reached, based on a certain metabolic network. Additional constraints were imposed, e.g. maximization of ectoine output for different ATP loads.

***The minimal model was generated by supervised reduction of a rich model***: As a first step, we wanted to provide a minimal model that allows to analyze conversion of glucose to ectoine. In order to avoid misunderstandings, we have to stress that this minimal model was build from a larger model by a reduction process. A series of KEGG maps were selected which represent central metabolism and biosynthesis of several amino acids. For all EC numbers on one of these KEGG maps, which are assigned in the annotated *H. elongata* genome, the corresponding reactions were added to the initial model. Mathematical tests were applied to discover reactions that are disconnected from the rest of the network. Unconnected reactions, which occur at the outer rim of the corresponding KEGG map and which are unrelated to ectoine biosynthesis or not present in the studied organism were removed from the model (e.g. reactions involving D-serine). Once a consistent model has been obtained, simulations were performed. Reactions, which proved unaffected under all scenarios explored, were then removed. The final model is the result of keeping only the core metabolism that is needed to explain ectoine metabolism.

Being confronted with a minimal model as the one presented, the reader may ask how realistic or useful such a model is. We are confident that the described procedure ensures generation of a realistic and useful model. To summarize, the minimal model has been derived from a richer model, including all metabolic pathways a biochemist would consider potentially relevant, by a supervised reduction process.

### Supporting Information: Details for various model-related aspects

***The P/O ratio***: The P/O ratio, the number of ATP molecules obtained by oxidative phosphorylation per molecule of oxygen consumed, is one of the parameters, which is needed in Flux Balance Analysis but cannot be easily obtained from genome annotation. Several different factors affect the P/O ratio like the structure of the electron transport chain, the stoichiometry of the ATPase (commonly defined by the subunit stoichiometry of the c-ring) or the proton leak (normally small).

There are no direct measurements of the P/O ratio for *H. elongata*, which therefore had to be approximated. This can be done by comparison to organisms for which experimental data or published approximations are available. The P/O ratio has been measured *in vivo* for *E. coli* under glucose limitation and excess and found to be 1.4 *±* 0.3 and 1.4 *±* 0.1 respectively (Noguchi et al., 2004). Modeling studies have estimated a P/O ratio of 1.3 for *Bacillus subtilis* (Sauer and Bailey, 1999).

The parameter estimations are further complicated by the fact that there are different P/O ratios for different substrates (NADH, FADH, succinate) and the overall ratio that can be measured is the average of all of them, weighted by their relative fluxes.

The electron transport chain in *H. elongata* is standard according to genome annotation. Some indirect evidence points towards a P/O ratio of 1.6 (Dötsch et al., 2008) or 2 (Maskow and Babel, 2001). Here, we have assumed an overall P/O ratio of 1.6, with values of 1.7 for NADPH and 1.1 for FAD and other reducing equivalents (collected in A:AH2). With this ratio, we generate 23 ATP per glucose molecule under aerobic conditions. The theoretically maximal P/O ratio, which is 3 for NADH and 2 for FADH, results in 38 ATP per glucose, a value used in the Oren review (Oren, 1999).

In order to evaluate the influence of the P/O ratio on the modeling results, all simulations were repeated with different P/O ratios ranging from the theoretical maxima of 3 and 2 to a minimum of 1 and 0.5. There were no qualitative changes in the results. The P/O ratio has only quantitative effects, influencing the fraction of glucose that is redirected to the TCA cycle at high ATP loads.

***The ATP load***: A general ATP hydrolysis step was added to account for the usage of ATP in processes that were not modeled (e.g. maintenance energy). This step is referred to as ATP load.

Besides maintenance energy, other processes contribute to the ATP load. An example are transport processes, e.g. those that may contribute to long-term regulation of ectoine biosynthesis. Such processes may be incorporated into more advanced models. Currently, the effect of these processes is restricted to their contribution to the ATP load.

***Alternative solutions***: The presence of equivalent alternatives (degenerate solutions) is frequent in Flux Balance Analysis and the number of such solutions grows with the size of the network. These solutions may reflect redundancy of the network that provides robustness to its metabolism by providing alternative pathways for the same conversion.

In other cases, alternative solutions may not be equivalent but may all be permissible due to reduced constraints. In this case, some of the alternative solutions may disappear when more stringent constraints are applied. As an example, the conversion of glucose to ectoine results in net generation of one ATP equivalent according to our model. When simulations are computed with the corresponding ATP load set to 1 ATP per glucose, only the most energy-efficient conversions are permissible. In simulations without ATP load, additional, less energy-efficient solutions become permissible.

An example is the PEP to oxaloacetate conversion, which is described in more detail below.

***Conversion of PEP to oxaloacetate***: Two alternative enzymes catalyze the direct interconversion of PEP and oxaloacetate. PEP carboxylase (EC 4.1.1.31, ATP neutral) and PEP carboxykinase (EC 4.1.1.49, one ATP required from oxaloacetate to PEP), both identified in *H. elongata* upon genome annotation. The question was if the latter reaction is reversible and thus would allow generation of one ATP by converting PEP to oxaloacetate.

It could be argued that the ATP in the PEP carboxykinase reaction is required to drive the reaction into the direction of PEP, in which case an ATP-conserving reaction from PEP to oxaloacetate, although theoretically possible, will not happen to an extent that is significant for the overall conversion of glucose to ectoine.

The priming of the TCA cycle through PEP carboxykinase occurs in mutants of *Bacillus subtilis* lacking pyruvate kinase (Zamboni et al., 2004) enabling them to grow. From this result, it can be concluded that PEP carboxykinase is reversible. However, the corresponding *B. subtilis* mutant grew only slowly, indicating that the reaction is kinetically unfavorable for thermodynamic reasons. We decided to treat PEP carboxykinase as irreversible in order to avoid a biochemically unfeasible solution.

To our surprise, modeling with an irreversible PEP carboxykinase still allowed a 100% ectoine yield at an ATP load of 1 ATP per glucose. When analyzing the network structure, we found that an alternative solution exists which converts PEP to oxaloacetate with the simultaneous generation of one ATP. PEP is converted to pyruvate through pyruvate kinase (EC 2.7.1.40), generating one ATP. Pyruvate is then converted to oxaloacetate in an ATP-neutral two-step reaction via malic enzyme (EC 1.1.1.38) and malate dehydrogenase (EC 1.1.1.37). This pathway is a stoichiometrically equivalent alternative solution to the PEP carboxykinase reaction.

As we did not find any argument that would disallow this pathway, we went ahead and computed if the conversion of glucose to ectoine with simultaneous generation of one ATP is thermodynamically feasible.

***Thermodynamics of the glucose to ectoine conversion***: The conversion of glucose to ectoine results in net generation of one ATP equivalent according to our model. Although stoichiometrically possible, it was important to calculate whether the production of one ATP per glucose is thermodynamically compatible with a full conversion to ectoine. The overall equation for the reaction is: glucose(*_aq_*) + 2 NH_3(_*_aq_*) *=>* ectoine(*_aq_*) + 4 *H*_2_*O*(*_l_*).

The following standard formation enthalpies (ΔH*^o^_f_*) were considered in this calculation: glucose(*_aq_*) -1262.4, ectoine(*_aq_*) -332.7, NH_3(_*_aq_*) -81.1 H_2_O(*_l_*) -285.8. Standard formation enthalpies were taken from (von Stockar et al., 1993) except for ectoine, which was calculated with Hyperchem software.

With these data, the reaction enthalpy can be computed as:

Δ*H^o^_r_* = 1262*.*4 + 2 * (81*.*1) *−* 332*.*7 + 4 * (*−*285*.*8) = *−*51*.*3

Although a very rough calculation, this result is compatible with ATP formation. The reaction enthalpy of ATP hydrolysis has been reported as -22.21 kJ/mol by Podolsky and Morales (1956) and -23 kJ/mol by Kodama and Woledge (1979)

***Phosphofructokinase***: It is currently not clear whether the *H. elongata* phosphofructokinase is a normal ATP-dependent enzyme (EC 2.7.1.11) or if it is pyrophosphate-dependent (EC 2.7.1.90). We annotated the enzyme (Helo_2186) as ATP-dependent while the homolog from *C. salexigens* (Csal_1534) is annotated as pyrophosphate-dependent. The basis for the latter annotation is not clear.

We analyzed the impact of this annotation ambiguity for our conclusion that an ATP can be generated by conversion of glucose to ectoine. In general, pyrophopshate is “cheaper” than ATP. As an example, pyrophosphate is released upon DNA and RNA polymerization. Normally, the pyrophosphate is destroyed rapidly (in order to ensure that DNA and RNA polymerization is irreversible). If *H. elongata* could use the pyrophosphate to phosphorylate fructose-6-phosphate, it would spare one ATP equivalent.

There is only one pyrophosphate-generating reaction in our current model, namely the conversion of acetate to acetyl-CoA (acetyl-CoA synthetase, EC 6.2.1.1) with concomitant hydrolysis of ATP to AMP. This is the energy-requiring step for the proposed ectoine synthesis/degradation cycle. Assuming an ATP-driven phosphofructokinase, we compute the energetic cost of this cycle as 2 ATP per turn. With a pyrophosphate-dependent phosphofructokinase this cost would be reduced to 1 ATP per turn as the released pyrophosphate could be reused by phosphofructokinase.

Taken together, although some details of the *H. elongata* phosphofructokinase are yet unknown, a pyrophosphate-dependent enzyme would even further support our conclusion as it would result in an even more favorable energetic balance.

***Glucose degradation***: Besides the Embden-Meyerhof pathway, *H. elongata* also contains enzymes of the Entner-Doudoroff pathway. Including this pathway in the simulations did not affect the result since the maximal amount of ATP from glucose to ectoine conversion was unaffected.

It is possible that two biochemical pathways operate in parallel although they catalyze the same overall conversion but with a different energy yield. The Entner-Doudoroff pathway is rejected as a suboptimal alternative when maximal theoretical yields are computed.

***Glutamate dehydrogenase***: Our model-based calculations differ from those in the Oren review with respect to the nitrogen assimilation step. Oren’s calculations assume nitrogen assimilation via glutamine synthetase (EC 1.4.1.13) while we assume assimilation via glutamate dehydrogenase (EC 1.4.1.2), which is more energy efficient and spares the equivalent of two ATP. The latter is based on the annotation of a NAD-dependent glutamate dehydrogenase (Helo_3049).

The annotation as an NAD-dependent enzyme is consistent with that of many homologs. Nevertheless, it is well known that coenzyme assignments by simple sequence comparison are not well substantiated. As explained below, we consider NADH and NADPH convertible in our current model. Although this is a simplification, we are confident that this is a valid assumption for most prokaryotic cells.

***Interconversion of NAD and NADP***: The cofactor specificity of enzymes is often difficult to assign according to sequence analysis. Therefore, in this initial approach to the metabolic network of *H. elongata*, NADH and NADPH are treated as equivalent. The model contains a transhydrogenase for the interconversion of reducing equivalents in the form of NADPH and NADH, which is considered to be energy-independent. Such a procedure is often used in this kind of models and enables overall calculations using the reducing equivalents as a single pool (Stephanopoulos et al., 1998).

Although the handling of NADH and NADPH as equivalent is a simplification, this may be considered appropriate for a prokaryotic cell. Any reversible enzyme, which is promiscuous with respect to NAD(P) and any corresponding pair of isoenzymes, will allow to establish equilibrium between these two coenzymes.

***Interconversion of ectoine and Nγ-Ac-DABA***: In ectoine biosynthesis, Nγ-Ac-DABA is converted to ectoine by EctC. We report here that DoeA hydrolyzes ectoine, which results in the formation of Nα-Ac-DABA and Nγ-Ac-DABA at a ratio of 2:1. Although the Nγ-Ac-DABA/ectoine conversion proceeds in both directions due to the combination of EctC and DoeA, each of the enzymes may only catalyze the conversion into one direction. This must be attributed to kinetic reasons associated with the catalytic details of the enzymatic reactions, as the combined activity of EctC and DoeA indicate that the Nγ-Ac-DABA/ectoine conversion should be in principle reversible. No ectoine hydrolysis activity of EctC could be detected *in vitro* (Ono et al., 1999). It is, however, possible that the *in vitro* measurements do not completely reflect the *in vivo* situation. For DoeA, we have only proven the hydrolysis of ectoine and have not addressed reversibility.

It should be stressed that the details of the Nγ-Ac-DABA/ectoine conversion do not have any impact on the model, including the proposed ectoine synthesis/degradation cycle. The only known fate of Nγ-Ac-DABA resulting from ectoine hydrolysis by DoeA is its reconversion to ectoine catalyzed by EctC. This is neutral with respect to all model parameters. In contrast, the other product of ectoine hydrolysis by DoeA, Nα-Ac-DABA, is deacetylated by the isomer-specific enzyme DoeB. This drives the overall reaction towards ectoine degradation. Due to the specificity of DoeB, the degradation pathway must proceed via the Nα-Ac-DABA intermediate.

***Hydroxyectoine***: Ectoine can be converted to hydroxyectoine by reaction that has not been included into the model, because it only occurs under certain stress conditions such as high temperatures. Therefore, we decided to exclude hydroxyectoine from the model for reasons of simplicity.

### Supporting Information: Results of the modeling of the metabolic network

Once a mathematical model is available, optimization tools can be applied to search in a large set of alternative pathways for those, which achieve certain goals. These mathematically sound tools will ensure that an exhaustive analysis is performed and that the (set of) optimal solution(s) is returned. We applied such tools to search for maximal ectoine production from glucose and, among those, for the most energy-efficient pathway. We have modeled ATP-utilizing processes (maintenance energy) as *ATP load*. Therefore, we requested maximal ectoine production imposing increasing ATP loads.

The metabolic reconstruction (Figure 8) is consistent with possible flux distributions (see above) that are equivalent in ectoine output but differ in ATP production (Figure 9 B, I-II). Overall, glucose is converted to two molecules of PEP/pyruvate, one of which is converted to acetyl-CoA, the other to aspartate semialdehyde via oxaloacetate. Ectoine is then produced from one molecule each of aspartate semialdehyde and acetyl-CoA.

The flux distribution marked as (I) in Figure S5 B is a representative of solutions where the conversion from glucose to ectoine is ATP-neutral. In the solution that is displayed, PEP is converted to oxaloacetate through PEP carboxylase (EC 4.1.1.31). This particular case has been used for the previous calculations on the energetic costs of ectoine synthesis presented by Oren (1999), which differs only in the way nitrogen is assimilated (see main text).

However, the network arising from our annotated genome shows an alternative solution, in which a total conversion of glucose into ectoine is possible with the simultaneous generation of ATP. The additional ATP can be obtained by using a different pathway to convert PEP to oxaloacetate and this is illustrated in the flux distribution where PEP is converted to pyruvate generating one molecule of ATP (Figure S5 B (II)). Pyruvate is then converted to oxaloacetate through the action of the malic enzyme (EC 1.1.1.38) and malate dehydrogenase (EC 1.1.1.37). An estimation of the reaction enthalpy for the conversion of glucose to ectoine shows that this pathway is not only stoichiometrically but is also thermodynamically feasible (see above).

When the ATP load (maintenance energy) rises beyond the threshold of one ATP/glucose (Figure S5 B, III), a full conversion of glucose into ectoine is no longer possible and part of the flux must be redirected towards energy production. The most efficient way of achieving this with the proposed pathways is a superposition of the flux distribution that results in maximal ectoine production with a fully active TCA cycle, which is required for maximum ATP production. Such superposition is a weighted average in which the contribution of ectoine production and the TCA cycle to the metabolic fluxes are determined solely by the total ATP demand.

The novel degradation pathway of ectoine that we have introduced here may result in an apparent futile cycle, in which ectoine can be synthesized and degraded simultaneously resulting in a net conversion of acetyl-CoA into acetate. Re-conversion of acetate to acetyl-CoA costs the equivalent of two ATP. Therefore, if such a cycle were active, it would result in an increased apparent cost of two ATPs per turn for ectoine synthesis.

A simulation of such a scenario is depicted in Figure S5 B (IV). The optimal ectoine yield, and its corresponding flux distribution, is identical to what would result in an inactive cycle, but at higher ATP demand. The model shows that the ectoine cycle has no other metabolic effect besides using ATP.

**References**

Dötsch, A., Severin, J., Alt, W., Galinski, E.A., and Kreft, J.-U. (2008) A mathematical model for growth and osmoregulation in halophilic bacteria. *Microbiology* **154:** 2956–2969.

Hanahan, D. (1983) Studies on transformation of Escherichia coli with plasmids. *J Mol Biol* **166:** 557–580.

Kanehisa, M., and Goto, S. (2000) KEGG: kyoto encyclopedia of genes and genomes. *Nucleic Acids Res* **28:** 27–30.

Kodama, T., and Woledge, R.C. (1979) Enthalpy changes for intermediate steps of the ATP hydrolysis catalyzed by myosin subfragment-1. *J Biol Chem* **254:** 6382–6386.

Noguchi, Y., Nakai, Y., Shimba, N., Toyosaki, H., Kawahara, Y., Sugimoto, S., and Suzuki, E. (2004) The energetic conversion competence of *Escherichia coli* during aerobic respiration studied by _31_P NMR using a circulating fermentation system. *J Biochem* **136:** 509–515.

Podolsky, R.J., and Morales, M.F. (1956) The enthalpy change of adenosine triphosphate hydrolysis. *J Biol Chem* **218:** 945–959.

Sauer, U., and Bailey, J.E. (1999) Estimation of P-to-O ratio in *Bacillus subtilis* and its influence on maximum riboflavin yield. *Biotechnol Bioeng* **64:** 750–754.

Schomburg, I., Chang, A., Hofmann, O., Ebeling, C., Ehrentreich, F., and Schomburg, D. (2002) BRENDA: a resource for enzyme data and metabolic information. *Trends Biochem Sci* **27:** 54–56.

Stephanopoulos, G., Aristidou, A., and Nielsen, J. (1998) *Metabolic Engineering: Principles and Methodologies*. San Diego, CA, USA: Academic Press.

von Stockar, U., Gustafsson, L., Larsson, C., Marison, I., Tissot, P., and Gnaiger, E. (1993) Thermodynamic considerations in constructing energy balances for cellular growth. *Biochim Biophys Acta* **1183:** 221–240.

Tatusov, R.L., Fedorova, N.D., Jackson, J.D., Jacobs, A.R., Kiryutin, B., Koonin, E.V., *et al*. (2003) The COG database: an updated version includes eukaryotes. *BMC Bioinformatics* **4:** 41.

Zamboni, N., Maaheimo, H., Szyperski, T., Hohmann, H.P., and Sauer, U. (2004) The phosphoenolpyruvate carboxykinase also catalyzes C3 carboxylation at the interface of glycolysis and the TCA cycle of *Bacillus subtilis*. *Metab Eng* **6:** 277–284.
